# Gut microbiota orchestrates skeletal muscle development and metabolism in germ-free and SPF pigs

**DOI:** 10.3389/fmicb.2025.1615884

**Published:** 2025-06-16

**Authors:** Zhengjie Li, Mingxing Wen, Chuang Tang, Shuangshuang Chen, Die Tang, Jinwei Zhang, Jing Sun, Liangpeng Ge, Keren Long, Lu Lu, Long Jin, Mingzhou Li, Xuewei Li, Jideng Ma

**Affiliations:** ^1^State Key Laboratory of Swine and Poultry Breeding Industry, College of Animal Science and Technology, Sichuan Agricultural University, Chengdu, China; ^2^Chongqing Academy of Animal Sciences, Chongqing, China; ^3^Key Laboratory of Pig Industry Sciences, Ministry of Agriculture, Chongqing, China; ^4^Chongqing Key Laboratory of Pig Industry Sciences, Chongqing, China

**Keywords:** gut microbiota, germ-free (GF) pigs, specific pathogen-free (SPF) pigs, skeletal muscle, LncRNA (long non-coding RNA), muscle fiber typing

## Abstract

The gut microbiota, as a crucial symbiotic microbial community in the host, participates in regulating the host’s metabolism, immunity, and tissue development. Skeletal muscle is a key tissue for movement and energy metabolism in the body, with its development and function regulated by multiple factors; however, the molecular mechanisms by which the gut microbiota influences skeletal muscle remain unclear. This study utilized germ-free (GF) and specific pathogen-free (SPF) pig models, combined with multiple analytical approaches, to systematically investigate the effects of gut microbiota absence on skeletal muscle development, muscle fiber typing, and metabolism. The study found that skeletal muscle development in GF pigs was impaired, with significant changes in muscle fiber diameter and the proportion of type I muscle fibers, with the forelimb extensor digitorum lateralis being the most significantly affected. Metabolic analysis revealed that short-chain fatty acid (SCFA) levels in the muscles of GF pigs were reduced, while amino acid and organic acid levels were elevated, suggesting that the gut microbiota regulates muscle energy metabolism. RNA-seq analysis revealed that the expression levels of protein-coding genes (PCGs) and LncRNAs in the muscles of GF pigs were generally reduced, with LncRNAs exhibiting more pronounced dynamic changes. Differentially expressed genes were enriched in muscle development and immune pathways, with significant changes in the expression patterns of *HOX* and *Homeobox* family genes, myokines, and myosin heavy chain (*MYH*) subtypes. WGCNA analysis identified 16 core genes associated with muscle nutrient metabolism and nine core genes related to muscle fiber phenotypes. Cis-acting LncRNA target gene prediction identified 40 differentially expressed LncRNAs and their regulated 29 PCGs, which are primarily involved in skeletal muscle development and immune responses, suggesting that LncRNAs may influence muscle homeostasis by regulating adjacent genes. In summary, the absence of gut microbiota disrupts skeletal muscle morphogenesis, metabolic characteristics, and transcriptional regulatory networks, with LncRNAs potentially mediating the regulation of muscle-specific genes in this process. This study elucidates the interaction mechanisms between the gut microbiota and skeletal muscle, providing a theoretical foundation and data support for further exploration of the microbiota-muscle axis in pathophysiological contexts.

## Introduction

Muscle growth, development, and metabolic function significantly influence animal growth performance and energy homeostasis (Hargreaves and Spriet, 2020), critically impacting meat production traits and quality. As a key symbiotic microbial community, the gut microbiota regulates host physiological functions through diverse pathways, including metabolites, immune modulation, and endocrine signaling (Delzenne and Cani, 2011; Oliphant and Allen-Vercoe, 2019; Wen et al., 2024). The recently proposed “gut-muscle axis” concept highlights a bidirectional regulatory relationship between the gut microbiota and skeletal muscle (Grosicki et al., 2018; Lahiri et al., 2019). Research indicates that the gut microbiota modulates muscle energy metabolism via short-chain fatty acids (SCFAs) (Okamoto et al., 2019; Walsh et al., 2015), alters host muscle fiber type distribution through microbiota transplantation (Yan et al., 2016; Liu et al., 2017), and enhances muscle quality with probiotic supplementation (Chen et al., 2016; Choi et al., 2015). The gut microbiota affects skeletal muscle function through multiple mechanisms, including metabolic regulation (Walsh et al., 2015; Gao et al., 2009), immune modulation (Ghosh et al., 2015; Thevaranjan et al., 2017), protein metabolism (Atherton and Smith, 2012; Tipton et al., 2018), and neural regulation (Swenarchuk, 2019). Notably, germ-free mouse models have shown that microbiota absence reduces muscle fiber diameter and motor function (Lahiri et al., 2019; Hsu et al., 2015); however, further research is needed in pigs, which are physiologically closer to humans.

Germ-free (GF) animal models provide a unique tool for elucidating microbiota-host interactions. Compared to rodents, GF pigs exhibit high similarity to humans in gastrointestinal anatomy, metabolic characteristics, and immune development (Xiao et al., 2016), with muscle tissue development patterns more closely resembling those of humans (Heinritz et al., 2013; Shirkey et al., 2006). However, existing research primarily focuses on rodent models, and systematic studies on the impact of gut microbiota on skeletal muscle development in large mammals, particularly GF pig models, remain limited. Transcriptomic studies can systematically reveal the effects of microbiota absence on muscle gene expression networks (Lahiri et al., 2019), particularly the regulatory role of long non-coding RNAs (LncRNAs) in muscle development, which remains to be explored (Li et al., 2018).

This study is the first to utilize a self-established germ-free (GF) Bama pig model, combined with multi-omics technologies, to systematically analyze the impact of microbiota absence on skeletal muscle development. By comparing the morphological characteristics, nutrient metabolic profiles, and transcriptomic differences of 14 muscle sites between GF and specific pathogen-free (SPF) pigs, this study focuses on elucidating: (1) the muscle site-specific effects of microbiota absence on muscle fiber type differentiation and nutrient metabolism; (2) the role of key signaling pathways (e.g., *HOX* gene family, *MYH* subtypes) in microbiota-muscle regulation; and (3) LncRNA-mediated transcriptional regulatory networks. The findings will provide new insights into the molecular mechanisms of the “gut-muscle axis” and offer a theoretical basis for improving muscle quality in livestock and poultry.

## Materials and methods

### Ethics approval

This study was approved by the Experimental Animal Ethics Committee, Chongqing Academy of Animal Science, under reference number XKY-No. 20210606.

### Experimental animal samples

This study was conducted at the standardized germ-free swine breeding platform of the Bioengineering Institute, Chongqing Academy of Animal Sciences. Neonatal germ-free piglets were obtained through aseptic cesarean sections performed on strictly screened specific pathogen-free (SPF) healthy pregnant Bama sows. The full-sibling female piglets from the same litter were randomly assigned to GF (germ-free, *n* = 12) and SPF (*n* = 12) groups. The GF group was maintained in sterile isolators throughout the experiment, while the SPF group received oral administration of parental SPF porcine fecal microbiota suspension (1 mL/day for three consecutive days) at 7 days of age to establish microbial colonization. All experimental animals were housed in sterile isolators under identical environmental conditions, with daily disinfection using 2% peracetic acid solution. The germ-free status was routinely monitored through weekly anaerobic culturing of rectal swabs, and subsequently confirmed by 16S rDNA sequencing technology at 44 days of age.

At 45 days of age, three randomly selected animals from each group were fasted for 12 h prior to euthanasia via isoflurane anesthesia followed by cervical exsanguination. Under strict aseptic conditions within a laminar flow hood, muscle tissue samples were collected from 14 anatomical regions: including masseter muscle (head), brachial head muscle, triceps brachii, flexor digitorum profundus, and extensor digitorum lateralis (forelimbs), longissimus dorsi muscle, rectus abdominis, and pectoralis profundus (trunk), as well as psoas major muscle, biceps femoris, gastrocnemius muscle, soleus muscle, medial femoral muscle, and adductores (hindlimbs). The collected muscle specimens were categorized into three processing groups according to experimental requirements: (1) RNA-seq samples: Rinsed with normal saline, blotted with sterile filter paper, and immediately flash-frozen in liquid nitrogen; (2) Cryosection samples: Embedded in OCT compound prior to liquid nitrogen freezing; (3) Nutritional analysis samples: Directly frozen in liquid nitrogen after saline rinsing. All processed specimens were stored as triplicated aliquots at −80°C to ensure experimental reproducibility and data reliability.

### Assessment of muscle nutrient-related indices

This study systematically analyzed the nutritional metabolic characteristics of muscle tissues. The measured parameters encompassed five major categories: amino acids, short-chain fatty acids (SCFAs), organic acids, free fatty acids (FFAs), and total fatty acids. The detailed experimental procedures were as follows: frozen muscle samples stored at −80°C were pulverized into homogeneous powder using liquid nitrogen-prechilled mortars. Precisely 100 mg aliquots were weighed into 1.5 mL microcentrifuge tubes, with all samples maintained on dry ice during transport to Beijing Hexin Technology Co., Ltd. for subsequent analyses.

### Cryosectioning and ATPase histochemistry

The muscle tissue sectioning protocol was performed as follows: (1) 15-μm sections were obtained using a cryostat at −17°C; (2) Sections were equilibrated at room temperature for 5 min; (3) Incubated in acidic solution (pH 4.55; 0.97 g sodium acetate + 1.47 g barbital sodium + 100 mL 0.1 M HCl) with 100 rpm shaking for 5 min; (4) Rinsed in alkaline buffer (pH 9.6; 40 mL 0.1 M barbital sodium + 20 mL 0.18 M anhydrous calcium chloride + 140 mL ddH₂O) with shaking for 1 min; (5) ATPase reaction solution (pH 9.6; 40 mL 0.1 M barbital sodium + 500 mg ATP + 20 mL 0.18 M CaCl₂ + 140 mL ddH₂O) with 80 min incubation; (6) 1% calcium chloride solution (2 g anhydrous CaCl₂ + 200 mL ddH₂O) with 10 min incubation; (7) 3% calcium chloride solution (4 g anhydrous CaCl₂ + 200 mL ddH₂O) with 3 min incubation; (8) Washed 8 times (30–60 s each) in 0.01 M barbital sodium solution (0.4 g + 200 mL ddH₂O); (9) Rinsed under running tap water for 2–3 min; (10) 1% ammonium sulfide solution (10 mL 20% stock + 190 mL ddH₂O) with 1 min incubation; (11) Thoroughly rinsed with tap water for 10 min; (12) Dehydrated in xylene and mounted with Canada balsam; (13) Imaging performed using confocal microscopy; (14) Fiber type-specific parameters (diameter/cross-sectional area) analyzed using Image-Pro Plus 6.0.

### Total RNA extraction

Total RNA was isolated from muscle tissues using the HiPure Universal RNA Mini Kit (Magen, China). The detailed procedures were performed as follows: 10–60 mg tissue aliquots were homogenized in 1 mL MagZol Reagent. Homogenized samples were incubated at room temperature for 5–10 min. After adding 200 μL chloroform with vigorous vortexing (15 s), phase separation was achieved by centrifugation at 12,000 × g for 15 min at 4°C following 3 min incubation. The aqueous phase was mixed with 1.5 volumes of absolute ethanol and loaded onto HiPure RNA Mini Columns (70 s centrifugation). Two washes with 600 μL Buffer RW2 were performed, and RNA was eluted with 60 μL RNase-free water. RNA quality was assessed using NanoDrop 2000 spectrophotometer (Thermo Scientific) and stored at −80°C for downstream applications.

### RNA library preparation and sequencing

Library construction and sequencing were performed by Novogene Bioinformatics Technology Co., Ltd. (Beijing, China). RNA integrity was rigorously assessed using Agilent 2100 Bioanalyzer (RIN ≥7.0). Qualified samples were processed with NEBNext^®^ Ultra^™^ Directional RNA Library Prep Kit for Illumina^®^ (NEB, United States) through the following workflow: (1) rRNA depletion and mRNA enrichment; (2) Random fragmentation of mRNA using divalent cations in NEB Fragmentation Buffer; (3) First-strand cDNA synthesis with M-MuLV reverse transcriptase using fragmented mRNA as template, followed by RNA strand degradation with RNase H and second-strand synthesis using dUTP-marked DNA polymerase I system; (4) Purified dsDNA underwent end repair, A-tailing and adaptor ligation; (5) Size selection (250–300 bp) using AMPure XP beads (Beckman Coulter, United States); (6) USER enzyme digestion of U-containing second strand followed by PCR amplification.

Library QC included: (i) Qubit 2.0 quantification (diluted to 1.5 ng/μL), (ii) Insert size verification via Agilent 2100 Bioanalyzer, and (iii) Accurate concentration determination by RT-qPCR (KAPA Biosystems). Validated libraries were pooled based on effective concentration and data requirements for paired-end sequencing (150 bp) on Illumina platforms.

### Sequence alignment, transcript identification and quantification

Raw sequencing data were processed by removing reads containing adapter contamination (>5 bp), low-quality bases (*Q* ≤ 19) accounting for >50% of total bases, or ambiguous bases (*N* ratio >5%), yielding high-quality clean data. We employed the published *Sus scrofa* genome assembly and annotation data (Ensembl release 102). Quality-filtered reads were aligned to the reference genome using STAR (v2.7.10a) (Dobin et al., 2012) with default parameters, and mapping rates were calculated. Transcript annotation integrated comprehensive information including protein-coding genes (PCGs), long non-coding RNAs (LncRNAs), and transcripts of uncertain coding potential (TUCPs). Transcript abundance was precisely quantified using kallisto (v0.46.2) (Bray et al., 2016) in pseudoalignment mode. Both raw read counts and transcripts per million (TPM) values were obtained for downstream differential expression analysis. This analytical pipeline ensured the accuracy and reproducibility of transcriptomic quantification.

### Differential gene expression analysis

Gene-level expression quantification was performed using Tximport (R package) to obtain normalized expression values in transcripts per million (TPM). Expressed genes were defined as those meeting the following criteria: TPM ≥1 for PCGs, TPM ≥0.1 for LncRNAs/TUCPs, and detectable in at least three biological replicates. Differential expression analysis was conducted using DESeq2 (v1.38.3) (Love et al., 2014) with negative binomial distribution modeling. The significance thresholds were set at adjusted *p*-value ≤0.05 (Benjamini–Hochberg correction) and absolute log2 fold change ≥1. Protein-coding genes, LncRNAs and TUCPs satisfying these criteria were identified as differentially expressed genes (DEGs).

### Functional enrichment analysis

To investigate potential regulatory relationships between differentially expressed PCGs and LncRNAs, Pearson correlation coefficients were calculated using Hmisc R package (v4.7-2). Gene pairs with absolute Pearson’s *r* ≥ 0.9 and adjusted *p*-value ≤0.05 were identified as candidate regulatory pairs. Functional enrichment analysis of selected PCGs was performed using Metascape (2023 update) (Zhou et al., 2019), with particular focus on muscle development-related biological processes in GO terms (cellular component, molecular function, and biological process). Visualization was implemented with ggplot2 (v3.4.2) to generate publication-quality figures.

### LncRNA target gene prediction

This study employed dual-strategy approaches for LncRNA target prediction: (1) Cis-regulation analysis: Protein-coding genes within 100 kb upstream/downstream of LncRNAs were considered potential targets; (2) Trans-regulation analysis: Regulatory relationships were identified by Pearson correlation (absolute *r* > 0.7, *p* < 0.05) between differentially expressed LncRNAs and PCGs. An integrated LncRNA-PCG regulatory network was constructed by combining both prediction methods using Cytoscape (v3.9.1) (Shannon et al., 2003). Systematic functional enrichment analysis (GO and KEGG) was performed on predicted target genes using clusterProfiler (v4.6.2) (Yu et al., 2012) to elucidate potential regulatory mechanisms of LncRNAs in muscle development.

### Weighted gene co-expression network analysis

The weighted gene co-expression network was constructed using the WGCNA R package (v1.72). The analytical pipeline included: (1) Construction of a gene–gene similarity matrix across all samples; (2) Determination of optimal soft-thresholding power (*β* = 7) to achieve scale-free topology (*R*^2^ > 0.85); (3) Module detection via dynamic tree cutting (minModuleSize = 30, mergeCutHeight = 0.15); (4) Identification of key modules by correlating module eigengenes with phenotypic traits (Pearson’s *r*, *p* < 0.05). Network topology was characterized by: (i) Module membership (MM) quantifying gene-module correlation (−1 to 1), and (ii) Gene significance (GS) measuring gene-phenotype associations. Hub genes were selected using stringent criteria (GS >0.8 & absolute MM >0.8), representing highly connected nodes with significant phenotypic relevance. These molecular hubs provide prioritized targets for mechanistic studies of muscle development regulation.

### Muscle fiber typing via bulk deconvolution

This study integrated snRNA-seq data from Bama pig longissimus dorsi (unpublished dataset). A systematic analytical pipeline was implemented to decipher myonuclear heterogeneity: (1) snRNA-seq quantification using CellRanger (10× Genomics, v7.1.0); (2) Data normalization and processing via Seurat (v4.3.0) including: highly variable gene selection, PCA dimensionality reduction, and UMAP nonlinear clustering for unsupervised cell clustering; (3) Identification of three myonuclear subtypes using canonical markers: type I (slow oxidative), type IIA (fast oxidative-glycolytic), and type IIB (fast glycolytic).

For bulk transcriptome analysis: (1) CIBERSORTx deconvolution was performed using a signature matrix derived from snRNA-seq subtype profiles; (2) Bulk RNA-seq data were deconvolved with 1,000 permutations, yielding relative proportions of each myonuclear subtype with *p* < 0.05 confidence. This approach enabled precise quantification of fiber-type dynamics during muscle development.

### Statistical analysis

A multi-platform analytical framework was implemented: (1) Morphometric analysis (fiber diameter/cross-sectional area) using Image-Pro Plus 6.0 (Media Cybernetics) with manual quality control; (2) Data visualization with: (i) GraphPad Prism 9.0 for statistical graphics following Nature journal style guidelines, and (ii) Cytoscape 3.9.1 for gene interaction networks (edge-weighted by correlation coefficients); (3) Statistical tests: (i) Two-group comparisons: unpaired Student’s *t*-test (two-tailed), and (ii) Multi-group comparisons: One-way ANOVA with post-hoc Tukey test (FDR correction). Data are presented as mean ± SEM from ≥3 biological replicates. Significance levels: ^*^p < 0.05 and ^**^p < 0.01, with exact *p*-values reported in figure legends.

## Results

### Establishment of GF and SPF porcine models with distinct muscle morphological phenotypes

This study strictly adhered to the Chinese National Standard (GB/T 14926-41-2001), employing dual detection methods to ensure the establishment of the GF pig model. Environmental, feed, and piglet samples were collected weekly and cultured in anaerobic (thioglycolate medium) and aerobic (brain-heart infusion broth) media for monitoring. Experimental results (Supplementary Figure 1A) showed no bacterial growth in any samples from GF pigs. 16S rDNA sequencing analysis further confirmed that the Shannon index and observed feature index of rectal samples from GF pigs were significantly lower than those from SPF pigs (*p* < 0.05), with no significant differences compared to sterile feed and milk powder (Supplementary Figures 1B,C). These results robustly demonstrate that this study successfully established GF and SPF pig models that meet the required standards.

**Figure 1:**
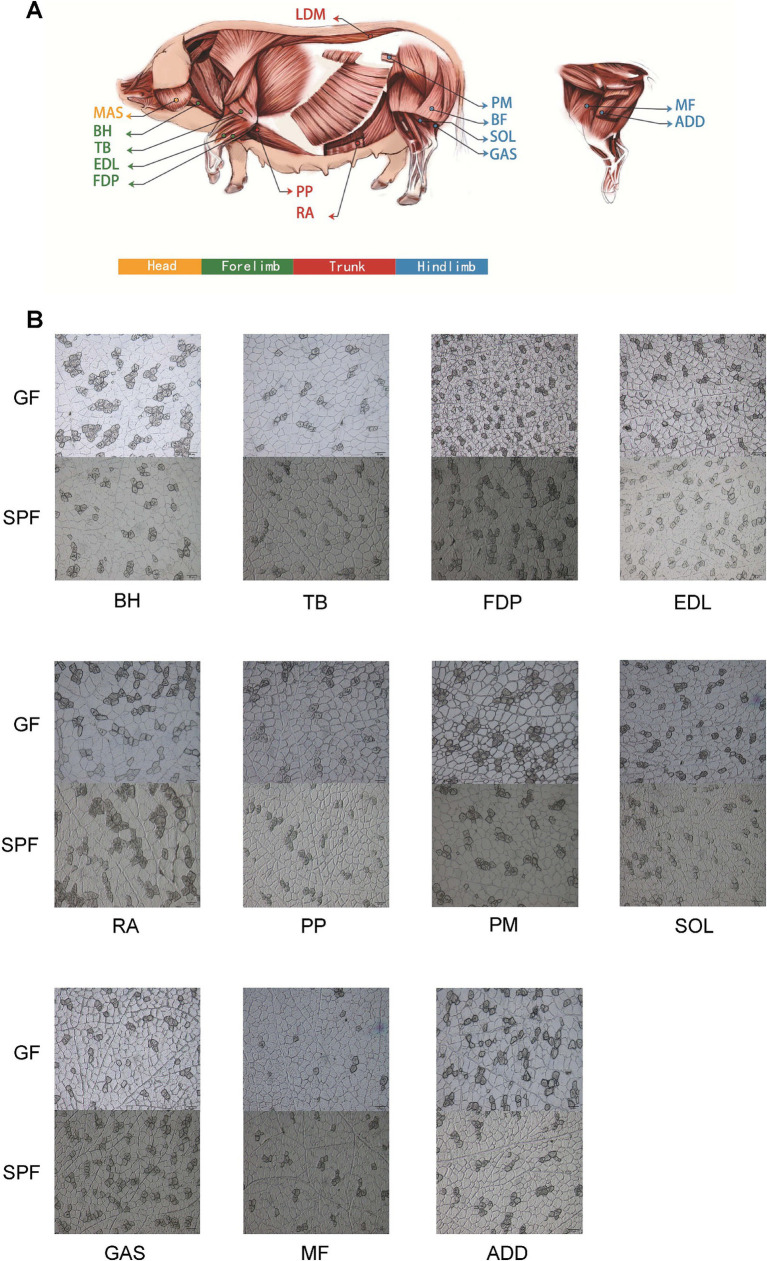
Sampling distribution and ATPase staining analysis of porcine skeletal muscle tissues. **(A)** Schematic diagram of skeletal muscle tissue sampling sites. **(B)** ATPase staining of muscle tissues from SPF and GF pigs (scale bar: 50 μm). BH, brachial head; TB, triceps brachii; FDP, flexor digitorum profundus; EDL, extensor digitorum lateralis; RA, rectus abdominis; PP, pectoralis profundus; PM, psoas major; SOL, soleus; GAS, gastrocnemius; MF, medial femoral; ADD, adductores.

Using frozen sectioning and ATPase staining techniques, we systematically analyzed the muscle fiber characteristics of 14 muscle tissues from GF and SPF pigs (Figure 1A) (Jin et al., 2021). The results revealed significant differences in the diameter, area, and number of 13 muscle fiber types, indicating that the absence of gut microbiota significantly impacts skeletal muscle development and muscle fiber typing. These findings provide critical experimental evidence for a deeper understanding of the regulatory mechanisms of the “gut-muscle axis.”

The results (Supplementary Table 1 and Figure 1B) demonstrate that the absence of gut microbiota significantly impacts skeletal muscle development and muscle fiber typing. Compared to SPF pigs, GF pigs exhibited smaller muscle fiber diameters in 10 muscle tissues, with the extensor digitorum lateralis showing a significant reduction (24.12 ± 1.12 μm vs. 30.85 ± 3.84 μm, *p* < 0.05). Regarding muscle fiber typing, the diameters of type I and type II muscle fibers in nine muscle tissues of GF pigs were smaller than those in SPF pigs, with type I fibers in the pectoralis profundus and extensor digitorum lateralis (25.25 ± 1.68 μm and 20.97 ± 3.94 μm) being significantly smaller than those in SPF pigs (27.16 ± 4.86 μm and 30.23 ± 3.80 μm, *p* < 0.05), and type II fibers in the extensor digitorum lateralis (24.81 ± 1.05 μm) being highly significantly smaller than those in SPF pigs (31.52 ± 4.2 μm, *p* < 0.01).

Interestingly, the area and proportion of type I muscle fibers in six hindlimb muscle tissues of GF pigs were significantly reduced, with the extensor digitorum lateralis showing a reduction of nearly 50%. However, the proportion of type I muscle fibers in the remaining seven muscle tissues showed no significant changes. This indicates that the impact of gut microbiota on skeletal muscle is tissue-specific, with the most pronounced effects observed in the forelimb extensor digitorum lateralis and the trunk pectoralis profundus.

Overall, the absence of gut microbiota has a significantly greater impact on skeletal muscle development than on muscle fiber typing. These findings provide critical evidence for understanding the regulatory mechanisms of the “gut-muscle axis,” suggesting that the gut microbiota may regulate skeletal muscle growth, development, and fiber type transformation through specific pathways.

### Gut microbiota deficiency disrupts muscle nutrient composition

Based on the phenotypic differences in muscle tissues between GF and SPF pigs, we selected six muscle sites—masseter, triceps brachii, pectoralis profundus, rectus abdominis, psoas major, and gastrocnemius—for metabolic profiling. Ultra-performance liquid chromatography (UPLC) analysis revealed that short-chain fatty acid (SCFA) levels in five muscle tissues of GF pigs were generally lower than those in SPF pigs (Supplementary Table 2). Notably, acetate (3.135 ± 0.070 vs. 5.457 ± 0.348 μg/g, *p* < 0.01) and butyrate (0.129 ± 0.013 vs. 0.174 ± 0.012 μg/g, *p* < 0.05) levels in the masseter were significantly and highly significantly reduced, respectively. Propionate levels in the psoas major (0.115 ± 0.037 vs. 0.344 ± 0.116 μg/g) were significantly reduced (*p* < 0.05). Valeric acid levels in the psoas major (0.011 ± 0.002 vs. 0.024 ± 0.003 μg/g) and gastrocnemius (0.012 ± 0.002 vs. 0.022 ± 0.003 μg/g) were highly significantly reduced (*p* < 0.01). Notably, SCFA levels in the pectoralis profundus showed an opposite trend, with propionate (0.328 ± 0.036 vs. 0.124 ± 0.031 μg/g) and valeric acid (0.023 ± 0.004 vs. 0.007 ± 0.001 μg/g) levels highly significantly elevated compared to SPF pigs (*p* < 0.01). These findings suggest that the absence of gut microbiota modulates skeletal muscle function by altering SCFA metabolism, with pronounced tissue-specific effects.

**Figure 2:**
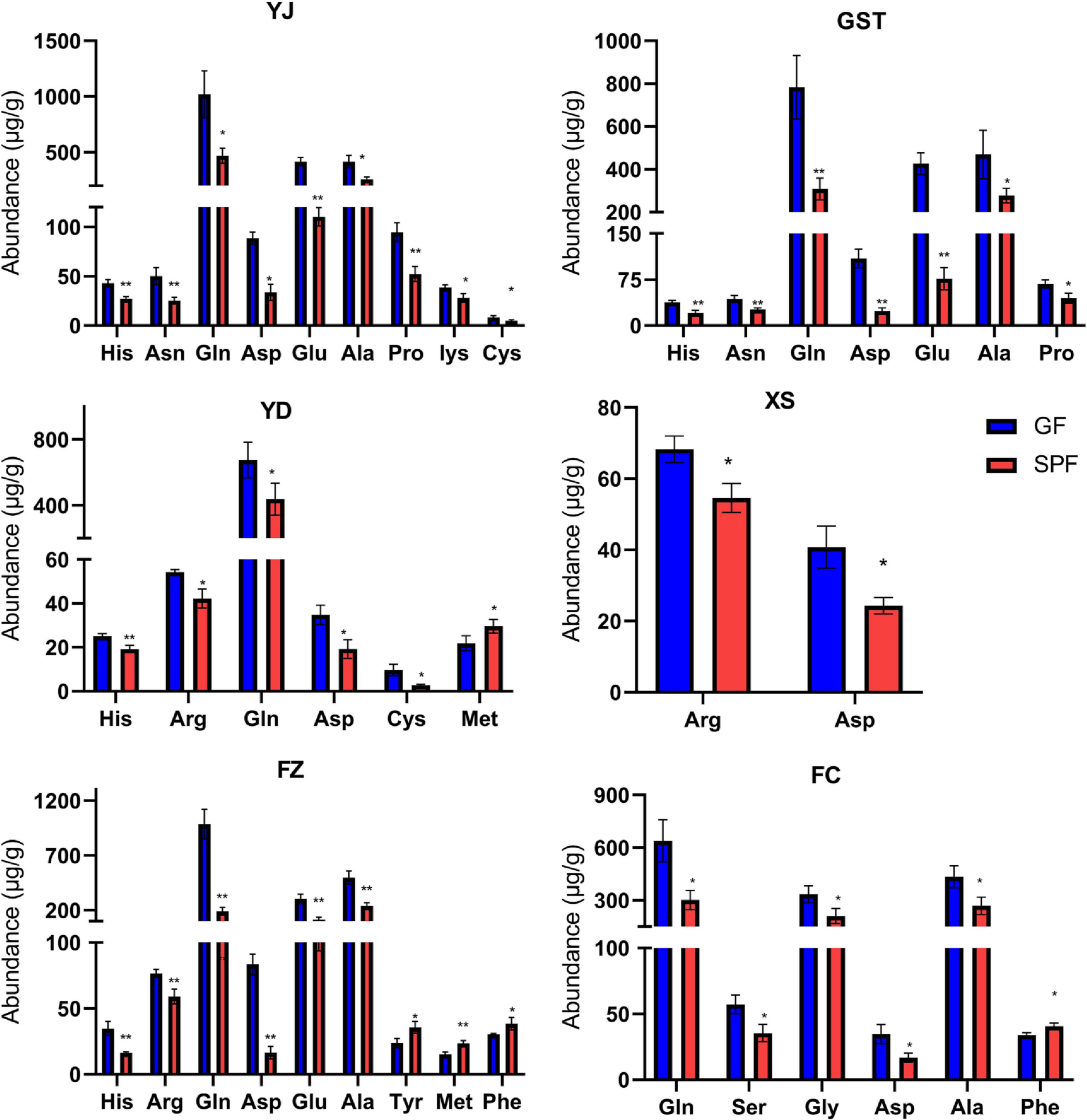
Amino acid content in muscle tissues from different anatomical sites of SPF and GF pigs. YJ (MAS, masseter), GST (TB, triceps brachii), YD (PM, psoas major), XS (PP, pectoralis profundus), FZ (RA, rectus abdominis), FC (GAS, gastrocnemius).

Analysis using the Waters ACQUITY UPLC I-CLASS system revealed that most amino acid levels in GF pig muscle tissues were significantly elevated compared to SPF pigs, with histidine, glutamine, serine, aspartic acid, glutamic acid, threonine, lysine, and valine showing particularly pronounced increases, and cystine detected exclusively in the pectoralis profundus of GF pigs. Specifically, histidine levels were highly significantly elevated in the masseter, triceps brachii, psoas major, and rectus abdominis of GF pigs compared to SPF pigs (*p* < 0.01). Arginine levels were significantly increased in the pectoralis profundus and psoas major (*p* < 0.05) and highly significantly increased in the rectus abdominis (*p* < 0.01). Glutamine levels were significantly elevated in the masseter, psoas major, and gastrocnemius (*p* < 0.05) and highly significantly elevated in the triceps brachii and rectus abdominis (*p* < 0.01). Aspartic acid levels were significantly increased in the masseter, pectoralis profundus, psoas major, and gastrocnemius (*p* < 0.05) and highly significantly increased in the triceps brachii and rectus abdominis (*p* < 0.01). Lysine and cystine levels were significantly elevated in the masseter and psoas major (*p* < 0.05). Methionine levels were significantly increased in the psoas major (*p* < 0.05) and highly significantly increased in the rectus abdominis (*p* < 0.01). These findings indicate that the absence of gut microbiota in GF pigs disrupts amino acid metabolism in muscle tissues, resulting in generally elevated amino acid levels (Figure 2 and Supplementary Table 3).

Targeted analysis of 16 organic acids using the Waters ACQUITY UPLC I-CLASS system detected 14 organic acids in the muscle tissues of GF and SPF pigs, with phenylacetic acid and ethylmalonic acid undetectable. The results (Supplementary Table 4) showed that most organic acid levels were higher in GF pig muscle tissues compared to SPF pigs, with pyruvate, fumaric acid, malic acid, and oxaloacetic acid exhibiting particularly significant differences. Notably, succinic acid was detected only in the triceps brachii of GF pigs (undetectable in SPF pigs) and was absent in the rectus abdominis and gastrocnemius of both groups.

**Figure 3:**
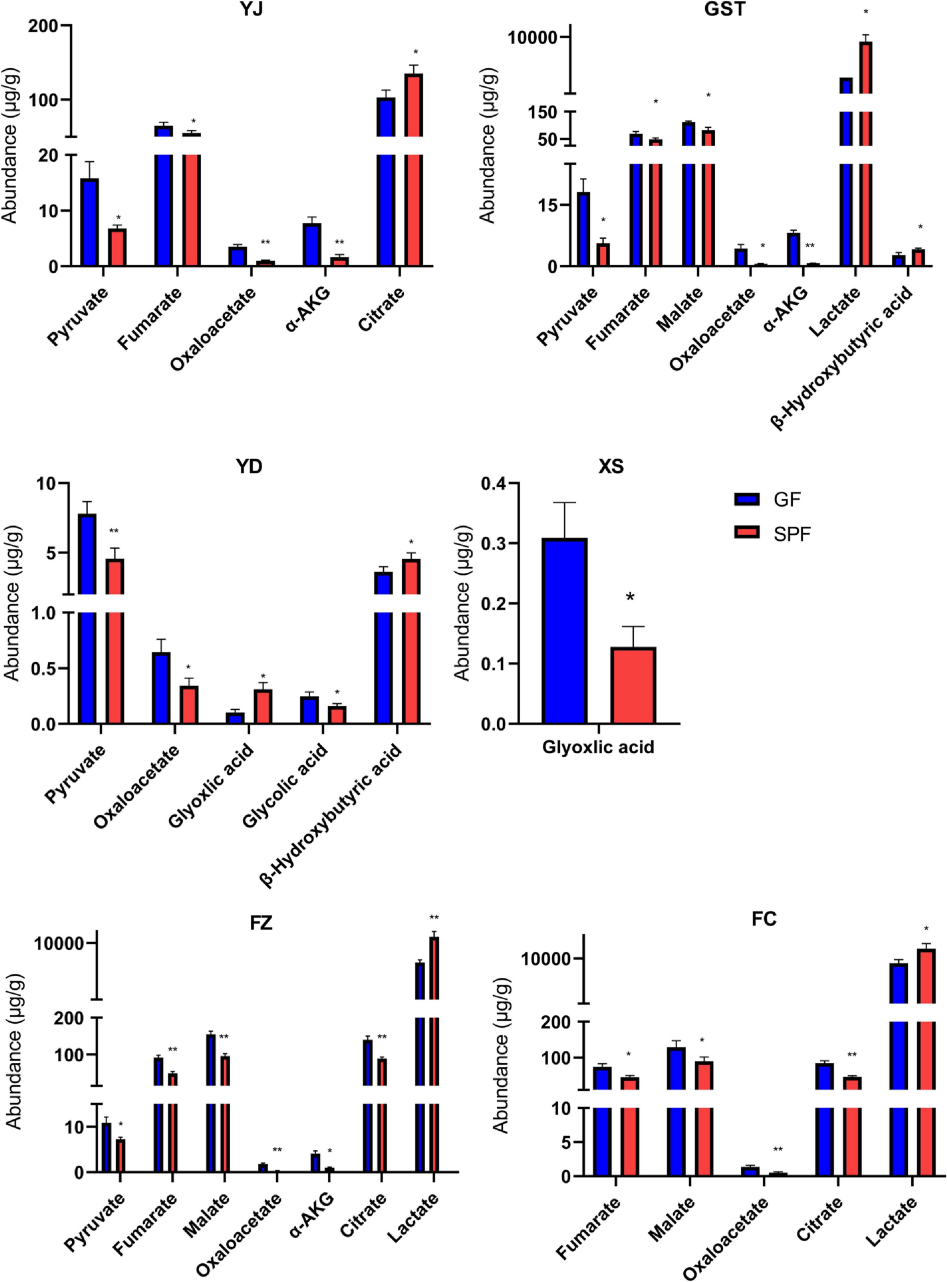
Organic acid content in muscle tissues from different anatomical sites of SPF and GF pigs. YJ (MAS, masseter), GST (TB, triceps brachii), YD (PM, psoas major), XS (PP, pectoralis profundus), FZ (RA, rectus abdominis), FC (GAS, gastrocnemius).

Specifically (Figure 3), pyruvate levels were significantly elevated in the masseter, triceps brachii, and rectus abdominis of GF pigs (*p* < 0.05) and highly significantly elevated in the psoas major (*p* < 0.01). Fumaric acid levels were significantly increased in the masseter, triceps brachii, and gastrocnemius (*p* < 0.05) and highly significantly increased in the rectus abdominis (*p* < 0.01). Malic acid levels were significantly elevated in the triceps brachii and gastrocnemius (*p* < 0.05) and highly significantly elevated in the rectus abdominis (*p* < 0.01). Oxaloacetic acid levels were highly significantly increased in the masseter, rectus abdominis, and gastrocnemius (*p* < 0.01) and significantly increased in the triceps brachii and psoas major (*p* < 0.05). *α*-Ketoglutaric acid levels were highly significantly elevated in the masseter and triceps brachii (*p* < 0.01) and significantly elevated in the rectus abdominis (*p* < 0.05). Citric acid levels were significantly reduced in the masseter (*p* < 0.05) but highly significantly increased in the rectus abdominis and gastrocnemius (*p* < 0.01). Lactic acid and β-hydroxybutyric acid levels were significantly or highly significantly reduced in multiple muscle sites, while glyoxylic acid and glycolic acid showed differential changes in specific muscles. These findings indicate that the absence of gut microbiota significantly alters organic acid metabolism in GF pig muscle tissues, resulting in generally elevated levels of most organic acids.

Using the Waters ACQUITY UPLC I-CLASS system, 37 free fatty acids were analyzed, with 34 detected in the muscle tissues of GF and SPF pigs. The results (Supplementary Table 5) revealed tissue-specific variations in free fatty acid levels across different muscle tissues of GF pigs. Specifically, free fatty acid levels were generally higher in the triceps brachii (31/34) and rectus abdominis (34/34) of GF pigs compared to SPF pigs, but lower in the pectoralis profundus (33/34) and psoas major (29/34). Notably, FA22:1 levels were consistently higher in all tested muscle tissues of GF pigs compared to SPF pigs.

Specifically (Supplementary Figure 2A), in the rectus abdominis, levels of FA11:0, FA16:0, FA16:1, and FA18:2 were highly significantly elevated (*p* < 0.01), while levels of FA18:0, FA18:3, FA20:5, FA23:0, and FA24:0 were significantly elevated (*p* < 0.05). Conversely, FA20:2 levels were significantly reduced in the pectoralis profundus (*p* < 0.05). These findings indicate that the absence of gut microbiota induces pronounced tissue-specific differences in free fatty acid metabolism in GF pig muscle tissues, suggesting varying degrees of responsiveness to gut microbiota-mediated metabolic regulation across different muscles.

Using the Waters ACQUITY UPLC I-CLASS system, 36 fatty acids (saturated fatty acids, monounsaturated fatty acids, polyunsaturated fatty acids) were analyzed, with 31 detected in the muscle tissues of GF and SPF pigs (Supplementary Table 6). The results showed that most fatty acid levels were lower in the triceps brachii (24/31), rectus abdominis (27/31), and gastrocnemius (21/31) of GF pigs compared to SPF pigs, but generally higher in the pectoralis profundus (24/31) and psoas major (29/31). Notably, levels of PUFA C18:3n6 and C22:6 were consistently higher in all tested muscle tissues of GF pigs compared to SPF pigs, whereas levels of SFA C15:0, C17:0, and MUFA C17:1 were generally lower (Supplementary Figure 2B).

Specifically, SFA C8:0 levels were highly significantly reduced in the triceps brachii (*p* < 0.01) but highly significantly elevated in the pectoralis profundus (*p* < 0.01). MUFA C16:1 levels were highly significantly reduced in the triceps brachii (*p* < 0.01) but highly significantly elevated in the pectoralis profundus (*p* < 0.01). PUFA C18:3n3 levels were significantly reduced in the triceps brachii (*p* < 0.05) but highly significantly elevated in the gastrocnemius (*p* < 0.01). C24:1 was undetectable in the masseter. These findings indicate that the absence of gut microbiota induces pronounced tissue-specific differences in fatty acid metabolism in GF pig muscle tissues, with short-chain fatty acids generally reduced and PUFA C18:3n6 and C22:6 consistently elevated, suggesting tissue-specific regulation of fatty acid metabolism by the gut microbiota.

### Transcriptomic profiling reveals gut microbiota-dependent regulation of skeletal muscle development

Sequencing results (Supplementary Table 7) showed that 84 libraries yielded a total of 7526.52 million paired-end 150 nt raw reads, with an average of 89.60 ± 4.35 million reads per library. The GF pig group (3757.83 million reads, 89.47 ± 3.52 million reads per library) and the SPF pig group (3768.69 million reads, 89.73 ± 5.09 million reads per library) generated comparable data volumes. The total data volume reached 1,129 GB (13 ± 0.65 GB per library), with the GF pig group (563.69 GB, 13.42 ± 0.53 GB per library) and the SPF pig group (565.31 GB, 13.45 ± 0.76 GB per library) showing similar outputs. After quality control filtering, 97.36% of raw reads were retained as high-quality clean reads, with the GF pig group (97.85%) slightly outperforming the SPF pig group (96.81%).

Sequencing quality assessment (Supplementary Table 8) revealed that the sequencing error rates of 84 libraries ranged from 0.02 to 0.04%, with GF pig libraries generally exhibiting lower error rates than SPF pig libraries (only SPF6-GNC reached 0.04%). All libraries achieved Q20/Q30 values above 90% (except SPF6-GNC at 85.86%), with the highest single-base error rate below 0.07%. GC content analysis showed that 83 libraries had stable GC content around 50%, with only SPF6-GNC exhibiting abnormal segregation. These results indicate high-quality sequencing data with accurate base calling. Genome alignment results (Supplementary Table 9) demonstrated that the average genome mapping rate across 84 libraries exceeded 97%, with unique mapping rates above 86%, confirming high-quality sequencing data suitable for downstream bioinformatics analysis.

**Figure 4:**
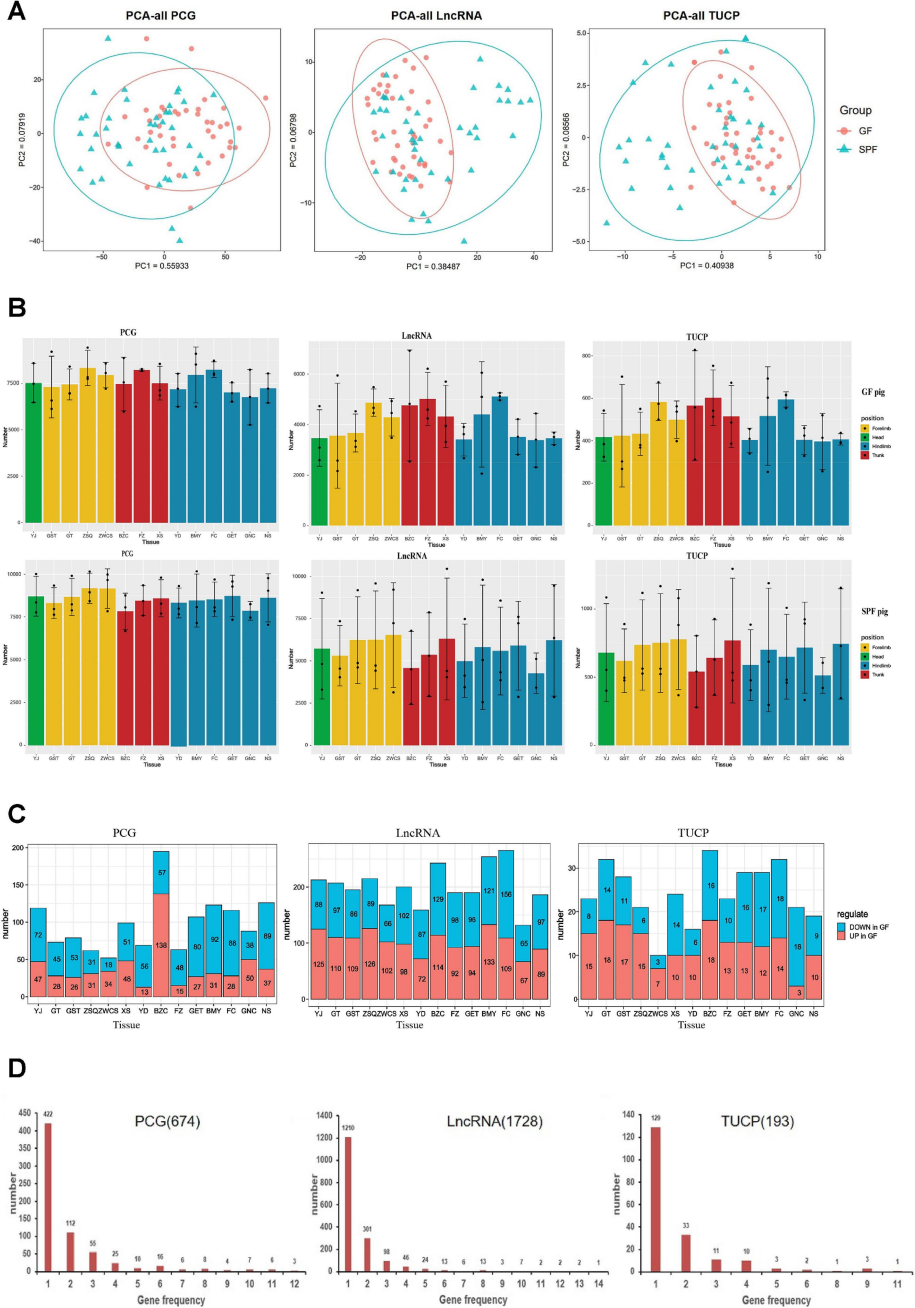
Transcriptomic characterization of PCG, LncRNA, and TUCP in muscle tissues of GF and SPF pigs. **(A)** Principal component analysis of PCG, LncRNA, and TUCP expression levels in muscle tissues of GF and SPF pigs. **(B)** Expression profiles of three transcript types in different muscle tissues of GF and SPF pigs. **(C)** Number of differentially expressed PCGs, LncRNAs, and TUCPs (up- and downregulated) in different muscle tissues of GF and SPF pigs. **(D)** Frequency of differentially expressed PCGs, LncRNAs, and TUCPs across 14 muscle tissues in GF and SPF pigs. YJ (MAS, masseter muscle), GST (TB, triceps brachii), GT (BH, brachial head muscle), ZSQ (FDP, flexor digitorum profundus), ZWCS (EDL, extensor digitorum lateralis), BZC (LDM, longissimus dorsi muscle), FZ (RA, rectus abdominis), XS (PP, pectoralis profundus), YD (PM, psoas major muscle), BMY (SOL, soleus muscle), FC (GAS, gastrocnemius muscle), GET (BF, biceps femoris), GNC (MF, medial femoral muscle), and NS (ADD, adductores).

Transcriptome correlation analysis (Supplementary Figure 3) showed that Pearson correlation coefficients based on PCG, LncRNA, and TUCP expression levels failed to effectively distinguish between GF and SPF pigs, with high correlations observed among different muscle tissues. However, PCA analysis (Figure 4A) revealed that PC1 significantly differentiated the two groups, indicating a substantial regulatory effect of gut microbiota on the expression of all three transcript types and confirming the reliability of the transcriptome data.

Transcriptome differential analysis (Supplementary Figure 4A) revealed significant differences in the expression profiles of PCG, LncRNA, and TUCP across 14 muscle tissues between GF and SPF pigs. Co-expression analysis indicated that over 15,000 PCGs (TPM >1) were expressed, with the pectoralis profundus exhibiting the highest number (15,683 PCGs), including 604 GF-specific and 509 SPF-specific PCGs. Over 11,000 LncRNAs (TPM >0.1) were co-expressed, with the pectoralis profundus showing the highest number (13,151 LncRNAs), including 1,334 GF-specific LncRNAs. Over 1,700 TUCPs (TPM >0.1) were co-expressed, with the pectoralis profundus having the highest number (1,923 TUCPs). The expression levels of all transcript types were generally lower in GF pigs compared to SPF pigs, independent of muscle anatomical location (Figure 4B), confirming that gut microbiota absence significantly impacts muscle tissue transcriptional regulation. These differentially expressed genes may be involved in the regulation of the gut-muscle axis, with underlying mechanisms warranting further investigation.

The tissue specificity index (*τ*) ranges from 0 to 1, with higher *τ* values indicating greater tissue specificity and lower *τ* values suggesting lower tissue specificity, resembling housekeeping gene characteristics. We calculated the *τ* values for PCG, LncRNA, and TUCP transcripts (Supplementary Figure 4B). In both GF and SPF pigs, PCG transcripts exhibited the lowest overall *τ* values, followed by TUCP, with LncRNA showing the highest *τ* values. This indicates that LncRNA and TUCP have higher tissue specificity than PCG in skeletal muscle, consistent with previous studies reporting elevated tissue specificity of LncRNA. Compared to SPF pigs, the tissue specificity of PCG transcripts in GF pig muscle tissues was minimally affected. LncRNAs play a role in regulating gene expression, and the overall *τ* values of LncRNA were lower in GF pigs compared to SPF pigs, suggesting differences in gene expression regulation between GF and SPF pigs, likely due to the absence of gut microbiota.

Transcript abundance analysis (Supplementary Figures 4C,D) revealed significant differences in the distribution patterns of different transcript types in muscle tissues between GF and SPF pigs. In GF pigs, the proportion of highly abundant transcripts (top 0.5%) was markedly higher than in SPF pigs (TUCP: 70% vs. 65%; LncRNA: 65% vs. 62.5%; PCG: 53% vs. 50%). These results suggest that the absence of gut microbiota reduces transcriptional complexity and weakens gene expression responses in GF pig muscle tissues. These findings indicate that gut microbiota may play a critical role in maintaining transcriptional diversity and functional complexity in skeletal muscle.

Transcriptome differential analysis (Figure 4C) revealed 674 differentially expressed PCGs, 1,728 LncRNAs, and 193 TUCPs across 14 muscle tissues between GF and SPF pigs, with LncRNA showing the most pronounced changes, further confirming the significant impact of gut microbiota on transcriptional regulation. Regional distribution analysis indicated a clear gradient in the number of differentially expressed genes: head (PCG: 119; LncRNA: 213; TUCP: 23) < forelimb (266/785/91) < trunk (354/633/81) < hindlimb (629/1,186/146), suggesting a more pronounced effect of gut microbiota on gene expression in trunk and hindlimb muscles. Expression pattern analysis showed that PCGs were predominantly downregulated in GF pigs (head: 60.5%; forelimb: 55.3%; trunk: 43.2%; hindlimb: 70.4%), whereas LncRNA and TUCP exhibited complex bidirectional regulation. Tissue specificity analysis (Figure 4D) revealed that 62.61% of differentially expressed PCGs (422/674), 70.02% of LncRNAs (1,210/1,728), and 66.84% of TUCPs (129/193) were differentially expressed in only one tissue, with only one LncRNA differentially expressed across all 14 tissues, indicating significant tissue heterogeneity in the transcriptional response to gut microbiota and distinct gene expression regulation patterns across muscle sites.

**Figure 5:**
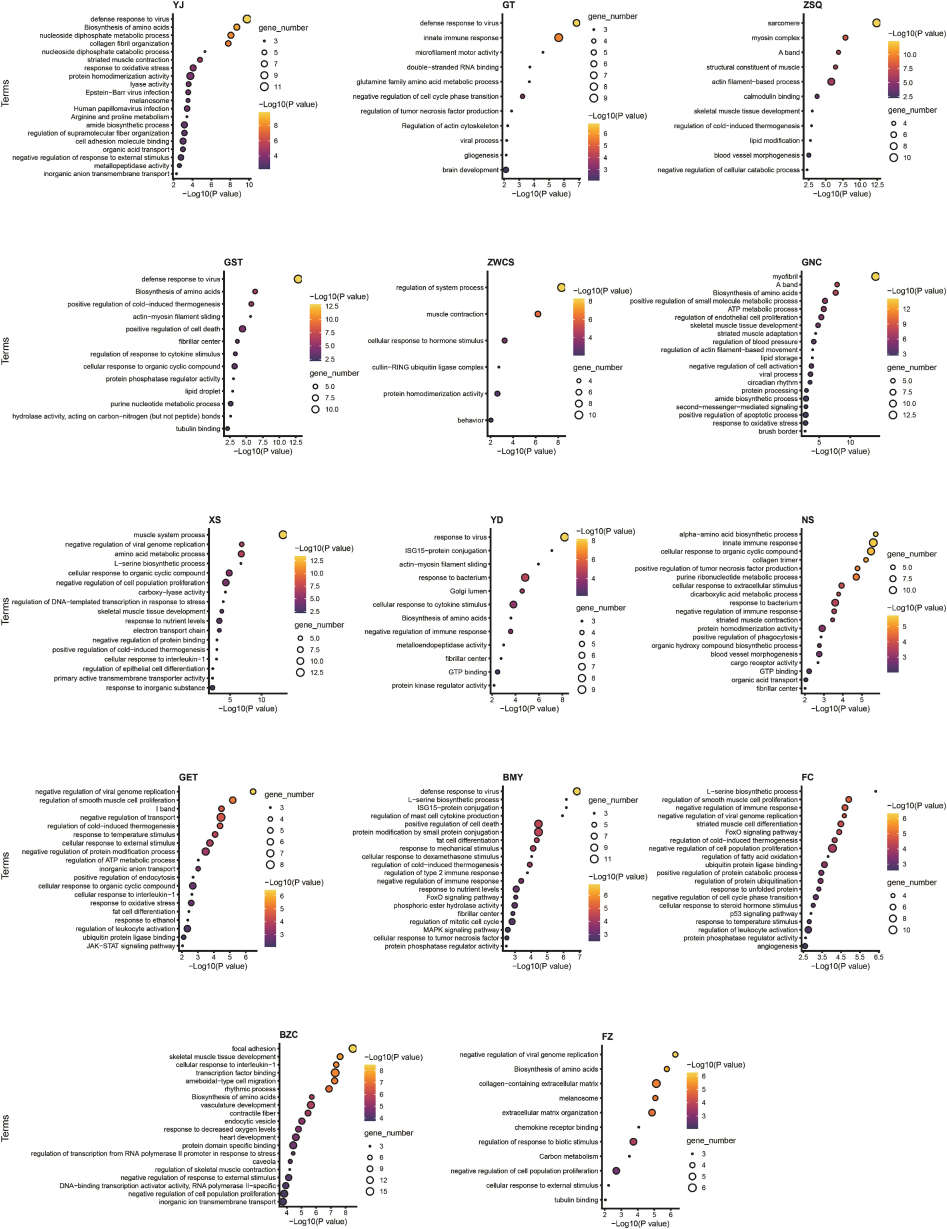
Pathway enrichment analysis of differentially expressed genes in individual muscle tissues of GF and SPF pigs. YJ (MAS, masseter), GT (BH, brachiocephalicus), ZSQ (FDP, flexor digitorum profundus), GST (TB, triceps brachii), ZWCS (EDL, extensor digitorum lateralis), GNC (MF, medial femoral), XS (PP, pectoralis profundus), YD (PM, psoas major), NS (ADD, adductores), GET (BF, biceps femoris), BMY (SOL, soleus), FC (GAS, gastrocnemius), BZC (LDM, longissimus dorsi), FZ (RA, rectus abdominis).

Functional enrichment analysis (Supplementary Figure 4E) revealed that differentially expressed genes in muscle tissues between GF and SPF pigs were primarily enriched in muscle-related functions, including muscle development (GO:0061061, *p* = 2.19 × 10^−24^), contractile fiber (GO:0043292, *p* = 7.14 × 10^−26^), and striated muscle contraction (GO:0006941, *p* = 3.36 × 10^−12^), as well as metabolic pathways such as amino acid biosynthesis (hsa01230, *p* = 2.02 × 10^−9^), pyruvate metabolism (GO:0006090, *p* = 7.49 × 10^−10^), and immune-related processes like defense response to virus (GO:0051607, *p* = 1.38 × 10^−13^). Further analysis showed that four pathways—amino acid biosynthesis, defense response to virus, muscle development, and contractile fiber—were consistently enriched across at least 10 of the 14 muscle tissues, whereas five pathways, including DNA-binding transcription factor binding (GO:0140297) and pyruvate metabolism, exhibited tissue specificity, being enriched in at most three tissues. Pathway analysis of individual muscle tissues (Figure 5) confirmed significant differences in functional enrichment across muscle sites, indicating that the impact of gut microbiota absence on muscle tissues is tissue-specific.

Muscle fiber type and metabolic pathway analysis revealed significant differences between GF and SPF pigs across multiple muscle tissues. In the flexor digitorum profundus, pectoralis profundus, and medial femoral muscle, differentially expressed genes (e.g., *ATP2A2*, *MYH7*) were significantly enriched in the transition between fast and slow muscle fibers pathway (GO:0014883, *p* < 1 × 10^−10^), consistent with the reduced proportion of type I muscle fibers in GF pigs. Metabolic analysis indicated that differentially expressed genes in the masseter muscle, pectoralis profundus, and medial femoral muscle (e.g., *ALDOA*, *PCK2*) were enriched in pyruvate metabolism (GO:0006090, *p* < 0.0001), those in the pectoralis profundus (e.g., *FASN*, *PPARGC1A*) were enriched in fatty acid metabolism (GO:0006631, *p* = 0.005), and genes across 11 muscle tissues (e.g., *ARG2*, *PHGDH*) were commonly enriched in the amino acid biosynthesis pathway (hsa01230). Notably, differentially expressed genes in four muscle tissues, including the triceps brachii (e.g., *PPARGC1A*, *PRKAG2*), were significantly enriched in the AMPK signaling pathway (hsa04152, *p* < 0.01). These findings align with changes in metabolite levels in the corresponding muscle tissues, confirming that the impact of gut microbiota absence on skeletal muscle is markedly tissue-specific.

### Gut microbiota modulates myofiber typing through MYH isoform-specific expression

**Figure 6:**
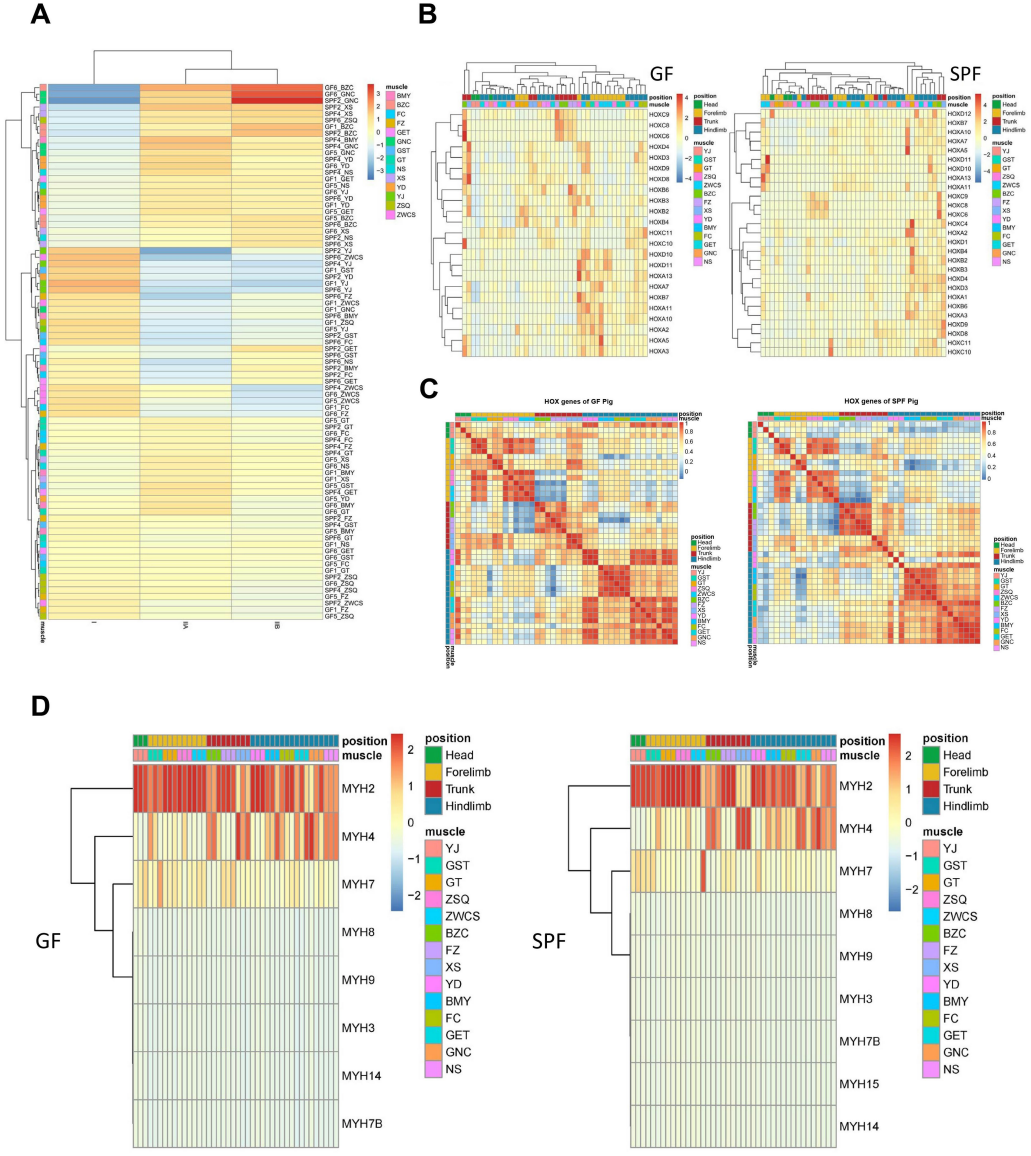
Analysis of muscle fiber types and key gene family expression profiles in muscle tissues of GF and SPF pigs. **(A)** Expression proportions of type I, IIA, and IIB muscle fibers in muscle tissues of GF and SPF pigs. **(B)** Expression patterns of HOX family genes in muscle tissues of GF and SPF pigs. **(C)** Correlation of HOX family gene expression patterns in muscle tissues of GF and SPF pigs. **(D)** Expression patterns of MYH isoforms in different muscle tissues of GF and SPF pigs.

Based on preliminary findings suggesting that gut microbiota alterations may influence muscle fiber typing, this study integrated transcriptome data with unpublished single-nucleus sequencing data from our group to identify three main myonuclei types (type I, IIA, and IIB) using marker genes, followed by deconvolution analysis of Bulk RNA-seq data using their gene expression matrices to calculate the relative proportions of these myonuclei types (Figure 6A). The results showed that, compared to SPF pigs, GF pigs exhibited a lower proportion of type I myonuclei in the forelimb extensor digitorum lateralis, whereas SPF pigs had higher proportions of type IIA and IIB myonuclei in the pectoralis profundus and type I myonuclei in the hindlimb gastrocnemius muscle. Further analysis of genes related to skeletal muscle development and fiber typing revealed significant differences in their expression between GF and SPF pig skeletal muscle.

Through literature review and differential gene pathway enrichment analysis, this study focused on three gene categories critical for skeletal muscle formation and function: *HOX* superfamily homeotic transcription factors, *HOX* family gene sets, myogenic factors, and myosin heavy chain (*MYH*) isoforms. The *HOX* superfamily, a core regulator of animal morphogenesis and development, exhibited significant expression differences for its 35 member genes between GF pigs (23 expressed) and SPF pigs (27 expressed) (Figure 6B). Correlation analysis revealed that *HOX* gene expression in GF pig muscles lacked anatomical site specificity, whereas the expression pattern in the masseter muscle of SPF pig heads was distinctly separated from other sites (Figure 6C). Further analysis of 129 *Homeobox* family genes showed expression differences between GF pigs (59 expressed) and SPF pigs (69 expressed) (Supplementary Figure 5A), with the *Homeobox* gene expression pattern in the masseter muscle of SPF pig heads exhibiting site specificity, while no significant differences were observed among GF pig muscle sites (Supplementary Figure 5B). Key regulatory factors, such as *MEOX2*, *PRRX1*, and *LHX2*, were highly expressed in the masseter muscle of SPF pig heads but downregulated in GF pigs, suggesting that gut microbiota absence may lead to a loss of tissue localization specificity in *HOX* family genes, thereby affecting the establishment of the skeletal muscle developmental regulatory network.

Skeletal muscle contributes to systemic physiological regulation by secreting myokines, a class of metabolic regulatory hormones that promote muscle fiber growth. Analysis revealed that 87.38% of myokines (485/555) in GF pig muscle tissues showed transcriptional evidence in at least one site, with 36.22% (201/551) being ubiquitously transcribed, whereas in SPF pigs, this proportion reached 97.66% (542/555), indicating that gut microbiota absence significantly suppresses myokine transcriptional activity in GF pigs (Supplementary Figure 5C). Both GF and SPF pig muscle tissues expressed 222 core myokines at high abundance (TPM >10), associated with fundamental functions such as angiogenesis (*VEGF*), NAD biosynthesis (*NAMPT*), and inflammatory responses (*AIMP1*, *CMTM6*, *CXCL12*, *HMGB1*). Only a small number of myokines (11 in GF pigs, 18 in SPF pigs) showed abundance differences between the two groups, suggesting that myokine expression profiles across different skeletal muscle sites are highly conserved.

The heterogeneity of mammalian skeletal muscle is primarily reflected in muscle fiber types with distinct contractile and metabolic characteristics, which can be distinguished by the expression patterns of myosin heavy chain (*MYH*) isoforms. Clustering analysis revealed that GF pig muscle tissues co-expressed eight *MYH* isoforms (predominantly *MYH2*), whereas SPF pigs expressed nine isoforms (also predominantly *MYH2*), with *MYH15* being specifically expressed in SPF pigs (Figure 6D). Significant differences were observed in the expression patterns of *MYH2* (type IIa fast oxidative fibers), *MYH4* (type IIb fast glycolytic fibers), and *MYH7* (type I slow oxidative fibers) across different muscle tissues in GF pigs, indicating that gut microbiota absence not only alters the overall *MYH* isoform expression profile but may also disrupt normal skeletal muscle development and functional differentiation in GF pigs by modifying the expression patterns of specific fiber type-related isoforms.

### Co-expression networks and LncRNA-mediated regulation underlie gut microbiota-muscle interactions

**Figure 7:**
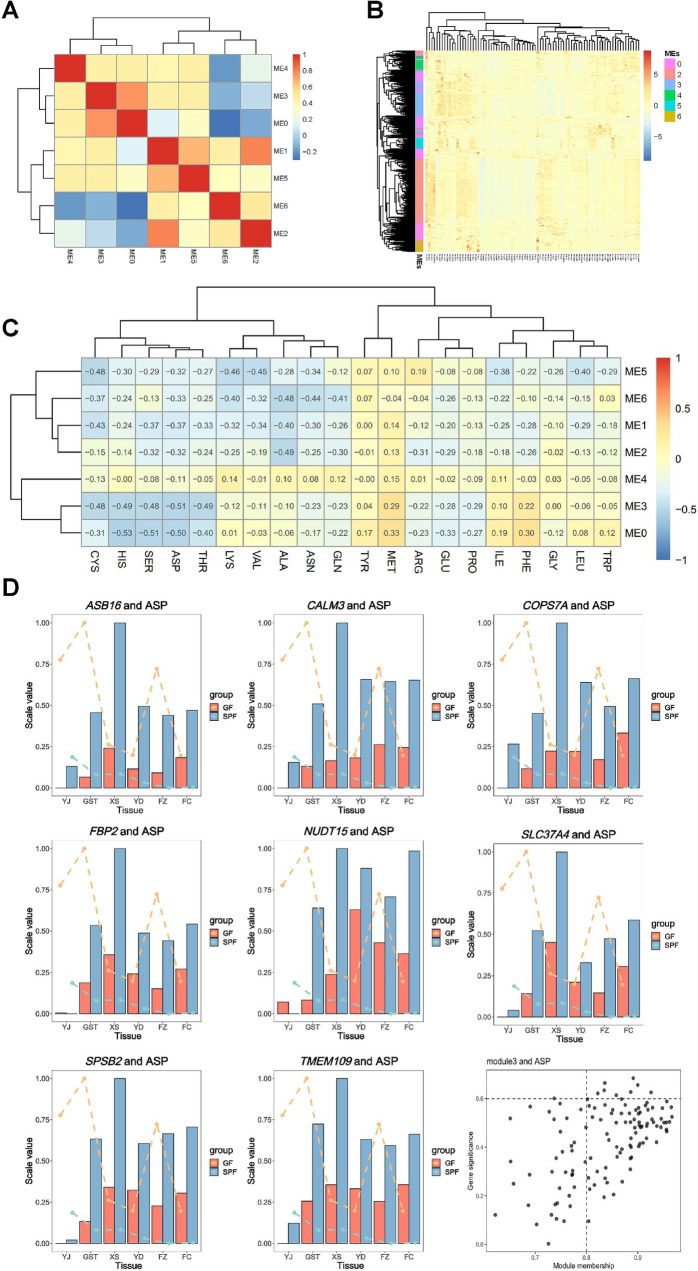
Correlation analysis of co-expression network modules with amino acid metabolism in muscle tissues of GF and SPF pigs. **(A)** Correlations between modules. **(B)** Expression profiles of genes within modules. **(C)** Correlations between modules and amino acid content. **(D)** Importance of aspartic acid (ASP)-related genes and distribution of ME3 module members. Bar graphs represent gene expression levels (normalized proportional values), lines indicate phenotype values (normalized proportional values), and black dots denote genes. YJ (MAS, masseter muscle), GST (TB, triceps brachii), XS (PP, pectoralis profundus), YD (PM, psoas major muscle), FZ (RA, rectus abdominis), FC (GAS, gastrocnemius muscle).

Integrated metabolomic and transcriptomic analysis revealed that GF and SPF pigs exhibited changes in amino acid, fatty acid, short-chain fatty acid, organic acid, and free fatty acid compositions in muscle tissues from the head (masseter muscle), forelimb (triceps brachii), trunk (pectoralis profundus, rectus abdominis), and hindlimb (psoas major muscle, gastrocnemius muscle). WGCNA (weighted gene co-expression network analysis) analysis clustered gene expression data from 84 samples into seven co-expression modules (modules 0–6), containing 232, 7,229, 243, 120, 52, 38, and 36 genes, respectively. Inter-module correlation analysis (Figure 7A) indicated high correlations among ME0, ME3, and ME4, while ME1, ME2, ME5, and ME6 formed another highly correlated cluster. These findings reveal systemic changes in metabolite composition and gene co-expression patterns across muscle tissues from different anatomical sites under conditions of gut microbiota absence.

Figure 7B shows that genes within the same module are generally clustered together, indicating that the previously identified modules are reliable. WGCNA functional module analysis (Supplementary Figure 6) revealed that module 0 was significantly enriched in pathways related to muscle organ development, including muscle organ development (GO:0007517, *p* = 1.45 × 10^−8^), contractile fiber (GO:0043292, *p* = 1.85 × 10^−8^), and actin-myosin filament sliding (GO:0033275, *p* = 1.24 × 10^−5^). Additionally, this module was involved in lipid metabolism regulation, such as cellular response to lipids (GO:0071396, *p* = 3.11 × 10^−6^) and regulation of fatty acid oxidation (GO:0046320, *p* = 2.92 × 10^−8^). Furthermore, genes in module 0 were significantly enriched in pathways related to regulation of cell death (GO:0010942, *p* = 4.98 × 10^−8^) and response to bacteria (GO:0009617, *p* = 4.12 × 10^−5^), suggesting that this gene co-expression network plays multiple biological roles in muscle development and metabolic regulation.

WGCNA functional module analysis revealed the specific functions of each module in muscle tissues (Supplementary Figure 6). Module 1 (top 300 genes) was significantly enriched in epigenetic regulation pathways, including chromatin organization (GO:0006325, *p* = 2.19 × 10^−16^), regulation of DNA metabolism (GO:0051052, *p* = 1.78 × 10^−12^), and histone modification (GO:0016570, *p* = 2.78 × 10^−12^). Module 2 was primarily involved in mitochondrial function (GO:0005743, *p* = 1.89 × 10^−100^) and energy metabolism processes, such as cardiac muscle contraction (hsa04260, *p* = 2.91 × 10^−26^) and fatty acid metabolism (GO:0006631, *p* = 5.64 × 10^−17^). Module 3 was enriched in carbohydrate metabolism (GO:0005975, *p* = 8.66 × 10^−21^) and regulation of muscle contraction (GO:0006937, *p* = 6.03 × 10^−9^), and was also associated with AMPK (hsa04152, *p* = 5.04 × 10^−5^) and FoxO (hsa04068, *p* = 7.36 × 10^−5^) signaling pathways. Module 4 was involved in adipocyte differentiation (GO:0045444, *p* = 2.72 × 10^−8^) and regulation of inflammation (GO:0050727, *p* = 1.94 × 10^−7^). Module 5 was primarily associated with lipid metabolism regulation, including the PPAR signaling pathway (hsa03320, *p* = 2.79 × 10^−12^) and insulin signaling pathway (hsa04910, *p* = 1.91 × 10^−5^). Module 6 was enriched in extracellular matrix organization (GO:0031012, *p* = 1.89 × 10^−12^) and regulation of chemotaxis (GO:0050920, *p* = 6.72 × 10^−5^).

Gene-trait association analysis (Figure 7C) revealed significant correlations between 20 amino acid traits and all seven modules, confirming that gut microbiota broadly influence muscle amino acid metabolism. Notably, aspartic acid (ASP) exhibited the strongest negative correlation with ME3. By setting thresholds (|MM| >0.8 and |GS| >0.6), eight hub genes (*CALM3*, *TMEM109*, *SLC37A4*, *SPSB2*, *COPS7A*, *NUDT15*, *FBP2*, and *ASB16*) were identified (Figure 7D), which likely play critical roles in mediating gut microbiota effects on muscle metabolism regulation.

Correlation analysis of short-chain fatty acids with gene modules (Supplementary Figure 7A) revealed significant associations between four out of five short-chain fatty acid traits and all seven modules. Notably, acetate exhibited a strong negative correlation with the ME3 module (correlation coefficient <−0.8). By applying stringent criteria (|MM| >0.8 and |GS| >0.5), three hub regulatory genes were identified: *TNNI2* (troponin I2), *HOMER1* (postsynaptic scaffold protein), and *DNAI1* (dynein intermediate chain 1) (Supplementary Figure 7B). Correlation analysis of free fatty acids with gene modules (Supplementary Figure 7C) showed significant associations between all 34 free fatty acid traits and the seven modules. Notably, FA 17:1 exhibited a strong positive correlation with the ME2 module (correlation coefficient >0.8). By applying stringent criteria (|MM| >0.8 and|GS| >0.6), four hub regulatory genes were identified: *PODN* (podocan, extracellular matrix protein), *MRPL9* (mitochondrial ribosomal protein), *AMN1* (negative regulator of meiosis), and *SNRPN* (small nuclear ribonucleoprotein) (Supplementary Figure 7D).

Correlation analysis of organic acids with gene modules (Supplementary Figure 8A) revealed significant associations between 11 out of 14 organic acid traits and all seven modules. Notably, lactate levels exhibited a strong positive correlation with the ME0 module (correlation coefficient >0.8). By applying screening criteria (|MM| >0.8 and|GS| >0.2), a key regulatory gene, *SERPINE1* plasminogen activator inhibitor (1), was identified (Supplementary Figure 8B). Correlation analysis of fatty acids with gene modules (Supplementary Figure 8C) showed significant associations between all 31 fatty acid traits and the seven modules. Notably, the saturated fatty acid C17:0 exhibited a strong positive correlation with the ME2 module (correlation coefficient >0.8), leading to the identification of seven hub genes: *HSDL2* hydroxysteroid dehydrogenase-like protein (2), *MGST3* (microsomal glutathione S-transferase) (3), *MRPL9* (mitochondrial ribosomal protein L9), *PRKAG1* (AMP-activated protein kinase gamma 1 subunit), *ACADVL* (very long-chain acyl-CoA dehydrogenase), *GSTK1* (glutathione S-transferase kappa 1), and *SNRPN* (small nuclear ribonucleoprotein) (Supplementary Figure 8D). These genes are primarily involved in lipid metabolism (*HSDL2*, *ACADVL*), oxidative stress response (*MGST3*, *GSTK1*), and energy sensing (*PRKAG1*). Notably, the polyunsaturated fatty acid C18:3n6 exhibited a strong negative correlation with the ME0 module (correlation coefficient <−0.8), leading to the identification of a key regulatory gene, *SERPINE1* (plasminogen activator inhibitor 1) (Supplementary Figure 8E).

Correlation analysis of muscle fiber phenotypes with gene modules (Supplementary Figure 9A) revealed significant associations between all five muscle fiber trait indicators and the seven modules. Notably, type I muscle fiber diameter exhibited a strong positive correlation with the ME5 module (correlation coefficient >0.8), leading to the identification of three hub regulatory genes: *CEBPA* (CCAAT/enhancer-binding protein alpha), *LIPE* (hormone-sensitive lipase), and *PC* (pyruvate carboxylase) (Supplementary Figure 9B). These genes are involved in biological processes including adipocyte differentiation (*CEBPA*), lipolysis (*LIPE*), and energy metabolism (*PC*). Additionally, type I muscle fiber diameter showed a strong negative correlation with the ME4 module (correlation coefficient <−0.8), leading to the identification of a key regulatory gene, *IFRD1* (interferon-related developmental regulator 1) (Supplementary Figure 9C). This gene, a regulator of muscle growth and differentiation, may directly influence the composition of muscle fiber types through its expression changes. These findings reveal that gut microbiota influence the morphological characteristics specific to muscle fiber types by regulating the expression of genes associated with lipid metabolism (*CEBPA*, *LIPE*), energy metabolism (*PC*), and muscle development (*IFRD1*).

Deep correlation analysis of muscle fiber phenotypes with gene modules (Supplementary Figure 10) revealed a strong positive correlation between type II muscle fiber diameter and the ME5 module (correlation coefficient >0.8), leading to the identification of nine hub regulatory genes: *ME1* (malic enzyme 1), *ADIPOQ* (adiponectin), *CEBPA*, *LIPE*, *PC*, *ALDH1L1* (aldehyde dehydrogenase 1 family member L1), *PLIN1* (perilipin 1), *RDH16* (retinol dehydrogenase 16), and *FASN* (fatty acid synthase). These genes form a functional network encompassing key pathways such as lipid metabolism (*FASN*, *PLIN1*), energy metabolism (*ME1*, *PC*), and adipokine signaling (*ADIPOQ*) (Supplementary Figure 10).

Further analysis (Supplementary Figure 11A) revealed that the positive correlation between total muscle fiber diameter and the ME5 module (correlation coefficient >0.8) was primarily driven by the *PC* gene. Detailed correlation analysis of muscle fiber characteristics with gene modules (Supplementary Figure 11B) revealed a strong positive correlation between the proportion of type I muscle fiber area and the ME6 module (correlation coefficient >0.8), leading to the identification of five hub regulatory genes: *ENSSSCG00000034949* (function unannotated), *COL12A1* (collagen type XII alpha 1 chain), *DKK3* (Dickkopf-related protein 3), *STEAP1* (six-transmembrane epithelial antigen 1), and *SEMA3D* (semaphorin 3D). These genes are involved in key biological processes, including extracellular matrix remodeling (*COL12A1*), Wnt signaling regulation (*DKK3*), and axon guidance (*SEMA3D*).

Additionally, the proportion of type I muscle fiber area showed a strong negative correlation with the ME3 module (correlation coefficient <−0.8), leading to the identification of seven key regulatory genes: *ENSSSCG00000004497*, *AC002996.1* (function unannotated), *PGM2L1* (phosphoglucomutase 2-like 1), *PHKA1* (phosphorylase kinase alpha 1), *ENHO* (energy homeostasis-related hormone), *DNAI1* (dynein axonemal intermediate chain 1), and *ENSSSCG00000018003* (Supplementary Figure 12A). Precise correlation analysis of muscle fiber composition with gene modules (Supplementary Figure 12B) revealed a strong positive correlation between the proportion of type I muscle fiber number and the ME6 module (correlation coefficient >0.8), with the core regulatory gene identified as *ENSSSCG00000034949* (function not yet characterized). This gene may specifically influence the numerical distribution of type I muscle fibers through unknown molecular mechanisms. Additionally, the proportion of type I muscle fiber number exhibited a strong negative correlation with the ME3 module (correlation coefficient <−0.8), with key regulatory genes including *ENHO* (energy homeostasis-related hormone), *DNAI1* (dynein axonemal intermediate chain 1), and *ENSSSCG00000018003* (function unannotated) (Supplementary Figure 12C).

Through integrated multi-omics correlation analysis of muscle nutrients and muscle fiber phenotypes, we identified 25 core regulatory genes, including 16 genes closely associated with muscle nutrient metabolism (e.g., *CALM3*, *SERPINE1*) and nine key genes regulating muscle fiber characteristics (e.g., *IFRD1*, *ENHO*), with *DNAI1* identified in both phenotype analyses, suggesting its central role in muscle function regulation. Additionally, we identified 40 differentially expressed LncRNAs and their 29 target protein-coding genes (PCGs) (Supplementary Table 10), which collectively form a multilayered regulatory network of the gut microbiota-muscle axis.

Through stringent neighborhood correlation analysis (5 kb upstream of TSS to 1 kb downstream of TTS), we identified LncRNA-PCG functional pairs regulated by gut microbiota absence (Supplementary Tables 11, 12). The analysis revealed that the masseter muscle exhibited the highest number of seven co-expressed regulatory units, whereas the gastrocnemius muscle showed only one pair. Notably, all identified LncRNA-PCG pairs displayed a coordinated regulatory pattern (synchronous upregulation or downregulation, FDR-BH <0.05), and this highly consistent co-expression feature (TPM >1) strongly suggests that these adjacent LncRNAs may regulate the expression of nearby protein-coding genes (PCGs) through cis-acting mechanisms, thereby mediating the specific transcriptional regulation of muscle tissues in response to gut microbiota absence. This conserved co-regulatory pattern provides critical insights into the functional mechanisms of LncRNAs within the gut microbiota-muscle axis.

**Figure 8:**
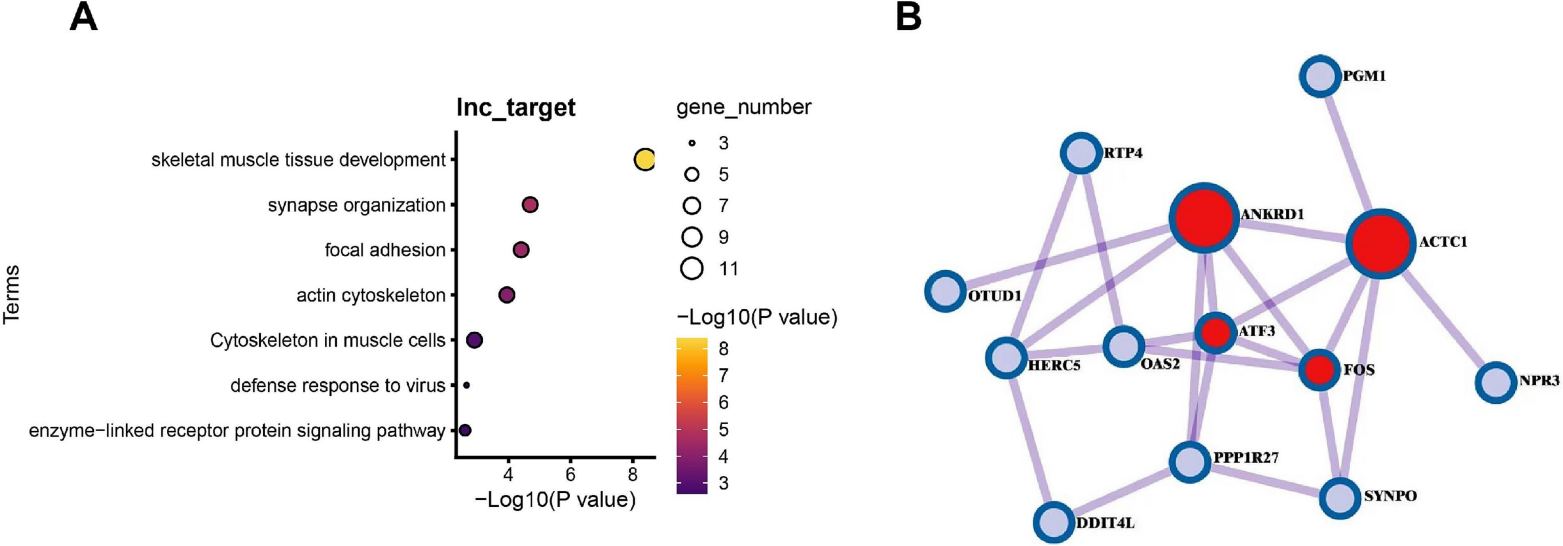
Analysis of LncRNA-target gene regulatory networks and the impact of gut microbiota absence in muscle tissues of GF and SPF pigs. **(A)** Functional enrichment analysis of target genes of differentially expressed LncRNAs in muscle tissues of GF and SPF pigs. **(B)** Pathway analysis of differential regulation of PCG-LncRNA pairs due to gut microbiota absence in muscle tissues of SPF and GF pigs.

Functional enrichment analysis of differentially expressed LncRNA target genes (Figure 8A) revealed that 29 target genes were significantly enriched in pathways related to muscle development and function, including muscle organ development (GO:0007517, *p* = 8.10 × 10^−7^) and sarcomere I band structure (GO:0031674, *p* = 7.74 × 10^−5^), among other muscle-specific processes. Additionally, these genes were involved in viral defense responses (GO:0051607, *p* = 4.33 × 10^−4^) and postsynaptic signal transduction (GO:0014069, *p* = 1.01 × 10^−3^), particularly mediating intercellular communication through enzyme-linked receptor protein signaling pathways (GO:0007167, *p* = 5.74 × 10^−3^). These findings suggest that gut microbiota may influence multiple biological processes in muscle tissues, including development, contractile function, and immune defense, through LncRNA regulatory networks.

Functional network analysis (Figure 8B) revealed that differentially expressed protein-coding genes (PCGs) and LncRNAs form key regulatory modules that directly influence skeletal muscle development and metabolism. Specifically, cardiac actin alpha 1 (*ACTC1*) contributes to myofibril assembly, phosphoglucomutase-1 (*PGM1*) regulates the glycolysis pathway, while transcription factors *FOS* and *ATF3* integrate stress responses and proliferative signals, collectively forming a multilayered regulatory network. The pairing relationships between these PCGs and adjacent LncRNAs (e.g., *ACTC1-ENSSSCG00000012345*) suggest that LncRNAs may precisely modulate muscle-specific gene expression patterns through cis-regulatory mechanisms, thereby mediating the systemic effects of gut microbiota on muscle development (actin assembly), energy metabolism (glycolysis regulation), and stress adaptation (transcriptional reprogramming). This coordinated LncRNA-PCG regulatory pattern offers novel insights into the molecular mechanisms underlying gut microbiota-muscle interactions. Correlation analysis of differentially expressed LncRNAs and PCGs between two pig groups revealed that approximately 50% of LncRNA-PCG pairs exhibited high correlation (|Cor| >0.8). Whether these PCGs are regulated by LncRNAs through cis- or trans-acting mechanisms requires further validation.

## Discussion

This study systematically compared phenotypical parameters, nutrient composition and transcriptomic profiles of skeletal muscles between GF and SPF pigs to investigate the impact of gut microbiota on porcine skeletal muscle development and functional metabolism. Results revealed that microbiota deficiency significantly affected muscle development, fiber-type distribution and nutrient metabolism, with region-specific patterns across different muscle groups.

Accounting for approximately 50% of body weight, skeletal muscle serves as a crucial metabolic organ (Grosicki et al., 2018) with functions including thermogenesis and glucose regulation (Lieber et al., 2017). Its mass is regulated by inflammatory status (Beyer et al., 2012), hormonal changes (Sakuma and Yamaguchi, 2012) and gut physiology (Azzolino et al., 2019). Evidence indicates gut microbiota enhances skeletal muscle growth and quality (Yan et al., 2016)—*Bacteroides thetaiotaomicron* transplantation increased muscle mass while reducing fat in chow-fed mice and improved muscle mass in high-fat diet mice (Liu et al., 2017), while *Lactobacillus plantarum TWK10* supplementation promoted muscle hypertrophy and exercise performance (Chen et al., 2016).

In our study, GF pigs showed smaller myofiber diameters in multiple muscles compared to SPF pigs, with statistically significant difference in extensor digitorum lateralis (*p* < 0.05), confirming the promotive role of microbiota in muscle development. These findings align with reports that GF mice had lower gastrocnemius muscle weight than SPF controls (Hsu et al., 2015), and that probiotics LC122 and BL986 increased muscle mass in aged mice (Ni et al., 2019). Notably, microbiota reconstitution increased myofiber cross-sectional area and enhanced oxidative metabolism with upregulated expression of Rapsyn and Lrp4 (Lahiri et al., 2019), while short-chain fatty acids reversed muscle injury and branched-chain amino acid metabolism regulated development (Waddell et al., 2008). The higher leucine levels in GF pigs’ muscles than SPF pigs further indicated microbiota depletion affects metabolism and muscle development.

The early postnatal period represents a critical window for myofiber metabolic and contractile type transitions, during which fiber-type distributions show minimal interbreed variation. Neonatal animals predominantly exhibit oxidative fiber types, with age-dependent gradual decline in oxidative fibers and rapid glycolytic fiber expansion until reaching a stable equilibrium in adulthood (Lefaucheur et al., 2001).

Gut microbiota has been demonstrated to modulate fiber-type switching through multiple mechanisms. Yan et al. (2016) reported that obese pig microbiota transplantation upregulated *MYH7* (slow myosin) while downregulating *MYH4* (fast myosin) in GF mice, promoting slow-twitch fiber formation. This aligns with findings that *Lactobacillus plantarum* supplementation improved exercise capacity concomitant with increased type I fibers in gastrocnemius (Chen et al., 2016). Such microbiota-induced fiber-type shifts may involve mitochondrial biogenesis (Kootte et al., 2012), with transmissible characteristics including obesity-associated phenotypes (Yan et al., 2016; Nicholson et al., 2012).

In our GF model, reduced type I fiber proportion and cross-sectional area were observed in multiple muscles (flexor digitorum profundus, extensor digitorum lateralis, pectoralis profundus, biceps femoris, gastrocnemius and vastus medialis), showing predominant hindlimb localization with partial forelimb and trunk involvement. Transcriptomics revealed significant enrichment (GO:0014883) for slow/fast fiber transition pathways (*p* = 7.62 × 10^−12^ to 4.47 × 10^−10^) in three affected muscles, with putative regulatory roles for calcium handling (*ATP2A2*) and myofilament components (*MYH7*, *TNNC1*, *TNNI1*, *TNNT1*), suggesting microbiota orchestrates fiber-type specification through excitation-contraction coupling modulation.

Gut microbiota synthesizes bioavailable amino acids for host utilization (Metges, 2000). GF mice exhibited altered muscle amino acid metabolism with elevated glycine [muscle energy substrate (Meléndez-Hevia et al., 2009) and mitochondrial stress responder (Quirós et al., 2017)] and alanine levels (Lahiri et al., 2019). BCAA deficiency accelerated catabolism (Lahiri et al., 2019), and BCAA transamination yielded alanine accumulation concomitant with increased alanine aminotransferase (ALT) expression in GF muscle, suggesting proteolytic adaptation to microbiota deprivation (Blanton et al., 2016). Our GF pigs showed higher histidine, alanine and other amino acids in six muscles versus SPF controls, with 11/14 muscles displaying differentially expressed gene (DEG) enrichment in amino acid biosynthesis pathways (KEGG hsa01230).

Regarding lipid metabolism, GF mice had enhanced fatty acid oxidation capacity (Bäckhed et al., 2007), whereas microbiota transplantation remodeled muscular lipid metabolism and deposition (Yan et al., 2016). Mechanistically, microbiota presence downregulated AMPK expression to suppress β-oxidation (Bäckhed et al., 2007), and obese pig microbiota transplantation upregulated acetyl-CoA carboxylase (*ACC*) and lipoprotein lipase (*LPL*) while downregulating carnitine palmitoyltransferase 1B (*CPT1B*), promoting lipogenesis over oxidation (Yan et al., 2016; Goldberg et al., 2009). Conversely, GF mice showed enhanced AMPK phosphorylation to potentiate anti-obesity fatty acid oxidation (Lahiri et al., 2019; Bäckhed et al., 2007), indicating microbiota’s bidirectional regulation of muscle energy metabolism.

Skeletal muscle development is regulated by genetic and nutritional factors, with gene expression playing a pivotal role. Leveraging RNA sequencing (RNA-seq) technology for its high sensitivity and broad coverage (Hrdlickova et al., 2017), we analyzed transcriptomes from 14 muscle tissues of GF and SPF pigs to elucidate molecular mechanisms underlying gut microbiota depletion. Significant differential expression of protein-coding genes (PCGs) between GF and SPF pigs was enriched in muscle structure development (GO:0061061, *p* = 2.19 × 10^−24^) and skeletal muscle tissue development (GO:0007519, *p* = 3.30 × 10^−18^), correlating with reduced fiber diameter in muscles such as the flexor digitorum profundus.

Dysregulated expression of *MSTN*, a TGF-β family member that negatively regulates myogenesis (Esposito et al., 2022), suppressed *MyoG* and *MyoD* via the MEK1/2 signaling pathway (Yang et al., 2006). These findings align with observations in GF mice showing reduced *MyoG* and *MyoD* expression compared to SPF controls (Lahiri et al., 2019), alongside perturbations in *POPDC2*, a gene essential for skeletal muscle development (Kirchmaier et al., 2012). Long non-coding RNAs (LncRNAs), critical regulators of myogenesis, exhibited microbiota-dependent expression patterns in GF pigs. Their cis-acting target genes, including *ATF3* and *ANKRD1*, were enriched in muscle organ development (GO:0007517, *p* = 8.10 × 10^−7^) and skeletal muscle tissue development (GO:0007519, *p* = 1.08 × 10^−6^). *ANKRD1* further modulates muscle stress responses (Nakada et al., 2003) and inflammatory signaling in *C2C12* myotubes (Liu et al., 2015).

Muscle-specific responses to microbiota depletion were evident: the flexor digitorum profundus showed enrichment in slow/fast fiber-type switching pathways (GO:0014883, *p* < 10^−10^), driven by *ATP2A2* and *MYH7* (Yan et al., 2016; Chen et al., 2016). Additionally, 11 muscles displayed amino acid biosynthesis alterations (KEGG hsa01230), while the pectoralis profundus exhibited perturbed fatty acid metabolism (GO:0006631, *p* = 5.00 × 10^−3^) and AMPK signaling (hsa04152). The masseter muscle demonstrated pyruvate metabolism dysregulation (GO:0006090), underscoring the microbiota’s diverse regulatory roles across muscle types.

Nevertheless, this study has several limitations that should be acknowledged. First, the restricted sample size due to the specialized rearing conditions of GF pigs may compromise statistical power. Second, while our focus was on transcriptomic profiling, future studies should incorporate proteomic and metabolomic approaches to validate and extend these findings. Additionally, although our previous study provided a comprehensive analysis of the gut metagenome in SPF pigs (Wen et al., 2024), future research should integrate metagenomics to further elucidate the regulatory mechanisms of specific microbial taxa and their metabolic products (e.g., SCFAs) on skeletal muscle development.

In summary, our multi-omics analysis demonstrates the profound impact of gut microbiota on porcine skeletal muscle development and functional metabolism. These findings advance the gut-muscle axis paradigm and provide a theoretical foundation for microbiota-targeted strategies to enhance muscle health and meat quality. Future research should delineate the precise molecular mechanisms underlying microbiota-muscle crosstalk to enable breakthroughs in both animal husbandry and human therapeutics.

## Conclusion

This study systematically compared skeletal muscle phenotypes, nutrient profiles, and transcriptomic data between GF and SPF pigs to comprehensively elucidate the critical role of gut microbiota in porcine skeletal muscle development and functional metabolism. Key findings demonstrated that microbiota depletion significantly impaired skeletal muscle development in GF pigs, manifesting as reduced myofiber diameter and altered type I fiber composition, with particularly prominent effects in forelimb extensor digitorum lateralis. Nutrient analysis revealed decreased short-chain fatty acid (SCFA) content in GF muscles alongside elevated amino acid and organic acid levels, while free fatty acid profiles showed muscle-specific patterns, demonstrating microbiota’s pivotal role in muscle metabolic regulation.

Transcriptomics revealed generally lower expression of protein-coding genes (PCGs), long non-coding RNAs (LncRNAs) and transcripts of uncertain coding potential (TUCPs) in GF muscles, with differentially expressed genes enriched in muscle development and immune-related pathways. Altered expression patterns of HOX genes, homeobox genes, myokines and myosin heavy chain (MYH) isoforms confirmed microbiota’s regulatory effects on myogenesis and fiber-type specification. Weighted gene co-expression network analysis (WGCNA) identified hub genes strongly correlated with muscle nutrient profiles and fiber phenotypes, providing potential targets for future investigations. Cis-target prediction of differential LncRNAs indicated their predominant involvement in skeletal muscle development and immune responses, suggesting LncRNA-mediated regulation of the gut-muscle axis via neighboring genes.

This study provides novel evidence for understanding gut-muscle axis mechanisms and establishes a theoretical foundation for improving muscle health and meat quality via microbiota modulation. Future studies should dissect the molecular mechanisms underlying microbiota-muscle crosstalk to advance applications in animal husbandry and human health.

## Data Availability

The datasets presented in this study can be found in the Genome Sequence Archive (GSA), accession number CRA025824.
