# Supplementary material for: Gut microbiota orchestrates skeletal muscle development and metabolism in germ-free and SPF pigs
Source: Front Microbiol. 2025 Jun 16;16:1615884. doi: 10.3389/fmicb.2025.1615884 (PMC12206747; doi:10.3389/fmicb.2025.1615884)
Supplement: Supplementary file 1 [file Supplementary_file_1.docx]

**Supplementary Table 1. Comparative morphological analysis of muscle tissues between SPF pigs and GF pigs.**

| **Sample ID** | **Type I muscle fiber diameter (μm)** | **Type II muscle fiber diameter (μm)** | **Composite (Type I+II) muscle fiber diameter (μm)** | **Type I fiber area proportion** | **Type I fiber numerical proportion** |
| --- | --- | --- | --- | --- | --- |
| GF ​​BH​​ | 27.25±4.17 | 30±1.53 | 29.09±1.88 | 26.14% | 30.64% |
| SPF ​​BH​​ | 23.70±2.27 | 29.4±3.12 | 28.28±3.18 | 10.88% | 19.79% |
| GF ​​TB | 23.31±2.89 | 26.73±3.1 | 25.99±3.21 | 15.50% | 20.06% |
| SPF ​​TB | 25.66±3.88 | 30.98±3.05 | 29.98±2.77 | 13.86% | 18.72% |
| GF ​​FDP​​ | 21.32±4.04 | 26.44±3.37 | 25.66±3.65 | 8.74% | 13.69% |
| SPF ​​FDP​​ | 22.99±2.02 | 30.42±4.31 | 29.18±4.00 | 9.71% | 15.97% |
| GF EDL | 20.97±3.94^a^ | 24.81±1.05^A^ | 24.12±1.12^a^ | 19.48% | 24.03% |
| SPF EDL | 30.23±3.80^b^ | 31.52±4.2^B^ | 30.85±3.84^b^ | 38.07% | 40.10% |
| GF ​​PP | 25.25±1.68^a^ | 26.74±1.73 | 26.18±1.77 | 28.91% | 31.20% |
| SPF ​​PP | 27.16±4.86^b^ | 29.42±4.33 | 28.65±4.6 | 31.09% | 34.25% |
| GF ​​PM​ | 23.95±2.63 | 26.71±1.71 | 26.28±2.26 | 17.52% | 21.70% |
| SPF ​​PM​ | 25.79±3.18 | 30.36±4.88 | 29.67±4.50 | 10.27% | 14.00% |
| GF LDM​​ | 25.96±3.25 | 29.13±2.71 | 28.90±2.69 | 5.76% | 7.11% |
| SPF LDM​​ | 26.78±0.91 | 28.66±0.69 | 28.58±0.64 | 4.57% | 5.77% |
| GF RA | 28.07±3.88 | 33.36±5.96 | 31.33±5.11 | 29.80% | 37.75% |
| SPF RA | 35.34±2.25 | 40.9±5.23 | 38.83±3.86 | 28.56% | 35.06% |
| GF BF​​ | 23.99±3.37 | 29.91±1.95 | 29.11±1.84 | 15.42% | 16.85% |
| SPF BF​​ | 24.10±0.53 | 28.74±2.24 | 27.36±1.54 | 20.83% | 27.31% |
| GF SOL | 24.30±2.72 | 32.35±1.14 | 31.03±1.68 | 9.23% | 15.85% |
| SPF SOL | 24.26±1.14 | 32.31±2.84 | 31.18±2.87 | 8.56% | 14.41% |
| GF GAS​​ | 23.47±1.28 | 28.04±1.29 | 27.31±0.95 | 11.66% | 16.01% |
| SPF GAS​​ | 24.11±0.59 | 29.12±0.95 | 27.90±0.91 | 18.06% | 28.29% |
| GF ​​MF​​ | 25.43±3.30 | 29.55±2.42 | 29.31±2.39 | 4.58% | 6.06% |
| SPF ​​MF​​ | 25.97±6.47 | 30.11±4.77 | 29.62±4.92 | 7.67% | 10.64% |
| GF ​​ADD​​ | 23.85±0.43 | 28.92±4.33 | 28.3±4.04 | 10.48% | 13.85% |
| SPF ​​ADD​​ | 29.4±3.48 | 34.03±4.66 | 33.4±4.26 | 9.18% | 12.28% |

**Supplementary Table 2. Short-chain fatty acid composition in muscle tissue of SPF and GF pigs. unit:μg/g**

| **Sample ID** | **Acetate** | **Propionate** | **Isobutyrate** | **Butyrate** | **Valerate** |
| --- | --- | --- | --- | --- | --- |
| GF MAS | 3.135±0.070^A^ | 0.247±0.002 | / | 0.129±0.013^a^ | 0.015±0.002 |
| SPF MAS | 5.457±0.348^B^ | 0.266±0.057 | 0.064±0.010 | 0.174±0.012^b^ | 0.017±0.002 |
| GF TB | 3.069±0.553 | 0.188±0.016 | / | 0.103±0.011 | 0.012±0.002 |
| SPF TB | 4.145±0.424 | 0.173±0.040 | 0.038±0.002 | 0.129±0.014 | 0.009±0.002 |
| GF PP​​ | 3.753±0.444 | 0.328±0.036^A^ | 0.104±0.011 | 0.151±0.043 | 0.023±0.004^A^ |
| SPF PP​​ | 3.050±0.252 | 0.124±0.031^B^ | 0.011±0.002 | 0.118±0.010 | 0.007±0.001^B^ |
| GF RA​​ | 5.326±0.635^a^ | 0.186±0.060 | / | 0.177±0.041 | 0.012±0.001 |
| SPF RA​​ | 3.166±0.740^b^ | 0.259±0.039 | 0.054±0.006 | 0.117±0.016 | 0.012±0.003 |
| GF PM | 2.607±0.569 | **0.115±0.037^a^** | 0.037±0.007 | 0.075±0.011 | **0.011±0.002^A^** |
| SPF PM | 4.103±0.972 | **0.344±0.116^b^** | 0.082±0.010 | 0.257±0.054 | **0.024±0.003^B^** |
| GF ​​GAS​​ | 2.989±0.898 | 0.216±0.045 | 0.038±0.011 | 0.138±0.031 | **0.012±0.002^A^** |
| SPF ​​GAS​​ | 2.726±0.213 | 0.346±0.077 | 0.064±0.012 | 0.148±0.023 | **0.022±0.003^B^** |

**Note:** No letters showed no significant difference (*P* > 0.05), different lowercase letters showed significant difference (*P* < 0.05), and different uppercase letters showed extremely significant difference (*P* < 0.01). Values are mean ± SEM.

**Supplementary Table 3. The amino acid composition in muscle tissue of SPF and GF pigs (Part Ⅰ) Unit: μg/g**

| **AA**  **Sample** | **L-HIS** | **L-ARG** | **L-ASN** | **GLN** | **L-SER** | **GLY** | **L-ASP** | **L-GLU** | **L-THR** | **L-ALA** |
| --- | --- | --- | --- | --- | --- | --- | --- | --- | --- | --- |
|  | **HIS** | **ARG** | **ASN** | **GLN** | **SER** | **GLY** | **ASP** | **GLU** | **THR** | **ALA** |
| GF MAS | 42.90±3.67**^A^** | 57.46±1.61 | 50.16±8.65**^A^** | 1021.95±210.41**^a^** | 78.92±13.42 | 226.15±62.78 | 88.42±6.29**^a^** | 415.93±37.28**^A^** | 84.72±12.10 | 416.81±54.44**^a^** |
| SPF MAS | 27.30±2.27**^B^** | 58.79±5.44 | 25.41±3.02**^B^** | 469.13±67.36**^b^** | 61.73±7.74 | 178.53±25.46 | 33.71±8.34**^b^** | 110.26±9.42**^B^** | 63.64±11.45 | 257.48±23.15**^b^** |
| GF TB | 37.94±2.80**^A^** | 45.36±5.71 | 43.55±5.4**^A^** | 783.30±148.54**^A^** | 55.37±12.87 | 226.06±48.33 | 109.14±15.15**^A^** | 426.31±51.46**^A^** | 62.45±9.35 | 469.76±113.59**^a^** |
| SPF TB | 20.82±4.00**^B^** | 41.36±3.82 | 26.63±2.08**^B^** | 307.88±50.77**^B^** | 42.22±3.71 | 188.18±50.49 | 23.78±5.07**^B^** | 76.25±18.13**^B^** | 40.30±10.58 | 276.80±34.29**^b^** |
| GF PP | 24.38±1.24 | 68.28±3.78**^a^** | 28.77±3.62 | 642.61±211.77 | 54.44±12.02 | 168.15±41.47 | 40.74±5.93**^a^** | 152.79±35.98 | 52.99±3.28 | 342.43±71.20 |
| SPF PP | 19.96±3.45 | 54.55±4.06**^b^** | 29.66±5.58 | 559.00±316.75 | 52.65±6.91 | 200.82±57.23 | 24.32±2.28**^b^** | 120.93±17.15 | 52.07±3.32 | 351.48±47.48 |
| GF PM | 25.10±1.14**^A^** | 54.30±1.18**^a^** | 39.45±9.40 | 674.47±108.04**^a^** | 53.26±12.83 | 280.60±47.88 | 34.82±4.35**^a^** | 153.30±53.33 | 51.15±6.01 | 402.42±34.62 |
| SPF PM | 19.23±1.67**^B^** | 42.23±4.29**^b^** | 38.02±7.02 | 437.04±96.83**^b^** | 46.29±7.93 | 334.95±57.85 | 19.25±4.27**^b^** | 91.71±13.08 | 46.66±5.97 | 394.60±77.58 |
| GF RA | 34.76±5.29**^A^** | 76.68±3.05**^A^** | 37.64±9.44 | 983.84±135.22**^A^** | 69.45±9.21 | 191.39±65.36 | 83.43±7.79**^A^** | 303.84±43.18**^A^** | 60.83±11.81 | 497.70±59.30**^A^** |
| SPF RA | 16.22±0.78**^B^** | 59.03±5.54**^B^** | 21.43±5.35 | 190.54±36.32**^B^** | 48.08±12.61 | 165.13±50.45 | 16.40±4.77**^B^** | 114.57±20.75**^B^** | 43.10±7.74 | 239.33±29.66**^B^** |
| GF GAS | 26.54±2.87 | 46.74±7.45 | 31.05±4.57 | 638.75±119.83**^a^** | 57.23±7.13**^a^** | 334.95±48.24**^a^** | 34.60±7.22**^a^** | 103.23±23.38 | 46.15±9.35 | 434.24±62.21**^a^** |
| SPF GAS | 19.75±3.26 | 40.76±3.71 | 23.04±4.52 | 301.96±53.55**^b^** | 35.45±6.61**^b^** | 210.67±42.20**^b^** | 16.78±3.47**^b^** | 89.04±8.48 | 36.60±5.55 | 269.30±49.19**^b^** |

**The amino acid composition in muscle tissue of SPF and GF pigs (Part Ⅱ) Unit: μg/g**

| **AA**  **Sample** | **L-PRO** | **L-LYS** | **L-CYS** | **L-TYR** | **L-MET** | **L-VAL** | **L-ILE** | **L-LEU** | **L-PHE** | **L-TRP** |
| --- | --- | --- | --- | --- | --- | --- | --- | --- | --- | --- |
|  | **PRO** | **LYS** | **CYS** | **TYR** | **MET** | **VAL** | **ILE** | **LEU** | **PHE** | **TRP** |
| GF MAS | 94.60±9.70**^A^** | 38.66±2.70**^a^** | 8.25±1.81**^a^** | 29.66±3.70 | 14.93±1.94 | 42.82±4.53 | 24.10±2.54 | 54.23±4.04 | 31.32±1.96 | 10.01±0.62 |
| SPF MAS | 52.15±7.54**^B^** | 27.92±4.51**^b^** | 4.82±1.10**^b^** | 24.48±2.99 | 12.24±1.65 | 35.48±4.40 | 19.35±3.16 | 47.07±5.84 | 28.55±3.81 | 11.28±1.36 |
| GF TB | 67.74±6.61**^a^** | 48.41±8.72 | 4.62±0.77 | 27.36±1.55 | 15.32±3.76 | 52.14±4.67 | 28.30±2.83 | 59.01±3.25 | 32.66±7.67 | 10.75±0.82 |
| SPF TB | 44.58±8.11**^b^** | 42.37±10.12 | 3.09±0.71 | 23.68±2.34 | 15.60±4.42 | 43.10±9.83 | 24.58±3.68 | 52.73±8.90 | 32.90±6.11 | 11.64±3.57 |
| GF PP | 51.04±8.36 | 34.88±9.38 | 7.05±1.61 | 25.96±3.44 | 13.73±2.65 | 38.27±5.59 | 23.28±4.62 | 49.96±6.27 | 31.18±3.40 | 10.11±2.28 |
| SPF PP | 54.17±5.35 | 29.91±6.39 | / | 25.85±2.06 | 15.90±3.03 | 35.63±5.82 | 22.00±3.95 | 45.28±4.30 | 29.20±2.23 | 10.19±2.17 |
| GF PM | 50.67±5.66 | 52.61±7.14**^a^** | 9.69±2.63**^a^** | 30.10±4.21 | 21.83±3.44^a^ | 52.31±9.98 | 32.71±4.15 | 61.28±10.74 | 38.46±7.18 | 12.50±2.17 |
| SPF PM | 50.07±5.87 | 34.03±6.13**^b^** | 2.78±0.39**^b^** | 28.09±2.02 | 29.63±3.09^b^ | 43.27±3.69 | 30.25±4.10 | 58.27±6.72 | 39.75±8.07 | 11.60±1.22 |
| GF RA | 55.44±6.02 | 33.03±3.81 | 1.78±0.25 | 23.65±3.54**^a^** | 15.29±1.60**^A^** | 39.28±4.36 | 23.37±2.92 | 52.99±1.07 | 30.34±0.60**^a^** | 9.95±0.12 |
| SPF RA | 53.35±6.10 | 27.88±5.69 | 1.85±0.74 | 35.51±4.49**^b^** | 23.44±2.14**^B^** | 35.68±4.27 | 25.47±2.61 | 54.46±5.42 | 38.39±4.75**^b^** | 12.22±2.47 |
| GF GAS | 53.82±4.46 | 38.81±5.27 | 2.15±0.37 | 26.09±4.05 | 18.37±3.06 | 43.63±3.03 | 26.66±3.56 | 53.47±10.54 | 34.05±1.75**^a^** | 10.89±0.75 |
| SPF GAS | 44.58±8.87 | 29.45±4.34 | 1.60±0.52 | 32.71±3.81 | 23.12±2.66 | 42.28±2.23 | 28.60±5.50 | 58.44±8.67 | 40.71±2.21**^b^** | 11.99±2.63 |

**Supplementary Table 4. The organic acid composition in muscle tissue of SPF and GF pigs (Part Ⅰ) Unit: μg/g**

| **OA**  **Sample** | **Pyruvate** | **Fumarate** | **Succinate** | **Malate** | **Oxaloacetate** | **α-AKG** | **Citrate** |
| --- | --- | --- | --- | --- | --- | --- | --- |
| GF MAS | 15.791±3.003**^a^** | 64.976±4.743**^a^** | 0.088±0.021 | 108.011±14.157 | 3.513±0.384**^A^** | 7.742±1.106**^A^** | 103.070±10.142**^a^** |
| SPF MAS | 6.789±0.620**^b^** | 54.871±3.759**^b^** | 0.070±0.017 | 87.321±13.002 | 0.984±0.108**^B^** | 1.654±0.467**^B^** | 135.158±11.474**^b^** |
| GF TB | 18.168±3.160**^a^** | 69.585±8.106**^a^** | 0.106±0.018 | 111.869±14.029**^a^** | 4.362±0.956**^a^** | 8.139±0.679**^A^** | 86.033±7.262 |
| SPF TB | 5.628±1.253**^b^** | 48.870±5.119**^b^** | / | 82.630±10.523**^b^** | 0.564±0.134**^b^** | 0.752±0.078**^B^** | 74.526±7.090 |
| GF PP | 6.484±0.686 | 58.913±5.621 | 0.073±0.023 | 103.751±10.944 | 0.767±0.169 | 1.374±0.289 | 126.650±12.621 |
| SPF PP | 6.310±0.451 | 64.573±4.953 | 0.205±0.010 | 107.022±9.760 | 0.588±0.067 | 1.460±0.304 | 130.061±15.647 |
| GF PM | 7.799±0.882**^A^** | 66.554±8.407 | 0.092±0.055 | 115.078±11.271 | 0.644±0.116**^a^** | 1.345±0.549 | 82.702±5.394 |
| SPF PM | 4.580±0.756**^B^** | 61.613±5.267 | 0.033±0.008 | 115.674±15.094 | 0.343±0.068**^b^** | 0.686±0.146 | 69.139±11.242 |
| GF RA | 10.890±1.257**^a^** | 91.367±7.219**^A^** | / | 155.071±8.592**^A^** | 1.801±0.225**^A^** | 4.071±0.611**^a^** | 140.039±9.982**^A^** |
| SPF RA | 7.243±0.439**^b^** | 49.155±5.243**^B^** | 0.505±0.006 | 95.797±7.039**^B^** | 0.287±0.081**^B^** | 0.994±0.113**^b^** | 89.087±4.756**^B^** |
| GF GAS | 12.875±1.511 | 74.632±8.994**^a^** | / | 128.781±18.182**^a^** | 1.378±0.233**^A^** | 1.291±0.399 | 84.525±6.610**^A^** |
| SPF GAS | 10.067±1.123 | 45.430±4.902**^b^** | 0.299±0.035 | 89.437±12.250**^b^** | 0.518±0.157**^B^** | 1.254±0.359 | 46.836±3.216**^B^** |

**The organic acid composition in muscle tissue of SPF and GF pigs (Part Ⅱ) Unit: μg/g**

| **OA**  **Sample** | **Lactate** | **Oxalic acid** | **Malonic acid** | **Methyl malonic acid** | **Glyoxlic acid** | **Glycolic acid** | **β-Hydroxybutyric acid** |
| --- | --- | --- | --- | --- | --- | --- | --- |
| GF MAS | 3441.329±255.543 | 1.730±0.376 | 0.140±0.022 | 0.031±0.004 | 0.204±0.046 | 0.204±0.013 | 3.042±0.635 |
| SPF MAS | 6443.393±836.570 | 1.183±0.032 | 0.141±0.048 | 0.024±0.002 | 0.245±0.025 | 0.168±0.025 | 2.475±0.076 |
| GF TB | 2943.760±61.962**^a^** | 2.024±0.277 | 0.174±0.033 | 0.045±0.008 | 0.168±0.034 | 0.269±0.010 | 2.702±0.636**^a^** |
| SPF TB | 9205.885±1186.504**^b^** | 1.857±0.498 | 0.194±0.020 | 0.041±0.010 | 0.152±0.047 | 0.209±0.031 | 4.129±0.328**^b^** |
| GF PP | 6565.650±1019.243 | 1.414±0.141 | 0.172±0.041 | 0.027±0.009 | 0.309±0.059**^a^** | 0.188±0.039 | 2.104±0.266 |
| SPF PP | 6922.764±1129.438 | 1.612±0.206 | 0.111±0.010 | 0.041±0.008 | 0.128±0.034**^b^** | 0.158±0.011 | 2.320±0.308 |
| GF PM | 9233.124±842.723 | 1.750±0.246 | 0.186±0.025 | 0.059±0.004 | 0.102±0.029**^a^** | 0.247±0.041**^a^** | 3.619±0.384**^a^** |
| SPF PM | 10496.104±1530.123 | 1.280±0.127 | 0.179±0.004 | 0.061±0.017 | 0.311±0.061**^b^** | 0.161±0.022**^b^** | 4.556±0.428**^b^** |
| GF RA | 6602.642±490.466**^A^** | 1.474±0.135 | 0.119±0.012 | 0.039±0.001 | 0.173±0.089 | 0.170±0.037 | 2.857±0.283 |
| SPF RA | 11047.661±939.091**^B^** | 1.184±0.165 | 0.160±0.025 | 0.043±0.007 | 0.222±0.033 | 0.113±0.012 | 2.082±0.551 |
| GF GAS | 8955.617±803.916**^a^** | 1.299±0.111 | 0.187±0.018 | 0.060±0.009 | 0.192±0.040 | 0.237±0.021 | 5.948±0.812 |
| SPF GAS | 12112.869±1103.567**^b^** | 1.503±0.438 | 0.172±0.013 | 0.076±0.011 | 0.292±0.070 | 0.246±0.057 | 4.781±0.163 |

**Supplementary Table 5. The free fatty acids composition in muscle tissue of SPF and GF pigs (Part Ⅰ) Unit: μg/g.**

| **FFA**  **Sample** | **FA11:0** | **FA12:0** | **FA14:1** | **FA14:0** | **FA15:0** | **FA16:0** | **FA16:1** | **FA17:0** | **FA17:1** |
| --- | --- | --- | --- | --- | --- | --- | --- | --- | --- |
| GF MAS | 0.269±0.016 | 1.947±0.848 | 1.139±0.201 | 15.87±4.759 | 1.624±0.219 | 114.476±3.424 | 33.458±10.195 | 2.167±0.092 | 1.906±0.288 |
| SPF MAS | 0.312±0.113 | 3.606±2.880 | 1.271±0.539 | 19.381±11.433 | 2.076±1.131 | 126.526±54.952 | 32.178±14.816 | 3.999±2.247 | 3.268±1.776 |
| GF TB | 0.348±0.131 | 2.532±2.191 | 1.767±1.330 | 13.535±6.576 | 1.455±0.275 | 119.621±50.027 | 31.375±12.235 | 1.892±0.732 | 1.557±0.681 |
| SPF TB | 0.276±0.014 | 2.291±1.764 | 1.455±0.469 | 13.258±6.415 | 1.647±0.145 | 89.641±13.850 | 25.903±8.399 | 2.294±0.446 | 2.258±1.144 |
| GF PP | 0.217±0.040 | 1.121±0.505 | 0.750±0.170 | 8.167±3.437 | 1.117±0.217 | 62.191±7.447 | 22.350±10.255 | 0.915±0.079 | 0.943±0.313 |
| SPF PP | 0.237±0.023 | 1.589±0.936 | 1.022±0.433 | 10.834±4.422 | 1.616±0.383 | 82.897±23.603 | 23.814±9.771 | 2.393±1.177 | 2.010±1.069 |
| GF PM | 0.225±0.030 | 1.703±1.157 | 1.222±0.970 | 10.449±4.650 | 1.219±0.215 | 80.968±19.507 | 21.267±7.507 | 1.373±0.468 | 1.157±0.418 |
| SPF PM | 0.219±0.024 | 1.341±0.757 | 1.096±0.576 | 10.060±4.178 | 1.576±0.471 | 89.771±19.201 | 22.382±6.544 | 2.811±1.368 | 1.899±0.942 |
| GF RA | 0.294±0.015**^A^** | 4.796±2.517 | 1.937±0.597 | 25.325±5.373 | 1.960±1.197 | 124.756±9.105**^A^** | 44.628±2.209**^A^** | 1.935±0.289 | 2.277±0.636 |
| SPF RA | 0.222±0.010**^B^** | 3.058±2.530 | 0.898±0.322 | 16.141±8.392 | 1.205±0.120 | 77.848±12.087**^B^** | 25.041±6.283**^B^** | 1.891±0.490 | 2.168±0.654 |
| GF GAS | 0.289±0.083 | 2.219±0.997 | 1.311±0.445 | 13.030±3.995 | 1.727±0.361 | 85.814±12.928 | 26.946±7.481 | 1.459±0.250 | 1.523±0.321 |
| SPF GAS | 0.194±0.005 | 1.450±0.682 | 1.256±0.736 | 9.752±3.608 | 1.518±0.474 | 74.474±19.003 | 19.700±5.303 | 2.359±1.304 | 1.762±0.698 |

**The free fatty acids composition in muscle tissue of SPF and GF pigs (Part Ⅱ) Unit: μg/g**

| **FFA**  **Sample** | **FA18:0** | **FA18:1** | **FA18:2** | **FA18:3** | **FA 20:0** | **FA 20:1** | **FA 20:2** | **FA 20:3** | **FA 20:4** |
| --- | --- | --- | --- | --- | --- | --- | --- | --- | --- |
| GF MAS | 62.156±4.346 | 159.743±28.889 | 193.102±27.147 | 29.329±5.732 | 1.471±0.114 | 7.965±0.749 | 10.545±2.013 | 18.770±1.355 | 38.735±3.601 |
| SPF MAS | 68.071±24.142 | 175.960±65.464 | 223.622±99.611 | 30.768±18.929 | 1.350±0.378 | 9.570±2.220 | 13.358±3.443 | 22.373±9.078 | 50.333±14.589 |
| GF TB | 53.904±24.938 | 144.675±69.736 | 232.391±119.648 | 31.696±17.737 | 1.707±1.065 | 6.699±4.822 | 8.183±6.219 | 16.079±9.488 | 42.018±25.672 |
| SPF TB | 42.946±7.687 | 120.404±41.863 | 156.031±25.221 | 18.714±7.997 | 1.114±0.174 | 5.934±2.030 | 7.388±2.561**^a^** | 11.413±2.432 | 29.892±6.793 |
| GF PP | 30.144±3.800 | 90.456±24.558 | 101.801±11.767 | 11.660±2.645 | 0.813±0.133 | 2.904±0.851 | 3.022±0.583**^b^** | 6.344±1.001 | 17.211±1.987 |
| SPF PP | 39.288±7.870 | 110.025±36.141 | 139.367±39.640 | 15.285±6.845 | 0.988±0.245 | 4.482±1.213 | 5.382±1.186 | 9.973±3.164 | 26.532±7.144 |
| GF PM | 38.601±10.134 | 100.025±30.129 | 133.680±38.148 | 17.877±6.309 | 1.069±0.462 | 4.648±2.427 | 4.180±2.306 | 8.486±2.713 | 19.555±3.405 |
| SPF PM | 40.869±9.069 | 107.391±31.788 | 154.410±30.987 | 18.876±6.684 | 1.228±0.425 | 4.527±1.862 | 5.571±1.843 | 10.139±2.704 | 27.766±4.999 |
| GF RA | 58.248±10.343**^a^** | 193.697±23.962 | 196.924±19.199**^A^** | 31.245±5.962**^a^** | 1.671±0.583 | 8.747±3.844 | 9.761±4.310 | 14.423±2.290 | 32.325±3.542 |
| SPF RA | 40.857±6.616**^b^** | 129.570±24.019 | 126.264±7.195**^B^** | 15.420±3.877**^b^** | 1.081±0.244 | 6.367±2.334 | 7.632±2.264 | 10.326±1.880 | 24.400±0.509 |
| GF GAS | 40.519±5.852 | 114.935±24.449 | 147.871±28.240 | 17.755±3.285 | 1.295±0.536 | 4.825±1.857 | 5.375±1.918 | 10.041±2.128 | 24.744±4.755 |
| SPF GAS | 37.034±9.343 | 102.437±23.044 | 124.923±33.911 | 13.698±6.519 | 1.023±0.351 | 4.647±1.409 | 5.647±1.372 | 9.367±2.721 | 23.608±3.985 |

**The free fatty acids composition in muscle tissue of SPF and GF pigs (Part Ⅲ) Unit: μg/g**

| **FFA**  **Sample** | **FA 20:5** | **FA 21:0** | **FA 22:0** | **FA 22:1** | **FA 22:2** | **FA 22:3** | **FA 22:4** | **FA 22:5** | **FA 22:6** |
| --- | --- | --- | --- | --- | --- | --- | --- | --- | --- |
| GF MAS | 2.234±0.322 | 1.295±0.068 | 0.252±0.023 | 0.459±0.038 | 0.449±0.066 | 2.116±0.400 | 16.735±3.048 | 6.157±0.661 | 3.411±0.037 |
| SPF MAS | 2.884±1.048 | 1.379±0.032 | 0.221±0.045 | 0.390±0.114 | 0.426±0.105 | 1.916±0.758 | 19.518±5.964 | 8.081±3.264 | 3.145±1.956 |
| GF TB | 2.882±2.236 | 1.436±0.043 | 0.396±0.199 | 0.532±0.334 | 0.427±0.301 | 1.792±1.178 | 15.207±10.468 | 7.247±3.085 | 4.156±1.201 |
| SPF TB | 1.490±0.324 | 1.382±0.251 | 0.227±0.027 | 0.296±0.012 | 0.241±0.014 | 0.806±0.115 | 8.583±1.928 | 6.140±2.104 | 2.949±0.832 |
| GF PP | 0.967±0.106 | 1.260±0.218 | 0.192±0.026 | 0.298±0.054 | 0.138±0.027 | 0.476±0.081 | 4.363±1.014 | 3.128±0.563 | 2.445±0.243 |
| SPF PP | 1.366±0.607 | 1.347±0.091 | 0.212±0.039 | 0.273±0.087 | 0.206±0.052 | 0.734±0.232 | 8.758±3.627 | 6.091±2.527 | 3.574±2.253 |
| GF PM | 1.059±0.267 | 1.187±0.156 | 0.237±0.038 | 0.323±0.141 | 0.220±0.117 | 0.735±0.034 | 5.775±2.671 | 3.155±1.047 | 2.470±0.660 |
| SPF PM | 1.438±0.443 | 1.453±0.319 | 0.257±0.040 | 0.317±0.112 | 0.246±0.089 | 0.867±0.332 | 8.427±2.264 | 5.489±1.706 | 2.911±1.414 |
| GF RA | 1.594±0.442**^a^** | 1.491±0.201 | 0.314±0.097 | 0.488±0.188 | 0.328±0.179 | 1.274±0.609 | 12.445±4.780 | 5.526±1.861 | 5.425±2.557 |
| SPF RA | 0.870±0.074**^b^** | 1.440±0.168 | 0.218±0.019 | 0.303±0.116 | 0.208±0.069 | 0.689±0.241 | 8.036±1.172 | 4.529±0.670 | 2.415±0.670 |
| GF GAS | 1.281±0.388 | 1.746±1.000 | 0.257±0.164 | 0.389±0.169 | 0.227±0.102 | 0.756±0.299 | 7.312±2.157 | 4.523±1.158 | 3.307±0.655 |
| SPF GAS | 1.155±0.520 | 1.316±0.086 | 0.266±0.121 | 0.300±0.097 | 0.230±0.106 | 0.768±0.418 | 7.713±2.613 | 5.158±1.956 | 2.585±1.340 |

**The free fatty acids composition in muscle tissue of SPF and GF pigs (Part Ⅳ) Unit: μg/g**

| **FFA**  **Sample** | **FA 23:0** | **FA 24:0** | **FA 24:1** | **FA 25:0** | **FA 25:1** | **FA 26:0** | **FA 26:1** |
| --- | --- | --- | --- | --- | --- | --- | --- |
| GF MAS | 0.126±0.025 | 0.158±0.033 | 0.132±0.004 | 0.028±0.007 | 0.024±0.003 | 0.029±0.014 | 0.017±0.007 |
| SPF MAS | 0.103±0.033 | 0.121±0.051 | 0.131±0.050 | 0.021±0.007 | 0.020±0.008 | 0.023±0.011 | 0.017±0.002 |
| GF TB | 0.270±0.l44 | 0.434±0.226 | 0.278±0.210 | 0.052±0.026 | 0.065±0.039 | 0.057±0.034 | 0.037±0.022 |
| SPF TB | 0.116±0.021 | 0.165±0.052 | 0.124±0.039 | 0.030±0.001 | 0.026±0.009 | 0.031±0.005 | 0.021±0.008 |
| GF PP | 0.091±0.012 | 0.140±0.024 | 0.065±0.020 | 0.028±0.006 | 0.017±0.003 | 0.028±0.007 | 0.014±0.003 |
| SPF PP | 0.118±0.031 | 0.175±0.068 | 0.107±0.038 | 0.033±0.015 | 0.021±0.007 | 0.039±0.016 | 0.015±0.007 |
| GF PM | 0.118±0.018 | 0.176±0.043 | 0.107±0.052 | 0.028±0.007 | 0.023±0.004 | 0.028±0.008 | 0.015±0.001 |
| SPF PM | 0.141±0.036 | 0.198±0.076 | 0.137±0.069 | 0.030±0.006 | 0.025±0.010 | 0.032±0.010 | 0.018±0.010 |
| GF RA | 0.141±0.015**^a^** | 0.212±0.025**^a^** | 0.145±0.084 | 0.038±0.013 | 0.028±0.008 | 0.046±0.025 | 0.021±0.003 |
| SPF RA | 0.095±0.011**^b^** | 0.133±0.021**^b^** | 0.099±0.025 | 0.030±0.002 | 0.019±0.003 | 0.030±0.004 | 0.017±0.005 |
| GF GAS | 0.138±0.088 | 0.197±0.126 | 0.108±0.064 | 0.036±0.024 | 0.023±0.014 | 0.044±0.024 | 0.021±0.008 |
| SPF GAS | 0.137±0.074 | 0.199±0.117 | 0.119±0.088 | 0.033±0.014 | 0.027±0.019 | 0.038±0.013 | 0.021±0.010 |

**Supplementary Table 6. The fatty acids composition in muscle tissue of SPF and GF pigs (Part Ⅰ) Unit: μg/g.**

| **FA**  **Sample** | **Saturated fatty acids (SFAs)** | | | | | | | |
| --- | --- | --- | --- | --- | --- | --- | --- | --- |
|  | **C6:0** | **C8:0** | **C10:0** | **C11:0** | **C12:0** | **C14:0** | **C15:0** | **C16:0** |
| GF MAS | 0.360±0.126 | 0.710±0.125 | 5.776±0.289 | 0.846±0.151 | 13.821±1.032**^A^** | 108.343±1.239**^a^** | 5.728±0.181 | 1900.539±337.567 |
| SPF MAS | 0.198±0.007 | 0.565±0.103 | 4.733±0.632 | 0.751±0.036 | 26.138±0.930**^B^** | 125.545±7.793**^b^** | 9.600±2.228 | 2330.207±151.131 |
| GF TB | 0.202±0.085 | 0.243±0.037**^A^** | 2.119±0.463**^a^** | 0.661±0.011 | 4.348±1.002 | 44.315±4.313**^A^** | 5.314±0.361 | 1491.223±122.683**^A^** |
| SPF TB | 0.313±0.136 | 0.948±0.093**^B^** | 8.634±1.499**^b^** | 0.724±0.118 | 8.532±1.198 | 91.694±9.172**^B^** | 7.633±1.707 | 3535.231±650.177**^B^** |
| GF PP | 0.232±0.085 | 0.703±0.097**^A^** | 7.013±0.953**^A^** | 0.568±0.076**^a^** | 7.284±1.681 | 47.505±7.608 | 4.790±0.944 | 2142.589±475.246 |
| SPF PP | 0.159±0.045 | 0.324±0.067**^B^** | 3.044±0.495**^B^** | 1.029±0.153**^b^** | 11.445±2.393 | 51.288±2.788 | 6.706±0.858 | 1706.950±512.248 |
| GF PM | 0.296±0.055 | 0.377±0.091 | 2.915±0.261**^a^** | 0.661±0.095 | 9.312±1.281a | 64.102±2.508**^a^** | 4.410±0.758**^a^** | 1643.407±375.760 |
| SPF PM | 0.248±0.042 | 0.488±0.093 | 5.478±0.696**^b^** | 0.713±0.089 | 5.557±0.846b | 79.326±6.235**^b^** | 6.616±1.054**^b^** | 2243.779±132.433 |
| GF RA | 0.639±0.027 | 2.842±0.260**^a^** | 39.071±2.123**^A^** | 0.864±0.176 | 136.447±14.282**^A^** | 535.186±66.286**^a^** | 14.198±1.213 | 9194.643±1092.739**^a^** |
| SPF RA | 0.476±0.078 | 1.975±0.288**^b^** | 19.243±1.003**^B^** | 1.067±0.252 | 78.043±13.310**^B^** | 320.827±38.265**^b^** | 17.698±2.672 | 6552.610±609.074**^b^** |
| GF GAS | 0.256±0.047 | 0.824±0.223 | 8.026±1.986**^a^** | 0.674±0.044 | 30.685±4.984**^a^** | 178.097±39.209**^a^** | 5.935±0.490 | 2617.067±609.074 |
| SPF GAS | 0.166±0.027 | 0.394±0.060 | 3.204±0.441**^b^** | 0.652±0.056 | 9.514±0.869**^b^** | 71.764±7.247**^b^** | 7.700±0.897 | 1829.278±262.631 |

**The fatty acids composition in muscle tissue of SPF and GF pigs (Part Ⅱ) Unit: μg/g**

| **FA**  **Sample** | **Saturated fatty acids (SFAs)** | | | | | | |
| --- | --- | --- | --- | --- | --- | --- | --- |
|  | **C17:0** | **C18:0** | **C20:0** | **C21:0** | **C22：0** | **C23：0** | **C24：0** |
| GF MAS | 19.695±0.983**^a^** | 1519.077±333.082 | 23.236±8.747 | 8.374±0.333 | 14.324±0.190 | 6.657±0.425 | 16.055±0.406 |
| SPF MAS | 54.977±8.629**^b^** | 1838.609±184.179 | 23.932±4.364 | 8.210±0.381 | 13.319±0.674 | 6.215±0.227 | 15.247±0.557 |
| GF TB | 19.056±0.819**^A^** | 1263.325±183.576**^A^** | 18.145±3.902**^A^** | 7.053±0.107 | 15.192±1.609 | 6.441±0.244 | 16.499±0.863 |
| SPF TB | 47.537±6.426**^B^** | 2292.174±103.380**^B^** | 36.888±2.596**^B^** | 7.128±0.453 | 13.820±1.634 | 6.122±0.156 | 15.330±0.823 |
| GF PP | 15.627±5.195**^a^** | 1301.251±369.669 | 24.715±5.333 | 6.358±0.624 | 12.833±0.956 | 6.118±0.323 | 14.673±0.683 |
| SPF PP | 31.066±2.871**^b^** | 1162.590±244.754 | 16.517±5.392 | 6.446±0.648 | 11.502±0.876 | 6.071±0.316 | 13.771±0.730 |
| GF PM | 15.377±2.145**^A^** | 1196.545±273.952 | 22.707±0.700 | 6.173±0.405 | 12.681±1.223 | 5.787±0.364 | 14.575±0.817 |
| SPF PM | 37.520±4.749**^B^** | 1538.763±408.392 | 22.066±2.967 | 6.554±0.560 | 13.270±2.060 | 6.203±0.347 | 15.189±0.405 |
| GF RA | 45.886±5.652**^A^** | 5053.293±763.269 | 110.508±13.944**^a^** | 7.815±1.016 | 18.039±3.529 | 6.600±0.285 | 16.907±0.669 |
| SPF RA | 81.784±8.461**^B^** | 4250.471±291.488 | 70.274±9.818**^b^** | 7.346±0.742 | 14.744±2.984 | 6.230±0.494 | 15.419±0.673 |
| GF GAS | 20.045±4.233**^a^** | 1626.489±327.024 | 29.508±7.818 | 6.544±0.327 | 13.151±0.596 | 5.961±0.276 | 14.805±0.552 |
| SPF GAS | 35.751±6.397**^b^** | 1270.944±210.688 | 17.972±4.008 | 6.403±0.468 | 12.012±1.448 | 5.757±0.373 | 13.851±0.498 |

**The fatty acids composition in muscle tissue of SPF and GF pigs (Part Ⅲ) Unit: μg/g**

| **FA**  **Sample** | **Monounsaturated fatty acids (MUFAs)** | | | | | | |
| --- | --- | --- | --- | --- | --- | --- | --- |
|  | **C15:1** | **C16:1** | **C17:1** | **C18:1n9c** | **C20:1** | **C22：1n9** | **C24:1** |
| GF MAS | 2.202±0.057 | 119.411±79.453 | 8.286±1.782**^a^** | 2361.299±514.614 | 60.995±5.935**^A^** | 7.970±0.277 | / |
| SPF MAS | 2.246±0.013 | 123.432±19.134 | 14.510±1.760**^b^** | 2806.519±281.372 | 88.501±3.943**^B^** | 7.589±0.622 | / |
| GF TB | 2.165±0.148 | 63.787±7.378^A^ | 6.653±0.263 | 1390.592±278.889A | 32.691±2.001**^a^** | 7.179±0.746 | 7.806±0.937 |
| SPF TB | 2.211±0.152 | 218.459±33.765^B^ | 19.918±5.803 | 4191.719±500.854B | 123.061±33.685**^b^** | 7.789±1.854 | 6.200±0.775 |
| GF PP | 2.203±0.190 | 211.380±28.750**^A^** | 6.653±0.263 | 2638.257±466.045 | 44.002±8.204 | 6.424±0.957 | 7.646±0.401 |
| SPF PP | 1.957±0.046 | 102.194±22.626**^B^** | 5.508±0.985 | 1808.759±384.516 | 37.134±6.615 | 6.039±0.714 | 6.469±0.650 |
| GF PM | 2.131±0.016 | 70.851±13.986**^a^** | 6.384±0.612 | 1761.895±385.892 | 50.872±6.459 | 6.526±0.941 | 6.750±0.429 |
| SPF PM | 2.215±0.051 | 119.822±10.781**^b^** | 9.280±1.932 | 2450.190±333.143 | 46.939±11.433 | 11.654±0.732 | / |
| GF RA | 2.055±0.083 | 547.526±138.374 | 22.752±4.539 | 12005.684±2895.412 | 301.826±4.813 | 10.020±1.517 | 11.787±1.362 |
| SPF RA | 2.288±0.073 | 370.265±53.343 | 29.704±6.559 | 8588.344±1041.182 | 240.508±37.088 | 6.272±0.718 | 9.508±1.230 |
| GF GAS | 2.092±0.128 | 200.301±67.441 | 6.950±0.224 | 3504.016±472.953**^a^** | 81.378±12.744**^a^** | 7.081±0.273 | 7.256±0.838 |
| SPF GAS | 1.869±0.036 | 92.648±3.945 | 10.679±2.128 | 2033.931±221.341**^b^** | 51.227±3.088**^b^** | 6.272±0.718 | 6.857±0.603 |

**The fatty acids composition in muscle tissue of SPF and GF pigs (Part Ⅳ) Unit: μg/g**

| **FA**  **Sample** | **Polyunsaturated fatty acids (PUFAs)** | | | | | | | | |
| --- | --- | --- | --- | --- | --- | --- | --- | --- | --- |
|  | **C18：2n6c** | **C18：3n6** | **C18：3n3** | **C20:2** | **C20：3n6** | **C20：4n6** | **C20：3n3** | **C20：5n3** | **C22：6** |
| GF MAS | 2311.860±89.903**^a^** | 12.744±1.888 | 82.404±11.740 | 94.853±8.539**^a^** | 66.417±4.475 | 472.048±21.097 | 25.889±2.910 | 21.952±0.585 | 30.804±2.369 |
| SPF MAS | 2742.472±172.184**^b^** | 9.421±0.851 | 92.384±16.789 | 121.812±11.493**^b^** | 69.562±3.924 | 479.419±17.326 | 30.988±3.820 | 22.078±2.419 | 26.353±3.806 |
| GF TB | 2527.666±233.008**^a^** | 11.216±3.663 | 75.764±5.416**^a^** | 66.977±13.028**^a^** | 51.809±2.601 | 451.740±8.348 | 18.726±2.289 | 21.054±2.675 | 34.035±3.480**^a^** |
| SPF TB | 3288.059±66.400**^b^** | 7.921±1.956 | 132.764±33.307**^b^** | 131.824±29.317**^b^** | 51.894±5.234 | 412.481±69.846 | 34.570±17.948 | 19.792±1.676 | 26.381±2.998**^b^** |
| GF PP | 1982.788±616.600 | 9.962±2.512**^a^** | 81.523±9.678 **^a^** | 62.925±3.573 | 39.124±5.696 | 322.090±5.289 | 18.672±6.558 | 17.964±1.186 | 28.369±3.721 |
| SPF PP | 1985.494±316.044 | 3.864±0.396**^b^** | 55.681±6.195 **^b^** | 61.466±4.910 | 39.880±1.697 | 359.069±43.762 | 18.484±2.870 | 16.713±1.077 | 21.406±2.533 |
| GF PM | 2017.784±359.580 | 8.438±0.850 | 72.157±3.031 | 59.510±15.819 | 40.952±5.036 | 345.398±40.410 | 18.963±2.951 | 18.862±0.979 | 30.550±3.559 |
| SPF PM | 2558.435±531.943 | 6.570±1.141 | 78.245±6.858 | 53.990±9.745 | 42.522±5.628 | 392.760±4.386 | 21.667±8.485 | 18.994±0.866 | 24.453±4.745 |
| GF RA | 4943.135±597.250 | 12.635±2.444**^a^** | 290.435±31.963 **^a^** | 226.155±43.984 | 56.303±3.821 | 364.973±44.046 | 59.080±5.467 | 18.546±1.041 | 34.759±3.899 |
| SPF RA | 3962.404±349.287 | 6.890±0.740**^b^** | 196.025±11.727 **^b^** | 197.878±41.913 | 52.851±1.727 | 441.429±50.111 | 50.665±6.400 | 18.533±1.272 | 29.830±4.561 |
| GF GAS | 2540.985±596.266 | 9.059±1.457**^a^** | 121.073±9.728**^A^** | 88.732±5.169**^a^** | 46.460±3.914 | 352.877±32.618 | 25.165±0.472 | 19.220±0.369 | 30.339±3.713 |
| SPF GAS | 2248.992±257.495 | 4.730±0.799**^b^** | 67.509±9.185**^B^** | 73.053±4.213**^b^** | 45.028±5.321 | 403.679±28.113 | 21.216±1.932 | 18.695±1.982 | 23.394±5.277 |

**Note:** No letters showed no significant difference (*P* > 0.05), different lowercase letters showed significant difference (*P* < 0.05), and different uppercase letters showed extremely significant difference (*P* < 0.01). Values are mean ± SEM.

**Supplementary Table 7. Summary of sequencing data.**

| **Sample ID** | **Raw_reads** | **Raw_bases** | **Clean_reads** | **Clean_bases** |
| --- | --- | --- | --- | --- |
| GF1-YJ | 95650730 | 14.35G | 93910442 | 14.09G |
| GF5-YJ | 90487978 | 13.57G | 88448188 | 13.27G |
| GF6-YJ | 90933696 | 13.64G | 88373996 | 13.26G |
| SPF2-YJ | 91240540 | 13.69G | 89069860 | 13.36G |
| SPF4-YJ | 94198198 | 14.13G | 92351200 | 13.85G |
| SPF6-YJ | 91937162 | 13.79G | 89699824 | 13.45G |
| GF1-GT | 92663630 | 13.9G | 90960158 | 13.64G |
| GF5-GT | 89134784 | 13.37G | 86316236 | 12.95G |
| GF6-GT | 90475128 | 13.57G | 88338302 | 13.25G |
| SPF2-GT | 93385432 | 14.01G | 90114408 | 13.52G |
| SPF4-GT | 85094442 | 12.76G | 82127724 | 12.32G |
| SPF6-GT | 89148690 | 13.37G | 86815550 | 13.02G |
| GF1-GST | 88641054 | 13.3G | 87239024 | 13.09G |
| GF5-GST | 86906024 | 13.04G | 84949148 | 12.74G |
| GF6-GST | 84876404 | 12.73G | 82601700 | 12.39G |
| SPF2-GST | 90482180 | 13.57G | 86822544 | 13.02G |
| SPF4-GST | 96101622 | 14.42G | 93086016 | 13.96G |
| SPF6-GST | 92227630 | 13.83G | 89663270 | 13.45G |
| GF1-ZSQ | 83270434 | 12.49G | 81872796 | 12.28G |
| GF5-ZSQ | 82557614 | 12.38G | 81284282 | 12.19G |
| GF6-ZSQ | 92240776 | 13.84G | 90347444 | 13.55G |
| SPF2-ZSQ | 91539782 | 13.73G | 88897230 | 13.33G |
| SPF4ZSQ | 82470408 | 12.37G | 80571930 | 12.09G |
| SPF6-ZSQ | 83090624 | 12.46G | 80466450 | 12.07G |
| GF1-ZWCS | 85786312 | 12.87G | 83929124 | 12.59G |
| GF5-ZWCS | 91956298 | 13.79G | 90129004 | 13.52G |
| GF6-ZWCS | 92414728 | 13.86G | 90342352 | 13.55G |
| SPF2-ZWCS | 88109832 | 13.22G | 86129104 | 12.92G |
| SPF4ZWCS | 85113914 | 12.77G | 83462538 | 12.52G |
| SPF6-ZWCS | 95108474 | 14.27G | 92599604 | 13.89G |
| GF1-XS | 86779688 | 13.02G | 85027794 | 12.75G |
| GF5-XS | 86487306 | 12.97G | 84784160 | 12.72G |
| GF6-XS | 86936292 | 13.04G | 84622384 | 12.69G |
| SPF2-XS | 103162448 | 15.47G | 101148696 | 15.17G |
| SPF4-XS | 83351082 | 12.5G | 80590766 | 12.09G |
| SPF6-XS | 93738524 | 14.06G | 91603914 | 13.74G |
| GF1-YD | 96396442 | 14.46G | 94293166 | 14.14G |
| GF5-YD | 84253502 | 12.64G | 82132830 | 12.32G |
| GF6-YD | 92760792 | 13.91G | 91506894 | 13.73G |
| SPF2-YD | 94122888 | 14.12G | 90009796 | 13.5G |
| SPF4-YD | 90843224 | 13.63G | 88730876 | 13.31G |
| SPF6-YD | 82813936 | 12.42G | 80908056 | 12.14G |
| GF1-BZC | 92991200 | 13.95G | 90635116 | 13.6G |
| GF5-BZC | 93085672 | 13.96G | 90388064 | 13.56G |
| GF6-BZC | 85969894 | 12.9G | 84336308 | 12.65G |
| SPF2-BZC | 89076514 | 13.36G | 84832050 | 12.72G |
| SPF4-BZC | 86147492 | 12.92G | 84025634 | 12.6G |
| SPF6-BZC | 90599780 | 13.59G | 89052962 | 13.36G |
| GF1-FZ | 90940564 | 13.64G | 88261336 | 13.24G |
| GF5-FZ | 82437590 | 12.37G | 80981456 | 12.15G |
| GF6-FZ | 90402640 | 13.56G | 88237790 | 13.24G |
| SPF2-FZ | 84781604 | 12.72G | 81764878 | 12.26G |
| SPF4-FZ | 86216408 | 12.93G | 84139838 | 12.62G |
| SPF6-FZ | 92105816 | 13.82G | 90105076 | 13.52G |
| GF1-GET | 90704866 | 13.61G | 88159832 | 13.22G |
| GF5-GET | 91713762 | 13.76G | 90129200 | 13.52G |
| GF6-GET | 85969928 | 12.9G | 84423222 | 12.66G |
| SPF2-GET | 85369744 | 12.81G | 83287638 | 12.49G |
| SPF4-GET | 85656486 | 12.85G | 81642892 | 12.25G |
| SPF6-GET | 85865176 | 12.88G | 83662192 | 12.55G |
| GF1-BMY | 86582664 | 12.99G | 84806714 | 12.72G |
| GF5-BMY | 90473804 | 13.57G | 88188016 | 13.23G |
| GF6-BMY | 87417328 | 13.11G | 85841790 | 12.88G |
| SPF2-BMY | 91340882 | 13.7G | 88202528 | 13.23G |
| SPF4-BMY | 85735622 | 12.86G | 80684498 | 12.1G |
| SPF6-BMY | 83036996 | 12.46G | 81578976 | 12.24G |
| GF1-FC | 89579352 | 13.44G | 88026156 | 13.2G |
| GF5-FC | 90513660 | 13.58G | 88564286 | 13.28G |
| GF6-FC | 88816078 | 13.32G | 86949920 | 13.04G |
| SPF2-FC | 89214566 | 13.38G | 85863570 | 12.88G |
| SPF4-FC | 92909152 | 13.94G | 88200554 | 13.23G |
| SPF6-FC | 89135494 | 13.37G | 87833508 | 13.18G |
| GF1-GNC | 94169840 | 14.13G | 91543116 | 13.73G |
| GF5-GNC | 92816568 | 13.92G | 91310924 | 13.7G |
| GF6-GNC | 93348318 | 14G | 91620862 | 13.74G |
| SPF2-GNC | 90839576 | 13.63G | 88173728 | 13.23G |
| SPF4-GNC | 98607772 | 14.79G | 93046342 | 13.96G |
| SPF6-GNC | 91097154 | 13.66G | 82284118 | 12.34G |
| GF1-NS | 93226114 | 13.98G | 91056052 | 13.66G |
| GF5-NS | 86673050 | 13G | 84949258 | 12.74G |
| GF6-NS | 88388968 | 13.26G | 87026818 | 13.05G |
| SPF2-NS | 103276866 | 15.49G | 101122796 | 15.17G |
| SPF4-NS | 83528696 | 12.53G | 81342380 | 12.2G |
| SPF6-NS | 86868442 | 13.03G | 85296048 | 12.79G |

**Note:** The abbreviations for the muscles in this study are defined as follows: YJ (masseter muscle, MAS), GST (triceps brachii, TB), GT (brachial head muscle, BH), ZSQ (flexor digitorum profundus, FDP), ZWCS (extensor digitorum lateralis, EDL), BZC (longissimus dorsi muscle, LDM), FZ (rectus abdominis, RA), XS (pectoralis profundus, PP), YD (psoas major muscle, PM), BMY (soleus muscle, SOL), FC (gastrocnemius muscle, GAS), GET (biceps femoris, BF), GNC (medial femoral muscle, MF), and NS (adductores, ADD).

**Supplementary Table 8. Quality assessment of sequencing data.**

| **Sample ID** | **Error rate (%)** | **Q20 (%)** | **Q30 (%)** | **GC content (%)** |
| --- | --- | --- | --- | --- |
| GF1-YJ | 0.03 | 96.7 | 91.43 | 48.75 |
| GF5-YJ | 0.02 | 98 | 94.35 | 49.42 |
| GF6-YJ | 0.03 | 97.5 | 92.92 | 50.99 |
| SPF2-YJ | 0.03 | 97.38 | 92.72 | 50.27 |
| SPF4-YJ | 0.03 | 97.66 | 93.38 | 49.12 |
| SPF6-YJ | 0.03 | 96.79 | 91.16 | 48.43 |
| GF1-GT | 0.03 | 96.64 | 91.24 | 49.36 |
| GF5-GT | 0.03 | 96.84 | 91.71 | 49.96 |
| GF6-GT | 0.03 | 97.67 | 93.62 | 49.86 |
| SPF2-GT | 0.03 | 97.52 | 93.27 | 49.58 |
| SPF4-GT | 0.03 | 97.72 | 93.42 | 49.12 |
| SPF6-GT | 0.03 | 96.73 | 91.2 | 48.56 |
| GF1-GST | 0.03 | 97.92 | 94.03 | 47.34 |
| GF5-GST | 0.03 | 96.57 | 91.09 | 50.77 |
| GF6-GST | 0.03 | 97.59 | 93.42 | 51.09 |
| SPF2-GST | 0.03 | 97.59 | 93.45 | 49.55 |
| SPF4-GST | 0.03 | 97.71 | 93.45 | 48.71 |
| SPF6-GST | 0.03 | 96.94 | 91.73 | 49.09 |
| GF1-ZSQ | 0.02 | 97.95 | 94.35 | 49.97 |
| GF5-ZSQ | 0.03 | 96.63 | 91.28 | 48.82 |
| GF6-ZSQ | 0.03 | 97.74 | 93.63 | 49.28 |
| SPF2-ZSQ | 0.03 | 97.44 | 92.99 | 48.46 |
| SPF4ZSQ | 0.03 | 97.64 | 93.35 | 50.05 |
| SPF6-ZSQ | 0.03 | 96.56 | 90.85 | 48.61 |
| GF1-ZWCS | 0.03 | 97.33 | 92.63 | 48.18 |
| GF5-ZWCS | 0.03 | 96.91 | 91.64 | 48.03 |
| GF6-ZWCS | 0.03 | 97.54 | 93.34 | 48.53 |
| SPF2-ZWCS | 0.03 | 97.66 | 93.52 | 49.53 |
| SPF4ZWCS | 0.03 | 96.97 | 91.98 | 49.39 |
| SPF6-ZWCS | 0.02 | 98.02 | 94.43 | 49.03 |
| GF1-XS | 0.02 | 98.08 | 94.67 | 49.36 |
| GF5-XS | 0.03 | 96.86 | 91.75 | 49.54 |
| GF6-XS | 0.03 | 97.66 | 93.61 | 50 |
| SPF2-XS | 0.03 | 97.7 | 93.69 | 49.6 |
| SPF4-XS | 0.03 | 97.2 | 92.18 | 50.13 |
| SPF6-XS | 0.03 | 97.4 | 92.92 | 48.93 |
| GF1-YD | 0.03 | 96.67 | 91.24 | 49.48 |
| GF5-YD | 0.03 | 97.21 | 92.42 | 50.07 |
| GF6-YD | 0.03 | 97.15 | 92.11 | 49.14 |
| SPF2-YD | 0.03 | 97.73 | 93.75 | 49.64 |
| SPF4-YD | 0.03 | 97.64 | 93.5 | 49.77 |
| SPF6-YD | 0.03 | 97.34 | 92.73 | 48.43 |
| GF1-BZC | 0.03 | 97.77 | 94.04 | 51.39 |
| GF5-BZC | 0.02 | 98.03 | 94.55 | 50.94 |
| GF6-BZC | 0.03 | 96.96 | 91.77 | 50.22 |
| SPF2-BZC | 0.03 | 97.44 | 93.06 | 49.66 |
| SPF4-BZC | 0.03 | 97.75 | 93.71 | 50.95 |
| SPF6-BZC | 0.03 | 97.27 | 92.68 | 48.71 |
| GF1-FZ | 0.02 | 97.87 | 94.35 | 51.85 |
| GF5-FZ | 0.03 | 96.91 | 91.72 | 48.57 |
| GF6-FZ | 0.03 | 97.5 | 93.09 | 49.2 |
| SPF2-FZ | 0.03 | 97.44 | 93.02 | 50.3 |
| SPF4-FZ | 0.03 | 97.6 | 93.16 | 48.98 |
| SPF6-FZ | 0.03 | 97.48 | 93.06 | 49.96 |
| GF1-GET | 0.03 | 97.82 | 93.96 | 50.4 |
| GF5-GET | 0.03 | 96.64 | 91.29 | 50.09 |
| GF6-GET | 0.03 | 97.51 | 93.09 | 48.65 |
| SPF2-GET | 0.03 | 97.43 | 92.99 | 49.92 |
| SPF4-GET | 0.03 | 97.26 | 92.57 | 52.55 |
| SPF6-GET | 0.03 | 97.48 | 93.03 | 49.23 |
| GF1-BMY | 0.03 | 96.44 | 90.94 | 49.04 |
| GF5-BMY | 0.02 | 98.12 | 94.67 | 51.73 |
| GF6-BMY | 0.03 | 97.16 | 92.19 | 48.82 |
| SPF2-BMY | 0.03 | 97.45 | 93.05 | 49.76 |
| SPF4-BMY | 0.03 | 97.54 | 93.04 | 49.86 |
| SPF6-BMY | 0.03 | 97.29 | 92.55 | 48.16 |
| GF1-FC | 0.03 | 97.76 | 93.81 | 49.01 |
| GF5-FC | 0.02 | 98.02 | 94.47 | 49.47 |
| GF6-FC | 0.03 | 97.31 | 92.53 | 49.11 |
| SPF2-FC | 0.03 | 97.61 | 93.44 | 49.58 |
| SPF4-FC | 0.03 | 97.71 | 93.64 | 49.9 |
| SPF6-FC | 0.03 | 97.42 | 92.94 | 48.46 |
| GF1-GNC | 0.02 | 98.08 | 94.6 | 49.25 |
| GF5-GNC | 0.03 | 96.95 | 91.88 | 49.78 |
| GF6-GNC | 0.03 | 97.81 | 93.93 | 50.45 |
| SPF2-GNC | 0.03 | 97.61 | 93.4 | 48.76 |
| SPF4-GNC | 0.03 | 97.74 | 93.57 | 50.46 |
| SPF6-GNC | 0.04 | 92.49 | 85.86 | 67.84 |
| GF1-NS | 0.02 | 98.09 | 94.65 | 50.03 |
| GF5-NS | 0.03 | 96.68 | 91.34 | 49.65 |
| GF6-NS | 0.03 | 97.44 | 92.98 | 49.55 |
| SPF2-NS | 0.03 | 97.11 | 92.07 | 51.52 |
| SPF4-NS | 0.03 | 97.44 | 92.76 | 49.06 |
| SPF6-NS | 0.03 | 97.22 | 92.5 | 48.36 |

**Note:** The abbreviations for the muscles in this study are defined as follows: YJ (masseter muscle, MAS), GST (triceps brachii, TB), GT (brachial head muscle, BH), ZSQ (flexor digitorum profundus, FDP), ZWCS (extensor digitorum lateralis, EDL), BZC (longissimus dorsi muscle, LDM), FZ (rectus abdominis, RA), XS (pectoralis profundus, PP), YD (psoas major muscle, PM), BMY (soleus muscle, SOL), FC (gastrocnemius muscle, GAS), GET (biceps femoris, BF), GNC (medial femoral muscle, MF), and NS (adductores, ADD).

**Supplementary Table 9. Statistical list of mapping to genome.**

| **Sample ID** | **Paired reads** | **Uniquely**  **Reads** | **Uniquely**  **Mapped (%)** | **MultiReads** | **MultiMapped (%)** | **Total**  **Mapped (%)** |
| --- | --- | --- | --- | --- | --- | --- |
| GF1-YJ | 46955221 | 42022260 | 89.49 | 3726302 | 7.93 | 97.42 |
| GF5-YJ | 44224094 | 38567987 | 87.21 | 4824281 | 10.91 | 98.12 |
| GF6-YJ | 44186998 | 37013725 | 83.77 | 6344678 | 14.36 | 98.13 |
| SPF2-YJ | 44534930 | 38211322 | 85.8 | 5078831 | 11.41 | 97.21 |
| SPF4-YJ | 46175600 | 40239275 | 87.14 | 4148492 | 8.99 | 96.13 |
| SPF6-YJ | 44849912 | 39701533 | 88.52 | 3079222 | 6.86 | 95.38 |
| GF1-GT | 45480079 | 40285785 | 88.58 | 4050887 | 8.91 | 97.49 |
| GF5-GT | 43158118 | 36384841 | 84.31 | 5704422 | 13.22 | 97.53 |
| GF6-GT | 44169151 | 37945850 | 85.91 | 5362930 | 12.14 | 98.05 |
| SPF2-GT | 45057204 | 39642878 | 87.98 | 4358721 | 9.68 | 97.66 |
| SPF4-GT | 41063862 | 35365678 | 86.12 | 4129017 | 10.06 | 96.18 |
| SPF6-GT | 43407775 | 38316371 | 88.27 | 2902872 | 6.68 | 94.95 |
| GF1-GST | 43619512 | 40276371 | 92.34 | 2538203 | 5.82 | 98.16 |
| GF5-GST | 42474574 | 35175167 | 82.81 | 6274557 | 14.78 | 97.59 |
| GF6-GST | 41300850 | 34887338 | 84.47 | 5503910 | 13.32 | 97.79 |
| SPF2-GST | 43411272 | 37927466 | 87.37 | 4299182 | 9.91 | 97.28 |
| SPF4-GST | 46543008 | 40422652 | 86.85 | 4449487 | 9.56 | 96.41 |
| SPF6-GST | 44831635 | 39192202 | 87.42 | 3421217 | 7.63 | 95.05 |
| GF1-ZSQ | 40936398 | 37017095 | 90.43 | 3100267 | 7.57 | 98 |
| GF5-ZSQ | 40642141 | 35736776 | 87.93 | 3903539 | 9.6 | 97.53 |
| GF6-ZSQ | 45173722 | 39489602 | 87.42 | 4921382 | 10.9 | 98.32 |
| SPF2-ZSQ | 44448615 | 39742274 | 89.41 | 3740600 | 8.42 | 97.83 |
| SPF4ZSQ | 40285965 | 35111536 | 87.16 | 4014002 | 9.97 | 97.13 |
| SPF6-ZSQ | 40233225 | 35343403 | 87.85 | 2853589 | 7.09 | 94.94 |
| GF1-ZWCS | 41964562 | 37286899 | 88.85 | 3905003 | 9.3 | 98.15 |
| GF5-ZWCS | 45064502 | 39121164 | 86.81 | 4943604 | 10.97 | 97.78 |
| GF6-ZWCS | 45171176 | 39925110 | 88.39 | 4331919 | 9.59 | 97.98 |
| SPF2-ZWCS | 43064552 | 37878944 | 87.96 | 4256431 | 9.88 | 97.84 |
| SPF4ZWCS | 41731269 | 37471574 | 89.79 | 2959042 | 7.09 | 96.88 |
| SPF6-ZWCS | 46299802 | 41211483 | 89.01 | 2778755 | 6 | 95.01 |
| GF1-XS | 42513897 | 37686000 | 88.64 | 4047130 | 9.52 | 98.16 |
| GF5-XS | 42392080 | 36092595 | 85.14 | 5236015 | 12.35 | 97.49 |
| GF6-XS | 42311192 | 36011297 | 85.11 | 5452166 | 12.89 | 98 |
| SPF2-XS | 50574348 | 44935586 | 88.85 | 4671577 | 9.24 | 98.09 |
| SPF4-XS | 40295383 | 34144223 | 84.73 | 4632334 | 11.5 | 96.23 |
| SPF6-XS | 45801957 | 40623208 | 88.69 | 3480244 | 7.6 | 96.29 |
| GF1-YD | 47146583 | 41538025 | 88.1 | 4446542 | 9.43 | 97.53 |
| GF5-YD | 41066415 | 34682367 | 84.45 | 5491593 | 13.38 | 97.83 |
| GF6-YD | 45753447 | 40064848 | 87.57 | 4795136 | 10.48 | 98.05 |
| SPF2-YD | 45004898 | 39142411 | 86.97 | 4831535 | 10.74 | 97.71 |
| SPF4-YD | 44365438 | 39082654 | 88.09 | 4446966 | 10.02 | 98.11 |
| SPF6-YD | 40454028 | 35608539 | 88.02 | 2696087 | 6.66 | 94.68 |
| GF1-BZC | 45317558 | 39648655 | 87.49 | 4498752 | 9.93 | 97.42 |
| GF5-BZC | 45194032 | 37602428 | 83.2 | 6700449 | 14.83 | 98.03 |
| GF6-BZC | 42168154 | 36473001 | 86.49 | 4689177 | 11.12 | 97.61 |
| SPF2-BZC | 42416025 | 37060876 | 87.37 | 4403470 | 10.38 | 97.75 |
| SPF4-BZC | 42012817 | 35836978 | 85.3 | 5384309 | 12.81 | 98.11 |
| SPF6-BZC | 44526481 | 39474792 | 88.65 | 3277325 | 7.36 | 96.01 |
| GF1-FZ | 44130668 | 34427058 | 78.01 | 8477534 | 19.21 | 97.22 |
| GF5-FZ | 40490728 | 35617134 | 87.96 | 3925818 | 9.7 | 97.66 |
| GF6-FZ | 44118895 | 39105899 | 88.64 | 4081722 | 9.25 | 97.89 |
| SPF2-FZ | 40882439 | 35207256 | 86.12 | 4658358 | 11.39 | 97.51 |
| SPF4-FZ | 42069919 | 36644599 | 87.1 | 3734362 | 8.88 | 95.98 |
| SPF6-FZ | 45052538 | 39562857 | 87.81 | 3867768 | 8.59 | 96.4 |
| GF1-GET | 44079916 | 37761737 | 85.67 | 5456749 | 12.38 | 98.05 |
| GF5-GET | 45064600 | 38336234 | 85.07 | 5593791 | 12.41 | 97.48 |
| GF6-GET | 42211611 | 36696094 | 86.93 | 4722061 | 11.19 | 98.12 |
| SPF2-GET | 41643819 | 36160960 | 86.83 | 4597617 | 11.04 | 97.87 |
| SPF4-GET | 40821446 | 34676250 | 84.95 | 3862318 | 9.47 | 94.42 |
| SPF6-GET | 41831096 | 36873428 | 88.15 | 3649597 | 8.72 | 96.87 |
| GF1-BMY | 42403357 | 37605891 | 88.69 | 3639925 | 8.58 | 97.27 |
| GF5-BMY | 44094008 | 35020202 | 79.42 | 8126621 | 18.43 | 97.85 |
| GF6-BMY | 42920895 | 38323853 | 89.29 | 3671266 | 8.55 | 97.84 |
| SPF2-BMY | 44101264 | 37885603 | 85.91 | 5257506 | 11.92 | 97.83 |
| SPF4-BMY | 40342249 | 34472973 | 85.45 | 4343012 | 10.77 | 96.22 |
| SPF6-BMY | 40789488 | 36045032 | 88.37 | 2566134 | 6.29 | 94.66 |
| GF1-FC | 44013078 | 39655261 | 90.1 | 3452947 | 7.84 | 97.94 |
| GF5-FC | 44282143 | 39006678 | 88.09 | 4388514 | 9.91 | 98 |
| GF6-FC | 43474960 | 38158841 | 87.77 | 4432221 | 10.2 | 97.97 |
| SPF2-FC | 42931785 | 37617046 | 87.62 | 4390861 | 10.23 | 97.85 |
| SPF4-FC | 44100277 | 37858364 | 85.85 | 4813609 | 10.91 | 96.76 |
| SPF6-FC | 43916754 | 39106686 | 89.05 | 2682353 | 6.11 | 95.16 |
| GF1-GNC | 45771558 | 40316539 | 88.08 | 4588830 | 10.02 | 98.1 |
| GF5-GNC | 45655462 | 39332079 | 86.15 | 5287731 | 11.58 | 97.73 |
| GF6-GNC | 45810431 | 37015044 | 80.8 | 8026597 | 17.52 | 98.32 |
| SPF2-GNC | 44086864 | 39117481 | 88.73 | 4062503 | 9.22 | 97.95 |
| SPF4-GNC | 46523171 | 39480988 | 84.86 | 5607613 | 12.05 | 96.91 |
| SPF6-GNC | 41142059 | 29399548 | 71.46 | 4096110 | 9.95 | 81.41 |
| GF1-NS | 45528026 | 39666759 | 87.13 | 4969419 | 10.92 | 98.05 |
| GF5-NS | 42474629 | 36201232 | 85.23 | 5203377 | 12.25 | 97.48 |
| GF6-NS | 43513409 | 37818013 | 86.91 | 4839996 | 11.12 | 98.03 |
| SPF2-NS | 50561398 | 42105440 | 83.28 | 6825383 | 13.5 | 96.78 |
| SPF4-NS | 40671190 | 35176874 | 86.49 | 3115611 | 7.66 | 94.15 |
| SPF6-NS | 42648024 | 37956010 | 89 | 3416307 | 8.01 | 97.01 |

**Note:** The abbreviations for the muscles in this study are defined as follows: YJ (masseter muscle, MAS), GST (triceps brachii, TB), GT (brachial head muscle, BH), ZSQ (flexor digitorum profundus, FDP), ZWCS (extensor digitorum lateralis, EDL), BZC (longissimus dorsi muscle, LDM), FZ (rectus abdominis, RA), XS (pectoralis profundus, PP), YD (psoas major muscle, PM), BMY (soleus muscle, SOL), FC (gastrocnemius muscle, GAS), GET (biceps femoris, BF), GNC (medial femoral muscle, MF), and NS (adductores, ADD).

## **Supplementary Table 10. Differential expression of LncRNA and its cis differentially expressed PCG in muscle tissues of GF and SPF pigs.**

| **Lnc ID** | **PCG Ensembl ID** | **Cor** | ***P* value** | **Gene name** |
| --- | --- | --- | --- | --- |
| G31795 | ENSSSCG00000000274 | 0.71 | <0.01 | PCBP2 |
| G39185 | ENSSSCG00000001916 | 0.80 | <0.01 | INSYN1 |
| G40047 | ENSSSCG00000002383 | 0.91 | <0.01 | FOS |
| G34513 | ENSSSCG00000003006 | 0.73 | <0.01 | CYP2B22 |
| G35652 | ENSSSCG00000003439 | 0.71 | <0.01 | DHRS3 |
| G35713 | ENSSSCG00000003514 | 0.73 | <0.01 | HSPG2 |
| G36125 | ENSSSCG00000003672 | 0.72 | <0.01 | CAP1 |
| G36821 | ENSSSCG00000003812 | 0.86 | <0.01 | PGM1 |
| G925 | ENSSSCG00000004255 | 0.93 | <0.01 | VGLL2 |
| G869 | ENSSSCG00000004255 | 0.75 | <0.01 | VGLL2 |
| G870 | ENSSSCG00000004255 | 0.73 | <0.01 | VGLL2 |
| G2153 | ENSSSCG00000004803 | 0.74 | <0.01 | ACTC1 |
| G2808 | ENSSSCG00000005039 | 0.82 | <0.01 | STYX |
| G4659 | ENSSSCG00000005719 | 0.81 | <0.01 | RAPGEF1 |
| G19854 | ENSSSCG00000007231 | 0.73 | <0.01 | MYLK2 |
| G25817 | ENSSSCG00000007798 | 0.84 | <0.01 | TBC1D10B |
| G7081 | ENSSSCG00000009535 | 0.84 | <0.01 | EFNB2 |
| G13394 | ENSSSCG00000009881 | 0.81 | <0.01 | OAS2 |
| G14722 | ENSSSCG00000010461 | 0.79 | <0.01 | ANKRD1 |
| G5555 | ENSSSCG00000011076 | 0.97 | <0.01 | OTUD1 |
| G10291 | ENSSSCG00000011521 | 0.71 | <0.01 | PDZRN3 |
| G45328 | ENSSSCG00000015595 | 0.80 | <0.01 | ATF3 |
| G16592 | ENSSSCG00000015901 | 0.90 | <0.01 | GRB14 |
| G42289 | ENSSSCG00000025240 | 0.86 | <0.01 | DDIT4L |
| G42477 | ENSSSCG00000030548 | 0.88 | <0.01 | HERC5 |
| G8546 | ENSSSCG00000031087 | 0.97 | <0.01 | NA |
| G23270 | ENSSSCG00000031539 | 0.87 | <0.01 | NA |
| G22743 | ENSSSCG00000031940 | 0.73 | <0.01 | GAS2 |
| G46460 | ENSSSCG00000032395 | 0.98 | <0.01 | NA |
| G23270 | ENSSSCG00000033136 | 0.77 | <0.01 | NA |
| G7198 | ENSSSCG00000033620 | 0.74 | <0.01 | NA |
| G11329 | ENSSSCG00000034802 | 0.80 | <0.01 | NA |
| G45034 | ENSSSCG00000035055 | 0.94 | <0.01 | NA |
| G21868 | ENSSSCG00000035293 | 0.71 | <0.01 | NA |
| G18081 | ENSSSCG00000036201 | 0.85 | <0.01 | NPR3 |
| G14450 | ENSSSCG00000036976 | 0.95 | <0.01 | NA |
| G7193 | ENSSSCG00000037039 | 0.90 | <0.01 | PPP1R27 |
| G31268 | ENSSSCG00000037958 | 0.72 | <0.01 | TOB2 |
| G25198 | ENSSSCG00000038453 | 0.76 | <0.01 | SYNPO |
| G31797 | ENSSSCG00000039506 | 0.96 | <0.01 | MYL6 |

**Supplementary Table 11. Numbers of differentially regulated lncRNA-PCG pairs.**

| **Muscle** | **Total lncRNA-PCG pairs** | **Co-upregulated pairs** | **Co-downregulated pairs** |
| --- | --- | --- | --- |
| BMY | 2 | 0 | 2 |
| BZC | 7 | 4 | 3 |
| FC | 6 | 2 | 4 |
| FZ | 3 | 2 | 1 |
| GET | 3 | 1 | 2 |
| GNC | 2 | 2 | 0 |
| GST | 1 | 0 | 1 |
| GT | 4 | 1 | 3 |
| NS | 2 | 1 | 1 |
| XS | 3 | 1 | 2 |
| YD | 4 | 1 | 3 |
| YJ | 3 | 1 | 2 |
| ZWCS | 4 | 4 | 0 |

**Note:** The abbreviations for the muscles in this study are defined as follows: YJ (masseter muscle, MAS), GST (triceps brachii, TB), GT (brachial head muscle, BH), ZSQ (flexor digitorum profundus, FDP), ZWCS (extensor digitorum lateralis, EDL), BZC (longissimus dorsi muscle, LDM), FZ (rectus abdominis, RA), XS (pectoralis profundus, PP), YD (psoas major muscle, PM), BMY (soleus muscle, SOL), FC (gastrocnemius muscle, GAS), GET (biceps femoris, BF), GNC (medial femoral muscle, MF), and NS (adductores, ADD).

**Supplementary Table 12. Differentially expressed lncRNAs and their co-regulated protein-coding genes in GF vs SPF pigs across multiple tissues.**

| **Lnc_ID** | **PCG_name ID** | **regulate_Lnc** | **regulate_PCG** | **tissue_name** | **PCG_name** |
| --- | --- | --- | --- | --- | --- |
| G42289 | ENSSSCG00000025240 | DOWN in GF | DOWN in GF | BMY | DDIT4L |
| G7193 | ENSSSCG00000037039 | DOWN in GF | DOWN in GF | BMY | PPP1R27 |
| G13394 | ENSSSCG00000009881 | DOWN in GF | DOWN in GF | BZC | OAS2 |
| G14450 | ENSSSCG00000036976 | UP in GF | UP in GF | BZC | / |
| G14722 | ENSSSCG00000010461 | DOWN in GF | DOWN in GF | BZC | ANKRD1 |
| G25198 | ENSSSCG00000038453 | UP in GF | UP in GF | BZC | SYNPO |
| G46460 | ENSSSCG00000032395 | UP in GF | UP in GF | BZC | / |
| G7081 | ENSSSCG00000009535 | UP in GF | UP in GF | BZC | EFNB2 |
| G7193 | ENSSSCG00000037039 | DOWN in GF | DOWN in GF | BZC | PPP1R27 |
| G13394 | ENSSSCG00000009881 | DOWN in GF | DOWN in GF | FC | OAS2 |
| G14722 | ENSSSCG00000010461 | DOWN in GF | DOWN in GF | FC | ANKRD1 |
| G18081 | ENSSSCG00000036201 | UP in GF | UP in GF | FC | NPR3 |
| G45328 | ENSSSCG00000015595 | DOWN in GF | DOWN in GF | FC | ATF3 |
| G7193 | ENSSSCG00000037039 | DOWN in GF | DOWN in GF | FC | PPP1R27 |
| G8546 | ENSSSCG00000031087 | UP in GF | UP in GF | FC | / |
| G13394 | ENSSSCG00000009881 | DOWN in GF | DOWN in GF | FZ | OAS2 |
| G34513 | ENSSSCG00000003006 | UP in GF | UP in GF | FZ | CYP2B6 |
| G8546 | ENSSSCG00000031087 | UP in GF | UP in GF | FZ | / |
| G13394 | ENSSSCG00000009881 | DOWN in GF | DOWN in GF | GET | OAS2 |
| G18081 | ENSSSCG00000036201 | UP in GF | UP in GF | GET | NPR3 |
| G7193 | ENSSSCG00000037039 | DOWN in GF | DOWN in GF | GET | PPP1R27 |
| G8546 | ENSSSCG00000031087 | UP in GF | UP in GF | GNC | / |
| G925 | ENSSSCG00000004255 | UP in GF | UP in GF | GNC | VGLL2 |
| G13394 | ENSSSCG00000009881 | DOWN in GF | DOWN in GF | GST | OAS2 |
| G13394 | ENSSSCG00000009881 | DOWN in GF | DOWN in GF | GT | OAS2 |
| G2153 | ENSSSCG00000004803 | DOWN in GF | DOWN in GF | GT | ACTC1 |
| G39185 | ENSSSCG00000001916 | DOWN in GF | DOWN in GF | GT | INSYN1 |
| G8546 | ENSSSCG00000031087 | UP in GF | UP in GF | GT | / |
| G13394 | ENSSSCG00000009881 | DOWN in GF | DOWN in GF | NS | OAS2 |
| G8546 | ENSSSCG00000031087 | UP in GF | UP in GF | NS | / |
| G39185 | ENSSSCG00000001916 | DOWN in GF | DOWN in GF | XS | INSYN1 |
| G42289 | ENSSSCG00000025240 | DOWN in GF | DOWN in GF | XS | DDIT4L |
| G8546 | ENSSSCG00000031087 | UP in GF | UP in GF | XS | / |
| G13394 | ENSSSCG00000009881 | DOWN in GF | DOWN in GF | YD | OAS2 |
| G34513 | ENSSSCG00000003006 | UP in GF | UP in GF | YD | CYP2B6 |
| G42477 | ENSSSCG00000030548 | DOWN in GF | DOWN in GF | YD | HERC5 |
| G45034 | ENSSSCG00000035055 | DOWN in GF | DOWN in GF | YD | / |
| G11329 | ENSSSCG00000034802 | DOWN in GF | DOWN in GF | YJ | RTP4 |
| G13394 | ENSSSCG00000009881 | DOWN in GF | DOWN in GF | YJ | OAS2 |
| G36821 | ENSSSCG00000003812 | UP in GF | UP in GF | YJ | PGM1 |
| G16592 | ENSSSCG00000015901 | UP in GF | UP in GF | ZWCS | GRB14 |
| G40047 | ENSSSCG00000002383 | UP in GF | UP in GF | ZWCS | FOS |
| G5555 | ENSSSCG00000011076 | UP in GF | UP in GF | ZWCS | OTUD1 |
| G8546 | ENSSSCG00000031087 | UP in GF | UP in GF | ZWCS | / |

**Supplementary Fig. 1 Constructing sterile testing indicators for GF pig model.**


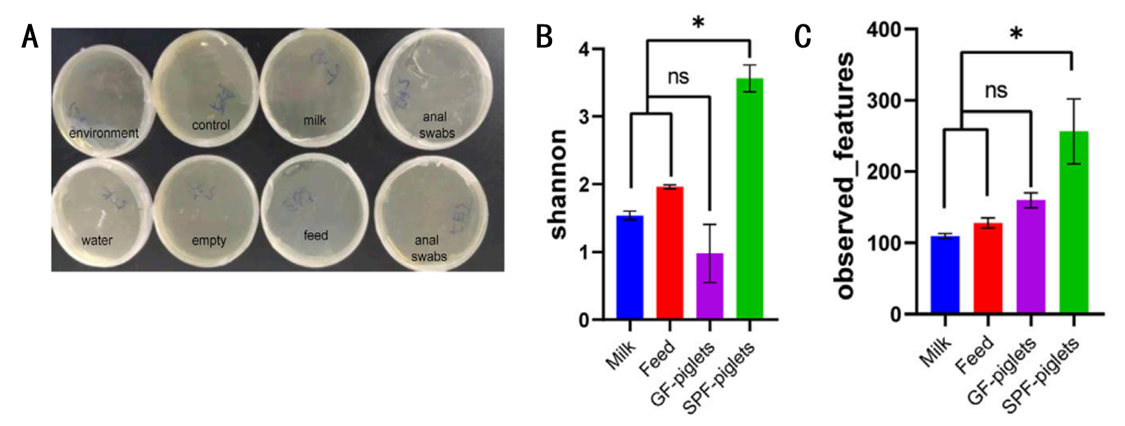


**Supplementary Fig. 1 Constructing sterile testing indicators for GF pig model. (A)** Medium samples were used to detect microbiota in the feeding isolator, including in the environment, feed, milk powder, and piglet anal swabs. **(B)** and **(C)** Microbiota detection in feed, milk powder, and piglet anal swabs by 16s rDNA amplicon sequencing. Shannon and observed features indices reﬂect the α diversity of the microbiota (n = 3). (Reprinted from Zhang et al., Front. Physiol. 14, 1084332 (2023), with permission).

­­­­

**Supplementary Fig. 2 Free fatty acid (FFA) and fatty acid (FA) content in the muscle tissues of SPF and GF pigs.**

**
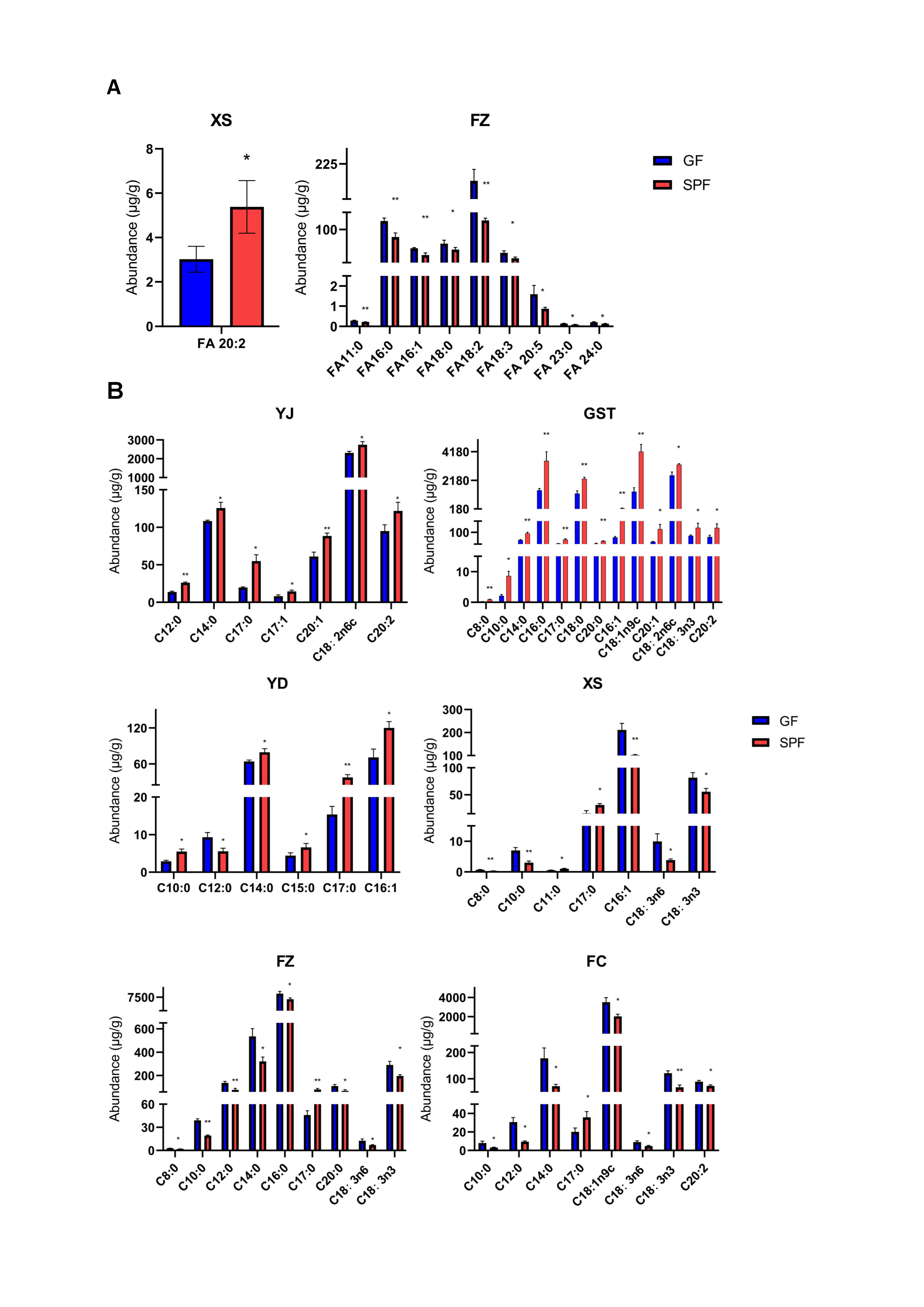
**

**Supplementary Fig. 2 Free fatty acid (FFA) and fatty acid (FA) content in the muscle tissues of SPF and GF pigs.**​​ **(A)** FFA content in muscle tissues from different anatomical locations of SPF and GF pigs. (**B**) FA content in muscle tissues from different anatomical locations of SPF and GF pigs. Abbreviations: YJ (masseter muscle, MAS), GST (triceps brachii, TB), YD (psoas major muscle, PM), XS (pectoralis profundus, PP), FZ (rectus abdominis, RA), FC (gastrocnemius muscle, GAS).

**Supplementary Fig. 3 Pearson correlation analysis of PCG, LncRNA, and TUCP expression levels in muscle tissues between GF and SPF pigs.​**


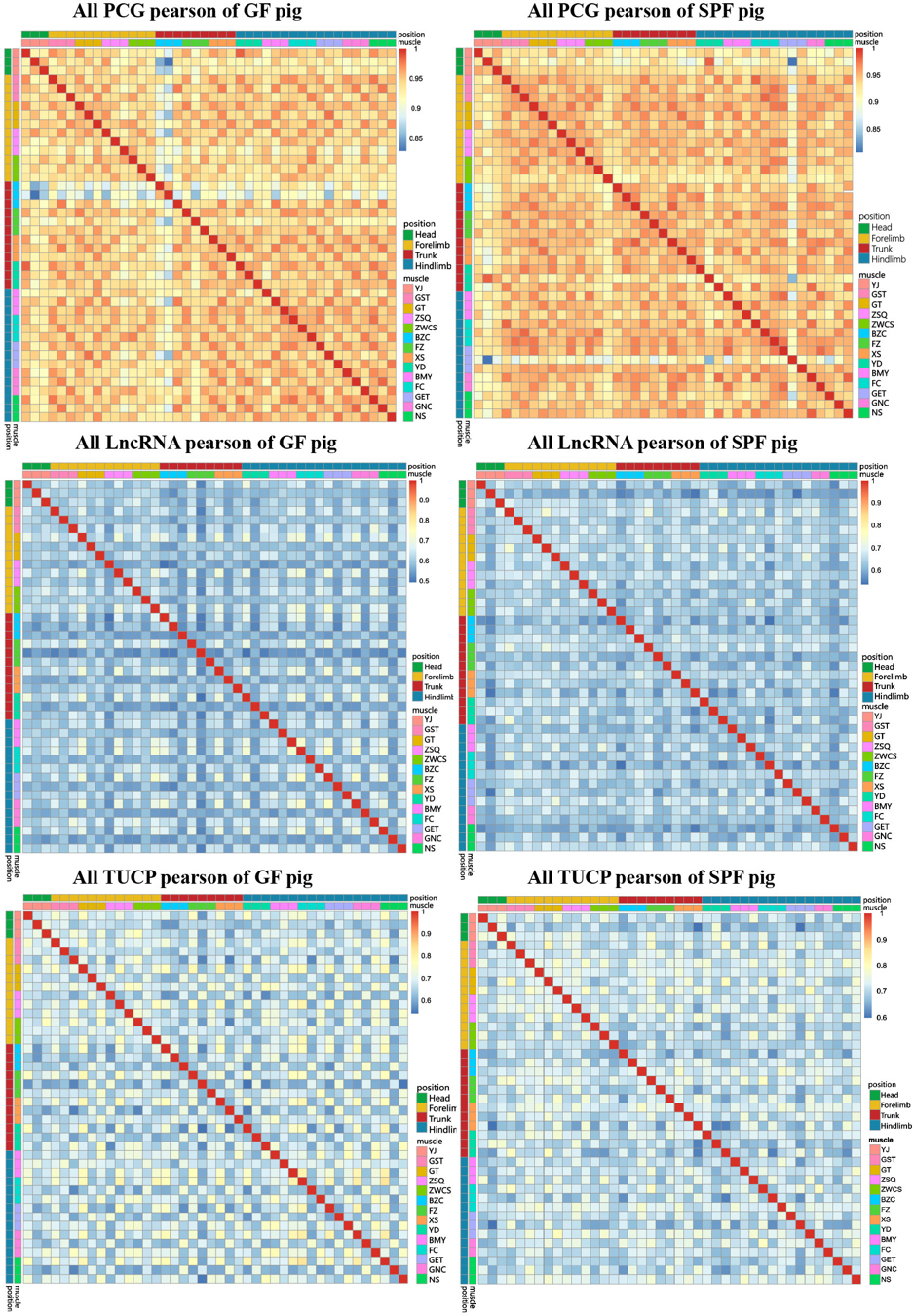


**Supplementary Fig. 3 Pearson correlation analysis of PCG, LncRNA, and TUCP expression levels in muscle tissues between GF and SPF pigs.**

**Supplementary Fig. 4 Transcript expression patterns and functional analysis in muscle tissues of GF and SPF pigs.**


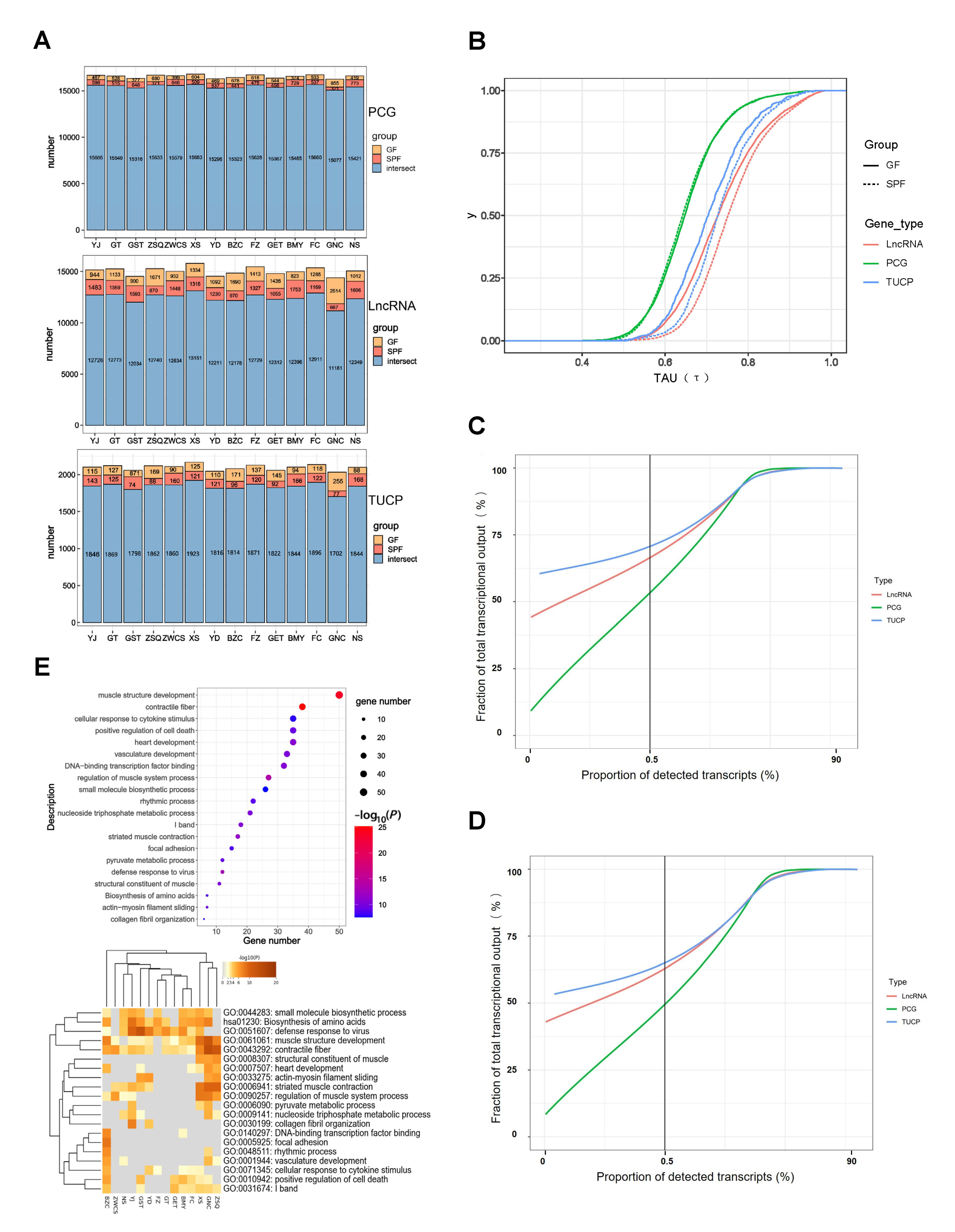


**Supplementary Fig. 4 Transcript expression patterns and functional analysis in muscle tissues of GF and SPF pigs.** **(A)** Expression profiles of PCGs, LncRNAs, and TUCPs in muscle tissues of GF and SPF pigs. **(B)** Tissue specificity TAU (τ) scores of different transcripts. **(C, D)** Abundance distribution of transcripts in muscle tissues of GF (C) and SPF (D) pigs. The x-axis represents the proportion of transcripts ranked from highest to lowest abundance, with the vertical solid line indicating the top 0.5% most abundant transcripts. The y-axis shows the cumulative proportion of transcripts relative to the total transcriptome. **(E)** GO enrichment analysis of differentially expressed genes in muscle tissues between GF and SPF pigs.

**Supplementary Fig. 5 Regulatory analysis of Homeobox gene family and myokines expression in muscle tissues of GF versus SPF pigs.​​**


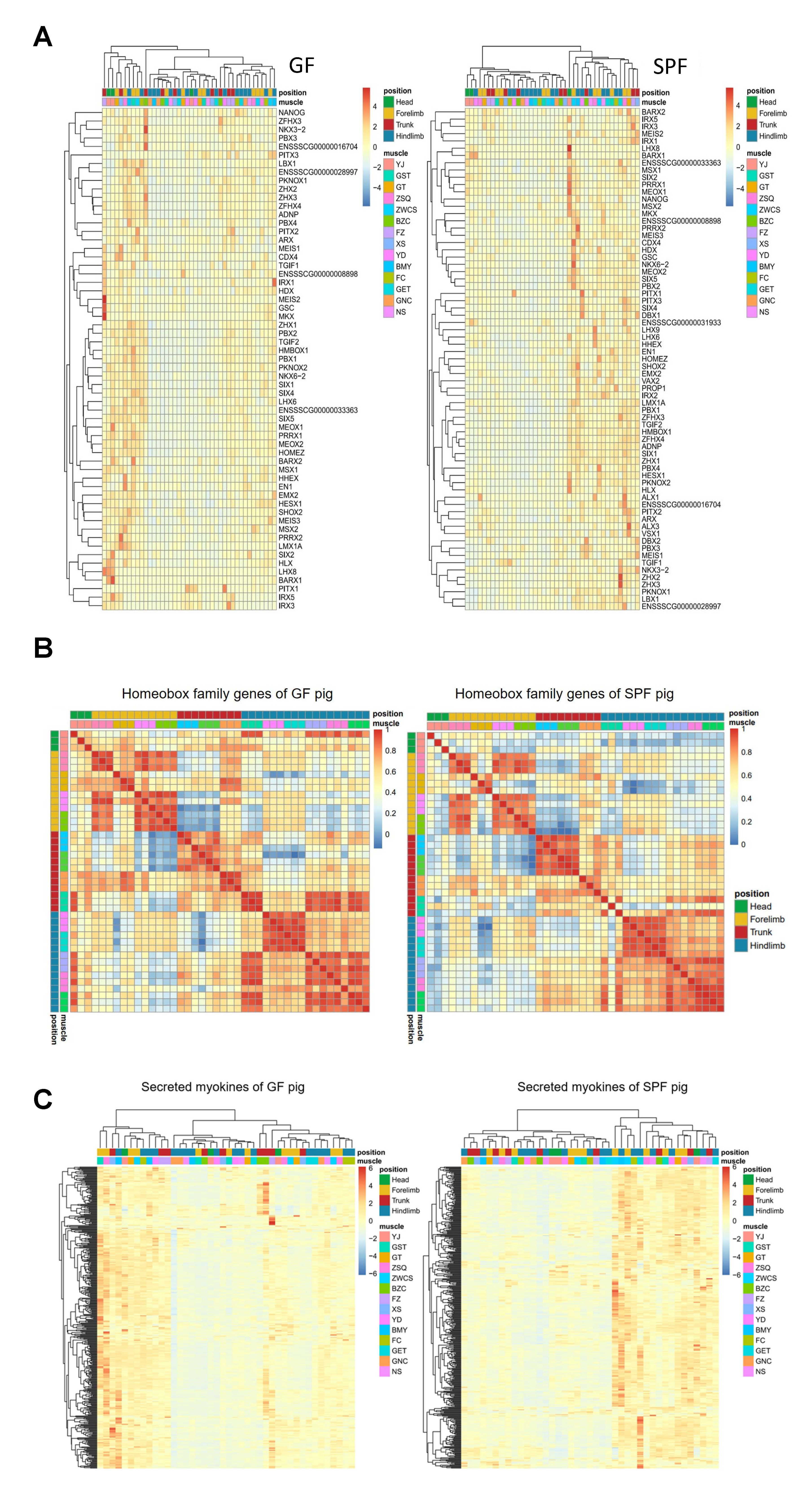


**Supplementary Fig. 5 Regulatory analysis of Homeobox gene family and myokines expression in muscle tissues of GF versus SPF pigs.​​ (A)** Expression profiles of Homeobox family genes in muscle tissues of GF and SPF pigs. **(B)** Correlation of expression patterns among Homeobox family genes in GF and SPF pigs. **(C)** Transcriptional patterns of myokines in muscle tissues of GF and SPF pigs.

­

**Supplementary Fig. 6 GO pathway enrichment in Module 0-6.**


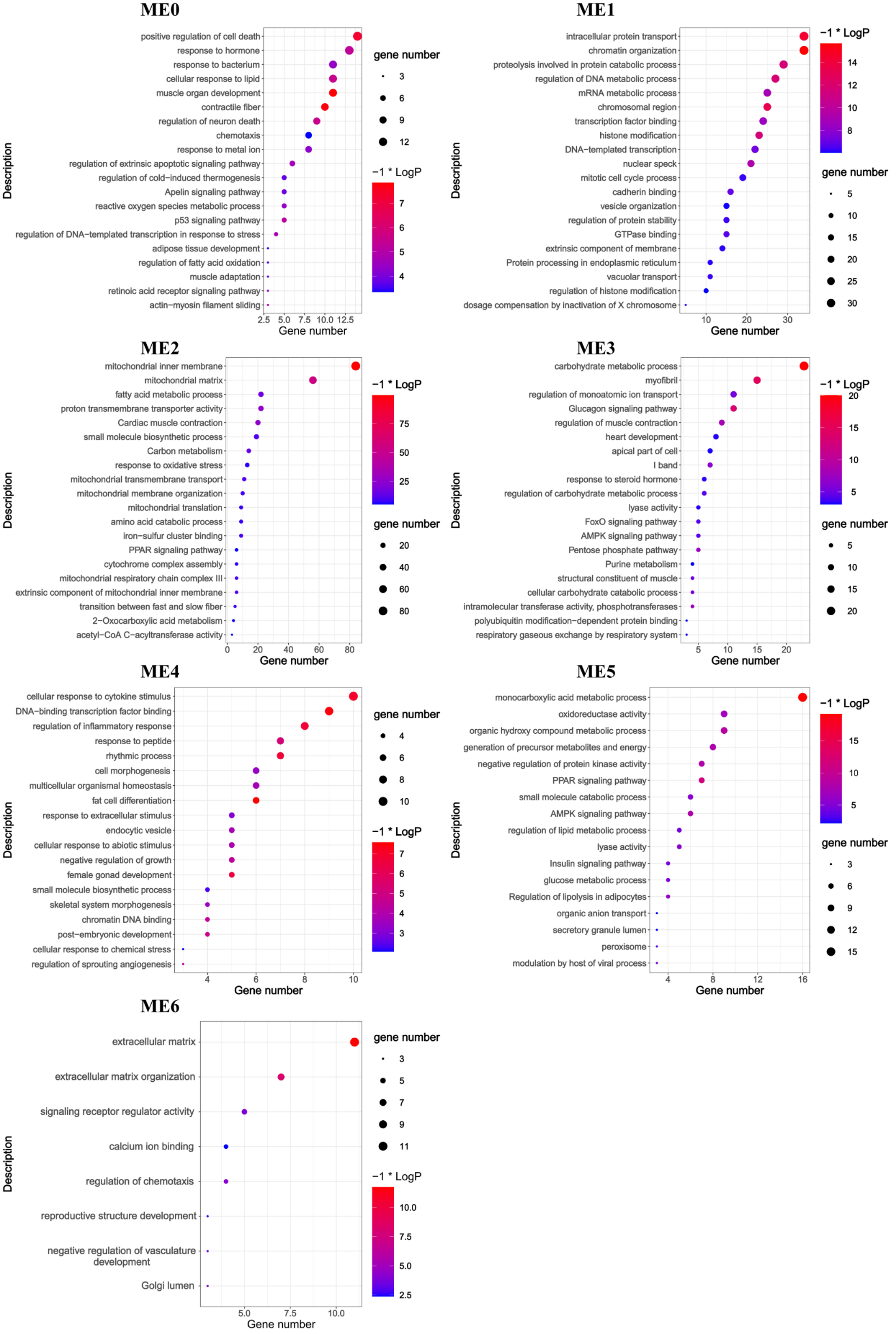


**Supplementary Fig. 6 GO pathway enrichment in Module 0-6.**

**Supplementary Fig. 7 Association analysis between co-expression network modules and fatty acid metabolism in muscle tissues.​​**


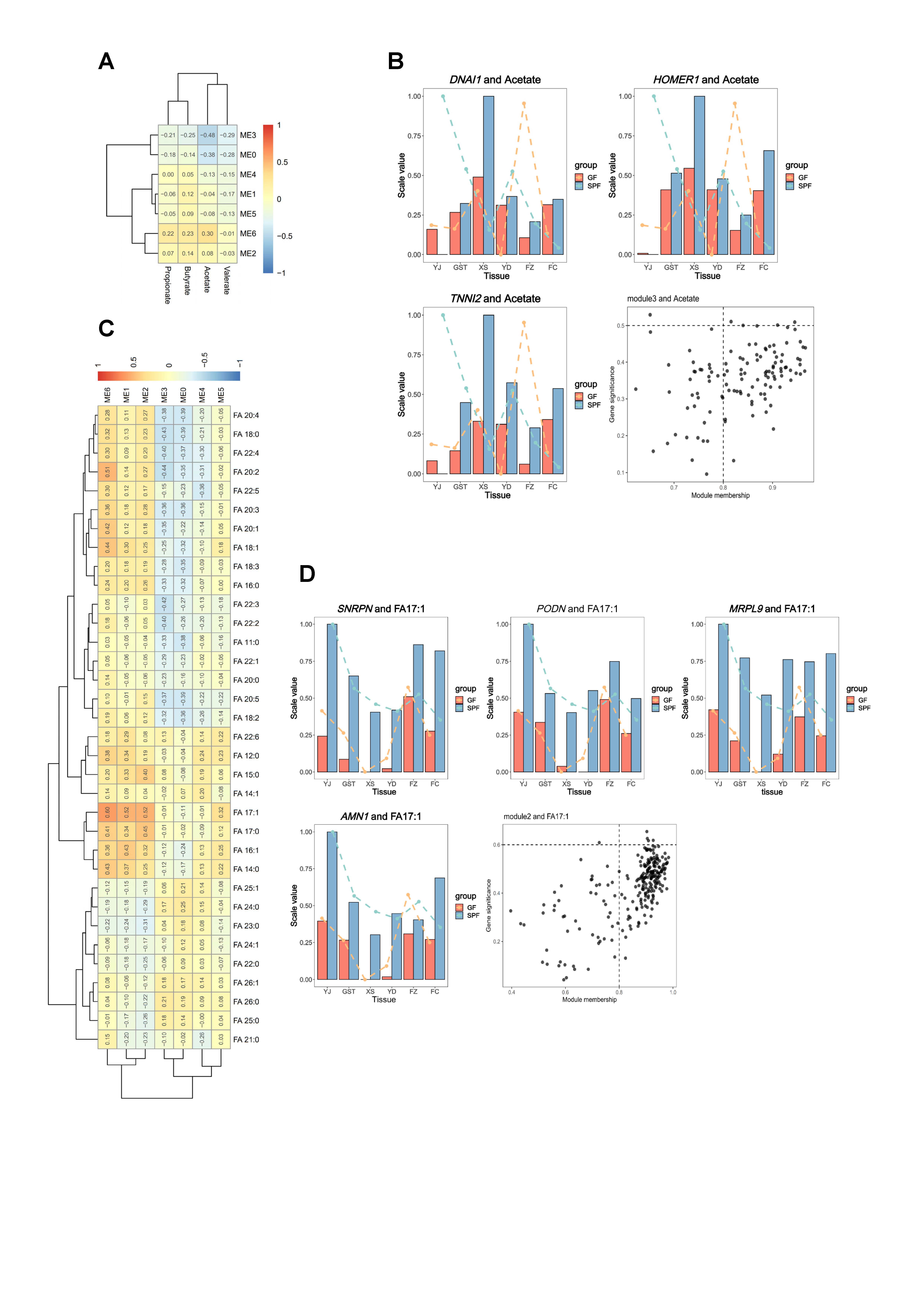


**Supplementary Fig. 7 Association analysis between co-expression network modules and fatty acid metabolism in muscle tissues.​​ (A)** Correlation between modules and short-chain fatty acid (SCFA) content. **(B)** Gene significance for acetate and distribution of ME3 members. **(C)** Correlation between modules and free fatty acid (FFA) content. **(D)** Gene significance for FA 17:1 and distribution of ME2 members. Abbreviations: YJ (masseter muscle, MAS), GST (triceps brachii, TB), XS (pectoralis profundus, PP), YD (psoas major muscle, PM), FZ (rectus abdominis, RA), FC (gastrocnemius muscle, GAS). Bar plots represent gene expression levels (normalized values), lines indicate phenotypic values (normalized scale), and black dots denote genes. The same representation applies to subsequent figures.

**Supplementary Fig. 8 Regulatory relationships between muscle tissue co-expression network modules and organic acid/fatty acid metabolism.​​**

**
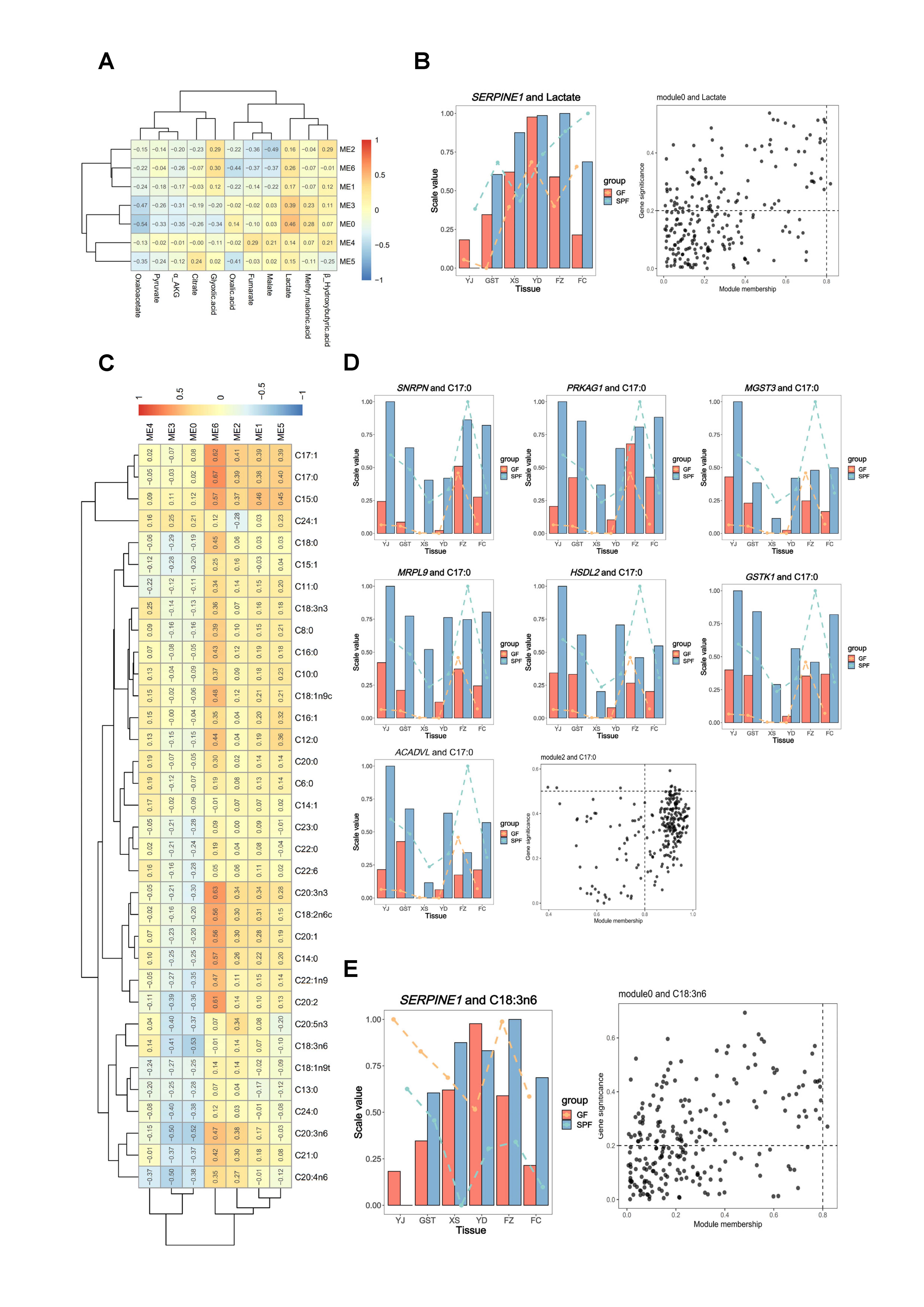
**

**Supplementary Fig. 8 Regulatory relationships between muscle tissue co-expression network modules and organic acid/fatty acid metabolism.​​ (A)** Module-organic acid content correlations. **(B)** Lactate gene significance and ME0 module member distribution. **(C)** Module-fatty acid content correlations. **(D)** C17:0 gene significance and ME2 module member distribution. **(E)** C18:3n6 gene significance and ME0 module member distribution. Muscle tissue abbreviations: YJ (masseter muscle, MAS), GST (triceps brachii, TB), XS (pectoralis profundus, PP), YD (psoas major muscle, PM), FZ (rectus abdominis, RA), FC (gastrocnemius muscle, GAS).

**Supplementary Fig. 9 Molecular regulatory relationships between muscle tissue co-expression modules and myofiber phenotypes.​​**

**
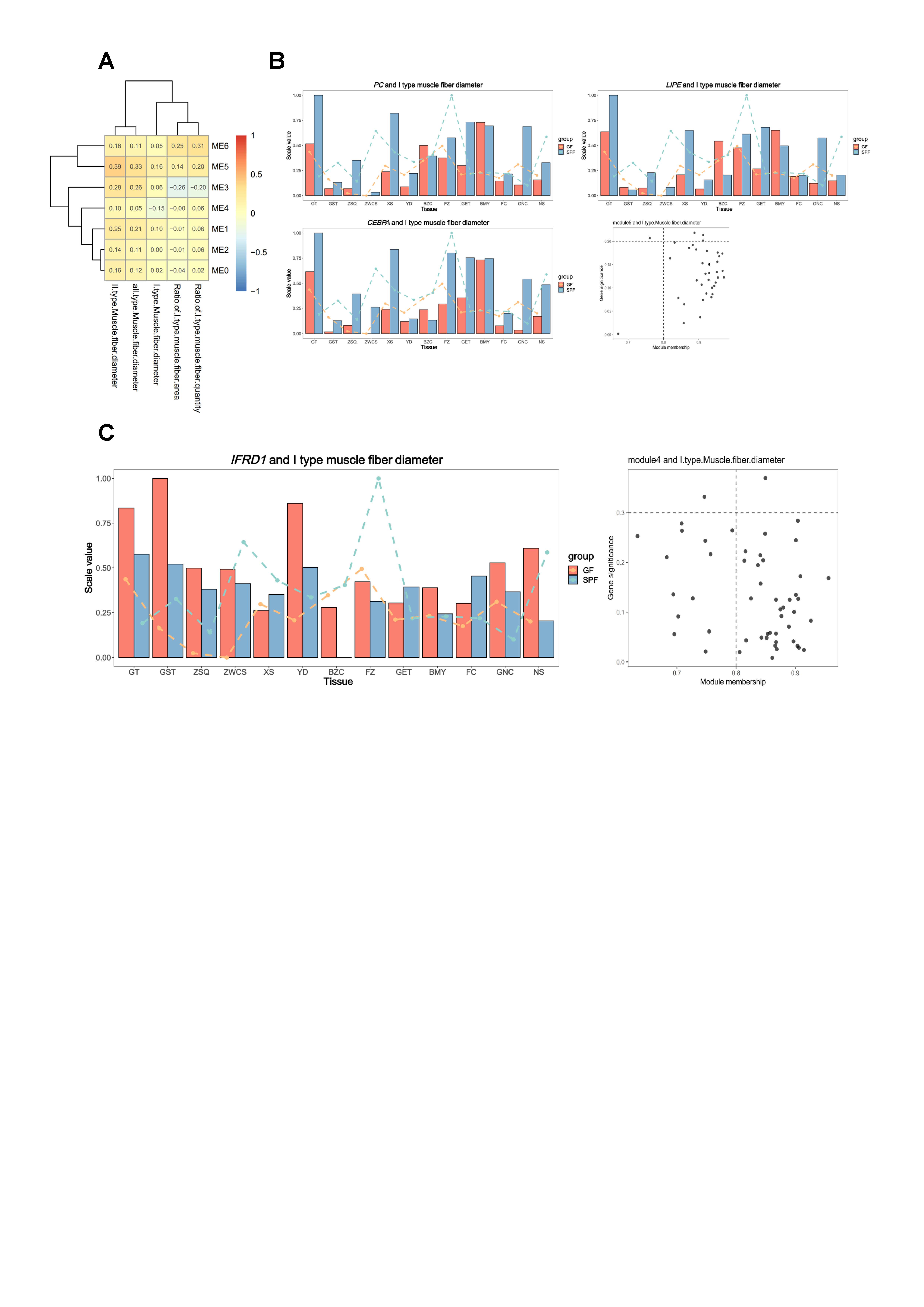
**

**Supplementary Fig. 9 Molecular regulatory relationships between muscle tissue co-expression modules and myofiber phenotypes.**​​ **(A)** Module-myofiber phenotype correlations. **(B)** Gene significance for type I myofiber diameter and M5 module member distribution. **(C)** Gene significance for type I myofiber diameter and M4 module member distribution. Muscle tissue abbreviations: GT (brachial head muscle, BH), GST (triceps brachii, TB), ZSQ (flexor digitorum profundus, FDP), ZWCS (extensor digitorum lateralis, EDL), XS (pectoralis profundus, PP), YD (psoas major muscle, PM), BZC (longissimus dorsi muscle, LDM), FZ (rectus abdominis, RA), GET (biceps femoris, BF), BMY (soleus muscle, SOL), FC (gastrocnemius muscle, GAS), GNC (medial femoral muscle, MF), NS (adductores, ADD).

**Supplementary Fig. 10 Gene significance for type II myofiber diameter and ME5 module member distribution.**

**
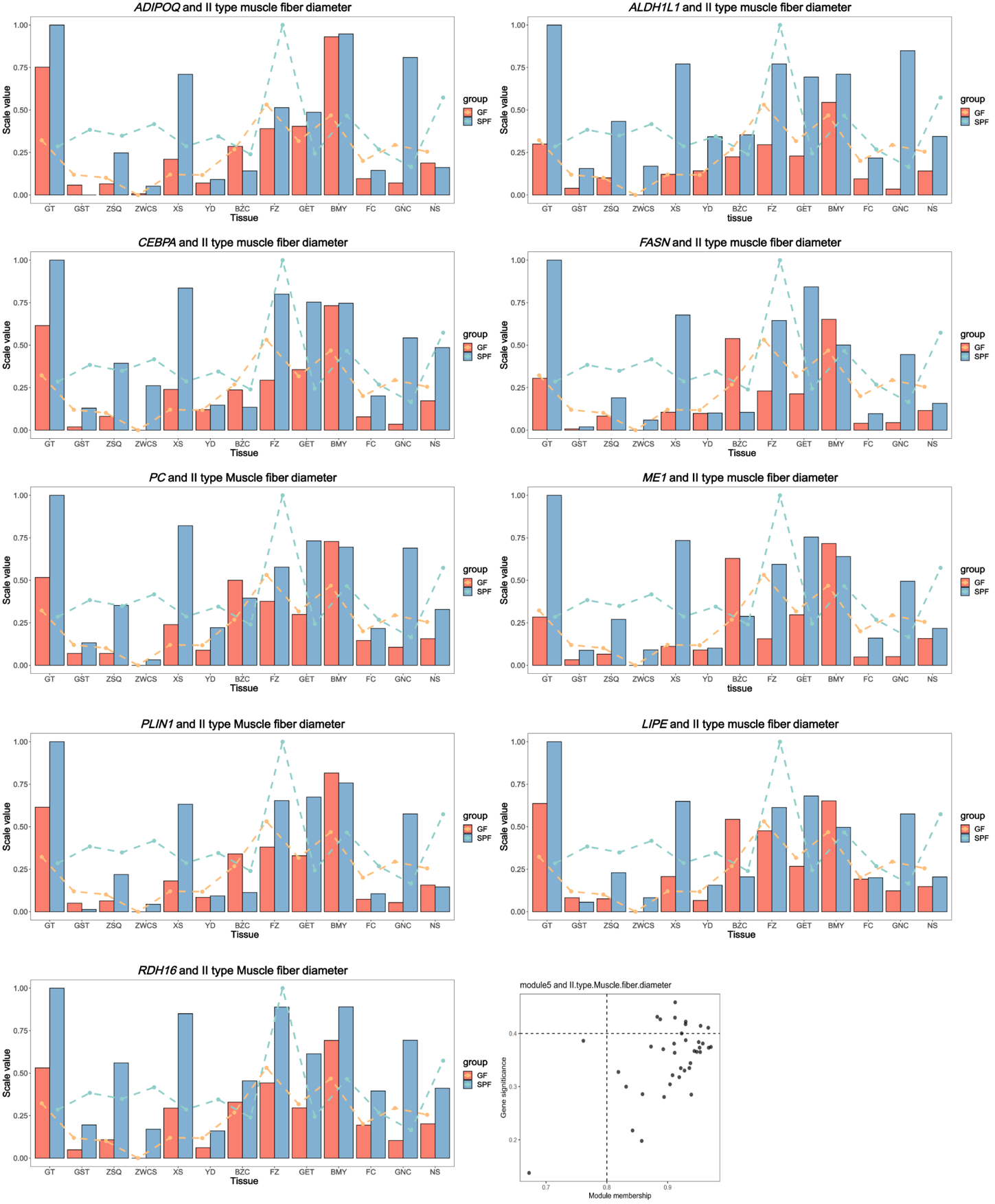
**

**Supplementary Fig. 10 Gene significance for type II myofiber diameter and ME5 module member distribution.** Muscle tissue abbreviations: GT (brachial head muscle, BH), GST (triceps brachii, TB), ZSQ (flexor digitorum profundus, FDP), ZWCS (extensor digitorum lateralis, EDL), XS (pectoralis profundus, PP), YD (psoas major muscle, PM), BZC (longissimus dorsi muscle, LDM), FZ (rectus abdominis, RA), GET (biceps femoris, BF), BMY (soleus muscle, SOL), FC (gastrocnemius muscle, GAS), GNC (medial femoral muscle, MF), NS (adductores, ADD).

**Supplementary Fig. 11 Regulatory effects of co-expression network modules on myofiber morphological characteristics in muscle tissues.**

**
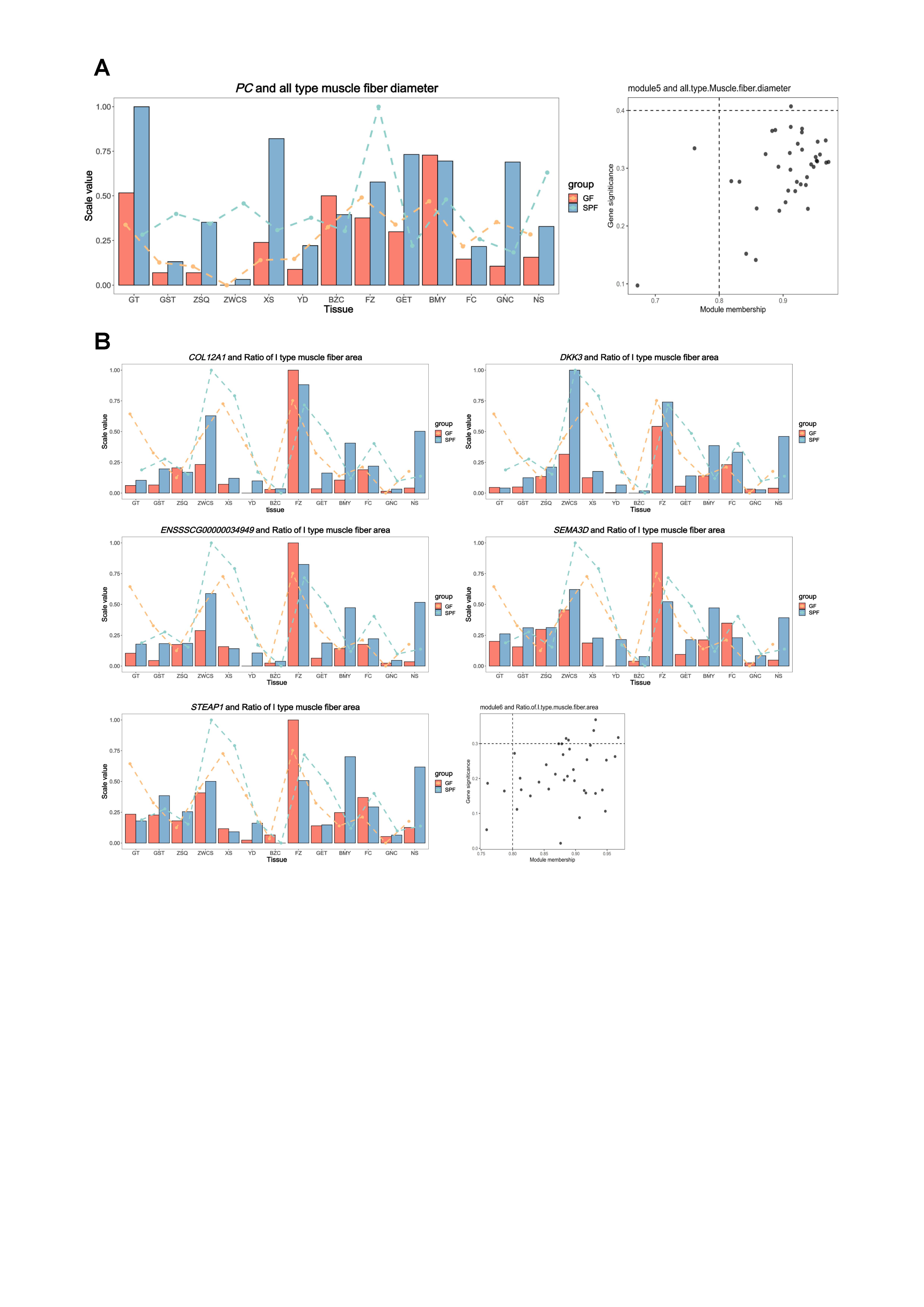
**

**Supplementary Fig. 11 Regulatory effects of co-expression network modules on myofiber morphological characteristics in muscle tissues. ​​(A)** Gene significance for total myofiber diameter and ME5 module membership distribution. **(B)** Gene significance for type I myofiber cross-sectional area ratio and ME6 module membership distribution. Muscle tissue abbreviations: GT (brachial head muscle, BH), GST (triceps brachii, TB), ZSQ (flexor digitorum profundus, FDP), ZWCS (extensor digitorum lateralis, EDL), XS (pectoralis profundus, PP), YD (psoas major muscle, PM), BZC (longissimus dorsi muscle, LDM), FZ (rectus abdominis, RA), GET (biceps femoris, BF), BMY (soleus muscle, SOL), FC (gastrocnemius muscle, GAS), GNC (medial femoral muscle, MF), NS (adductores, ADD).

**Supplementary Fig. 12 Fine-tuned regulation of type I myofiber characteristics by co-expression network modules in muscle tissues.​​**

**
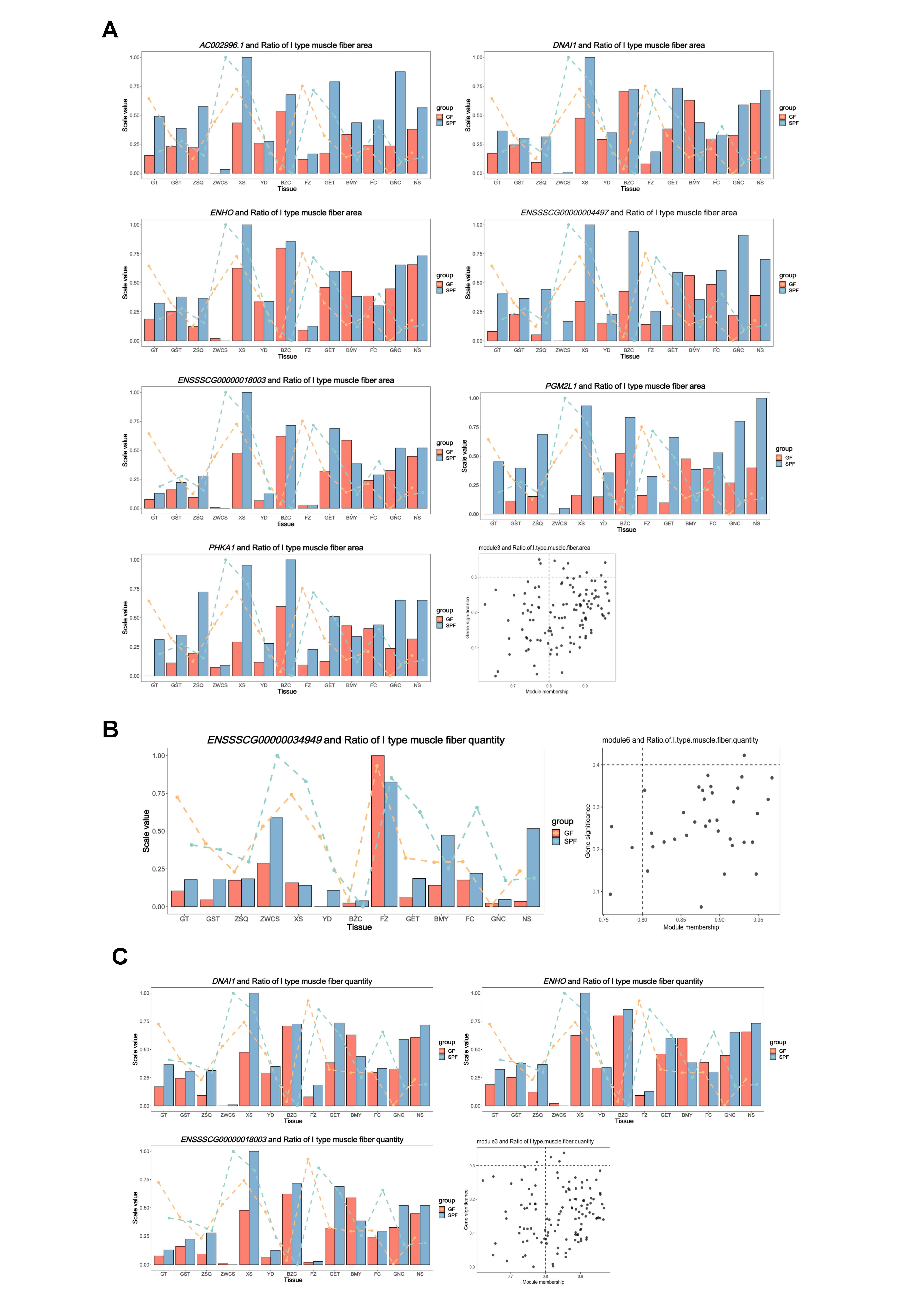
**

**Supplementary Fig. 12 Fine-tuned regulation of type I myofiber characteristics by co-expression network modules in muscle tissues.​​ (A)** Gene significance for type I myofiber cross-sectional area proportion and ME3 module membership distribution. **(B)** Gene significance for type I myofiber numerical proportion and ME6 module membership distribution. **(C)** Gene significance for type I myofiber numerical proportion and ME3 module membership distribution.
